# Development and internal validation of clinical prediction models for scrub typhus and doxycycline-treatable causes in paediatric acute encephalitis syndrome in Karnataka, India: a multicentre, prospective study

**DOI:** 10.1016/j.lansea.2025.100626

**Published:** 2025-06-26

**Authors:** Tina Damodar, Maria Jose, Uddhava V. Kinhal, Bhagteshwar Singh, Surbhi Telang, Akhila Lekha, Srilatha Marate, Namratha Prabhu, Chitra Pattabiraman, Prathyusha Parthipulli Vasuki, A.V. Lalitha, Fulton Sebastian Dsouza, Sushma Veeranna Sajjan, Gangasamudra Veerappa Basavaraja, Mallesh Kariyappa, Benedict Daniel Michael, Reeta S. Mani, Tom Solomon, Vykuntaraju K. Gowda, Vasanthapuram Ravi, Ravi Yadav, Lance Turtle, Ruwanthi Kolamunnage-Dona

**Affiliations:** a National Institute of Mental Health and Neurosciences (NIMHANS), Bengaluru, India; b Indira Gandhi Institute of Child Health, Bengaluru, India; c Institute of Infection, Veterinary, and Ecological Sciences, University of Liverpool, Liverpool, UK; d Tropical and Infectious Diseases Unit, Royal Liverpool University Hospital, Liverpool, UK; e Christian Medical College, Vellore, Tamil Nadu, India; f Liverpool School of Tropical Medicine, Liverpool, UK; g St John's Medical College and Hospital, Bengaluru, India; h Bangalore Medical College and Research Institute, Bengaluru, India; i National Institute of Health and Care Research Health Protection Research Unit in Emerging and Zoonotic Infections, University of Liverpool, Liverpool, UK; j The Walton Centre NHS Foundation Trust, Liverpool, UK; k The Pandemic Institute, Liverpool, UK; l Institute of Population Health, University of Liverpool, Liverpool, UK

**Keywords:** Acute encephalitis syndrome (AES), Scrub typhus, Doxycycline-treatable infections, Paediatric encephalitis, Clinical prediction models, Point-scoring systems

## Abstract

**Background:**

Scrub typhus and other doxycycline-treatable infections are significant contributors of acute encephalitis syndrome (AES) in India. Limited surveillance in South India has hindered their recognition and the inclusion of doxycycline in treatment protocols. We aimed to systematically investigate infectious aetiologies of AES in children from Karnataka, India, and develop clinical prediction models for diagnosing scrub typhus and guiding clinical decisions for doxycycline therapy.

**Methods:**

This multicentre, prospective study enrolled children aged >28 days to 18 years with AES presenting to three tertiary care hospitals in Bengaluru, India. Primary outcomes were microbiological diagnosis of AES and clinical prediction models for diagnosing scrub typhus and identifying patients with doxycycline-treatable causes. Models were developed using multivariable logistic regression, internally validated, and simplified into point-scoring systems. Model performance was evaluated using c-statistics, calibration slopes, and calibration-in-the-large, adhering to TRIPOD guidelines.

**Findings:**

Between February 2020 and February 2023, 714 children were screened, of whom 587 were included. Of these, 315 (54%) had a microbiological diagnosis. Scrub typhus accounted for 138/315 (44%), and doxycycline-treatable causes were diagnosed in 193/315 (61%) of these cases. Key predictors associated with both scrub typhus and doxycycline-treatable causes were age, illness duration, lymphadenopathy, oedema, hepatomegaly, lymphocyte count, platelet count, and serum albumin levels. Adjusted c-statistics were 0.83 (95% CI: 0.78–0.87) for the scrub typhus model and 0.75 (95% CI: 0.7–0.81) for the doxycycline model, with calibration slopes of 0.85 (0.82–0.88) and 0.83 (0.78–0.87), respectively. CITL values were −0.03 (−0.06–0) and 0.05 (0.02–0.09). Points-based scores predicted probabilities ranging from 5% to 99.8% (scrub typhus model) and 20%–99% (doxycycline-treatable model).

**Interpretation:**

Scrub typhus was the most common microbiological diagnosis, and most patients had a doxycycline-treatable cause, underscoring the need to prioritise doxycycline in empirical treatment protocols in South India. The models demonstrated strong performance; however external validation is necessary for broader applicability.

**Funding:**

DBT/Wellcome Trust India Alliance FellowshipIA/CPHE/18/1/503960.

**Research in context**

**Evidence before this study**

Acute encephalitis syndrome (AES), predominantly caused by infectious aetiologies, is a significant public health challenge, disproportionately affecting children in India. A systematic PubMed search was conducted using MeSH terms such as “acute encephalitis syndrome,” “children,” “aetiology,” and “India”. The search identified a few multicentre studies from northern India, which revealed diverse and region-specific aetiologies of AES, including the emergence of scrub typhus (caused by *Orientia tsutsugamushi*) as an important cause. Research in southern India was limited to single-centre studies or was focused on AES caused by specific pathogens. No large-scale multicentre prospective studies systematically evaluating children with AES in southern India were identified.

In addition, a separate systematic PubMed search conducted in October 2024 targeted cohort studies developing clinical prediction models for scrub typhus-associated AES globally, using keywords such as “scrub typhus,” “acute encephalitis syndrome,” and “clinical prediction”. Two single-centre studies from northern India proposed scoring systems to differentiate scrub typhus from other causes of AES. However, neither study adhered to recommended TRIPOD guidelines for clinical prediction model development and validation. No studies were identified that developed models to support clinical decision-making regarding doxycycline use in children with AES.

**Added value of this study**

To our knowledge, this study is the largest multicentre prospective systematic investigation for a broad range of pathogens causing AES in children in southern India, and among the largest globally. It is also the first to develop clinical prediction models for diagnosing scrub typhus and guiding clinical decision-making for doxycycline therapy in children with AES, using recommended guidelines. By leveraging carefully collected prospective clinical data, the study translates its findings into practical, user-friendly point-scoring systems, ready for real-world application following external validation.

**Implications of all the available evidence**

The findings of this study offer a strong evidence base supporting the inclusion of scrub typhus testing in diagnostic algorithms for AES surveillance in southern India. They further advocate for the incorporation of doxycycline in standard empirical treatment guidelines for AES. The clinical prediction models developed in this study hold significant potential for improving diagnosis and guiding doxycycline therapy, especially in low-resource settings with limited microbiological diagnostic capacity.

## Introduction

Acute encephalitis syndrome (AES), characterised by acute-onset fever and altered mental status and/or new seizures, presents a significant global public health challenge. It has an incidence of 3.5 to 13.8 cases per 100,000 patient-years, disproportionately affects children, and is associated with high morbidity and mortality.^1^, ^2^, ^3^

In India, ∼10,000 cases of AES are reported annually to the National Vector Borne Disease Control Programme (NVBDCP), though this is likely an underestimate.^3^ Historically, surveillance efforts have prioritised Japanese encephalitis (JE), even as its incidence has significantly declined due to widespread vaccination.^4^ Moreover, recent studies reveal a dynamic, region-specific, and evolving aetiology of AES in India.^3^^,^^5^ Scrub typhus (caused by *O. tsutsugamushi*), an under-recognised cause of AES, has been increasingly reported in certain regions.^6^, ^7^, ^8^

Currently, third-generation cephalosporins and acyclovir remain the cornerstone of empirical AES treatment regimens for children in India.^9^ While cephalosporins treat several bacterial pathogens linked to AES, widespread immunisation has reduced their incidence.^3^^,^^10^ The low incidence of Herpes simplex encephalitis and potential risks of therapy are prompting re-evaluation of the routine empirical use of acyclovir in paediatric AES.^11^

Doxycycline, a cost-effective and efficacious treatment for scrub typhus, leptospirosis, and other rickettsial infections, has emerged as a crucial therapeutic option.^5^^,^^6^^,^^12^, ^13^, ^14^ However, despite growing recognition of these doxycycline-treatable causes, it remains absent from standard treatment guidelines of AES, largely due to overlapping clinical features with other causes of AES, limited surveillance, and diagnostic challenges.^3^^,^^8^^,^^9^^,^^14^, ^15^, ^16^ The aim of this study was to systematically investigate infectious causes of AES in children in South India and to develop clinical prediction models for diagnosing scrub typhus and guiding clinical decisions for doxycycline therapy.

## Methods

### Study design and participants

This multicentre, observational, prospective study screened children with suspected AES at three tertiary care hospitals in Bengaluru, Karnataka, South India: (i) St. John's Medical College and Hospital, (ii) Indira Gandhi Institute of Child Health (IGICH), and (iii) Vani Vilas Hospital, Bangalore Medical College; between Feb 14, 2020, and Feb 28, 2023. Details of referral hospitals are in Supplementary Information (pp 1–2). Screening occurred consecutively in emergency departments, inpatient wards, and intensive care units, to ensure that all eligible children presenting to the participating hospitals during the study period were included.

Inclusion criteria were based on the Indian NVBDCP and WHO AES case definitions.^17^ Briefly, children aged >28 days to 18 years were included if they presented with fever or a history of febrile illness and an altered mental state (e.g., irritability, drowsiness, confusion, disorientation, personality or behavioural changes, altered speech, irrelevant talk, or decreased/altered consciousness) lasting ≥24 h, with or without new onset seizures, and an illness duration of less than 30 days.

Pre-defined exclusion criteria included onset of symptoms during the neonatal period (first 28 days of life), simple febrile seizures, postoperative or post-traumatic seizures, documented immunocompromised conditions, and a prior AES episode within the past year. In addition, children who were diagnosed during hospitalisation with a confirmed non-infectious or post-inflammatory cause of AES, or with an immunocompromising disease, were subsequently excluded from the study.

The study was approved by the institutional ethics and review boards of the participating hospitals and the coordinating centre, National Institute of Mental Health & Neurosciences (NIMHANS). Fully informed consent was obtained from caregivers, with assent from older children, facilitated by a specially trained study team using procedures and forms approved by the institutional ethics committees.

### Procedures

Clinical data documented by the treating physician at hospital admission, along with baseline routine biochemical and haematological test results from referral hospitals, were recorded. Socio-demographic details were captured through caregiver interviews (Supplementary Information, p 2).

**Fig. 1:**
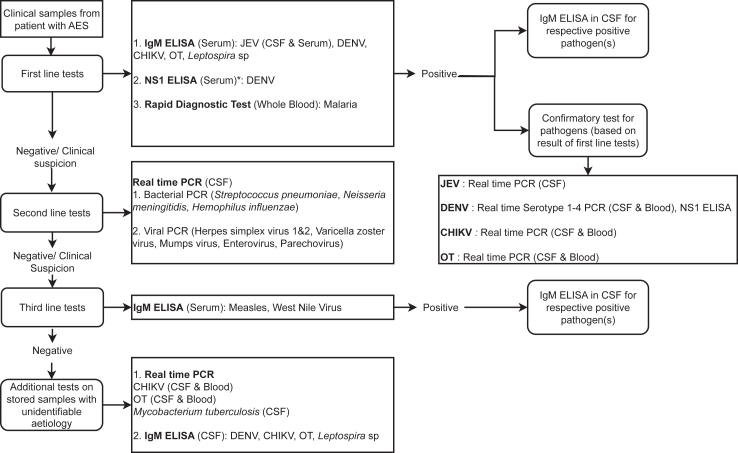
Diagnostic algorithm used for serologic and molecular testing of samples from children with acute encephalitis syndrome, in Bengaluru, India. AES: Acute encephalitis syndrome, JEV: Japanese encephalitis virus, DENV: Dengue virus, CHIKV: Chikungunya virus, OT: *Orientia tsutsugamushi*, CSF: Cerebrospinal fluid, NS1: Nonstructural Protein 1. ∗Dengue NS1 ELISA was be performed on serum samples from children with ≤7-day illness duration.

Blood and cerebrospinal fluid (CSF) samples were sent to and tested at NIMHANS, Bangalore, using a three-tier diagnostic algorithm (Fig. 1). Briefly, first-line tests targeted JEV, *O. tsutsugamushi*, dengue virus, chikungunya virus, *Leptospira* species, and malarial parasites. Second-line tests were performed if first-line results were negative or if there was clinical suspicion for other pathogens, including Herpes simplex virus (HSV)-1 & −2, Varicella zoster virus (VZV), mumps virus, enterovirus, and parechovirus. Third-line tests, including for West Nile virus (WNV) and measles virus, were conducted following negative second-line results or in case of clinical suspicion. Full details of the testing protocols are provided in the Supplementary Information (pp 2–8).

Results of first-line tests were provided to clinicians within 24–48 h, while second- and third-line test results were shared within 48–72 h, from receipt of samples. In April 2022, the algorithm was revised based on results from the first two years of the study to include real-time PCR (cerebrospinal fluid [CSF] and blood) for *O. tsutsugamushi* and chikungunya as first-line tests. Additionally, the results of any routine microbiological tests, if conducted, were also obtained from the recruitment hospitals.

### Outcomes

Primary outcomes were microbiological diagnosis of AES and clinical prediction models for diagnosing scrub typhus and identifying cases with doxycycline-treatable causes.

Microbiological diagnosis, based on comprehensive pathogen testing, was classified as confirmed, probable, or possible according to predefined pathogen causation criteria.^18^ For model development, scrub typhus diagnoses included confirmed or probable cases without microbiological evidence of another pathogen. Comparators consisted of confirmed, probable, or possible aetiologies other than scrub typhus. Cases with possible scrub typhus or rickettsial infections were excluded. Cases with doxycycline-treatable causes included confirmed, probable, or possible cases of scrub typhus, leptospirosis, or any rickettsial infection. Comparators comprised confirmed, probable, or possible aetiologies other than these. Cases with unidentified aetiologies were excluded from both models. Neurological functional outcomes were assessed by telephone three months post-discharge using the Liverpool Outcome Score, relying on caregiver proxy reporting, age-appropriate functional tasks, and comparison with baseline functioning to assess post-illness neurological status.^18^^,^^19^ Scores of 1, 2, or 3 were categorised as unfavourable outcomes, while scores of 4 or 5 indicated favourable outcomes. Secondary outcomes were comparisons of clinical and laboratory findings across different aetiologies and assessments of neurological functional outcomes.

### Analysis

Categorical variables were summarised as proportions. The distribution of continuous variables was assessed using the Shapiro–Wilk test and further verified through visual inspection of histograms for normality. Continuous variables were described as median (IQR) for skewed data or mean (SD) for normally distributed data. Comparisons between groups were conducted using the χ^2^ test (or Fisher’s exact test for smaller event counts) for categorical variables, and the Mann–Whitney U test for skewed continuous variables. A p-value < 0.05 was considered statistically significant.

For development of the clinical prediction models, we adhered to the Transparent Reporting of a Multivariable Prediction Model for Individual Prognosis or Diagnosis (TRIPOD) guidelines.^20^

Multivariable logistic regression was used for identifying characteristics associated with scrub typhus-associated AES and presence of doxycycline-treatable causes of AES. Potential predictors obtained at hospital admission were preselected by a team of clinical investigators (TD, UVK, BS, VKG, RY, LT), based on clinical relevance, prior evidence and accessibility of tests. These included clinical variables such as age, illness duration (before presentation to hospital), regional lymphadenopathy, oedema, rash, hepatomegaly, and signs of meningeal irritation. Laboratory variables included baseline peripheral blood lymphocyte count, platelet count, and serum albumin.

Primary models for scrub typhus and doxycycline-treatable causes of AES were developed using these preselected variables. Two additional versions of each model were developed: a “presentation-at-hospital model”, which used only the preselected clinical variables, for use at patient’s presentation to the hospital; and a “post-lumbar puncture (post-LP) model” that incorporated CSF lymphocyte count and CSF protein concentration alongside the preselected clinical and laboratory variables.

Missing data for selected variables were multiply imputed using the predictive mean-matching method via chained equations, generating 20 imputed datasets. Appropriate functional forms of continuous variables were determined using fractional polynomials within each imputed dataset. Backward selection (using a p value threshold of <0.1) was applied to identify variables for inclusion in the final model, ensuring that no two predictors with known interdependencies were included together, to minimise collinearity and confounding. Variables retained in at least 10 of the 20 models were included. Pooled odds ratios (ORs) and intercepts were calculated using Rubin’s rule.^21^^,^^22^

Model performance was evaluated using discrimination (c-statistic) and calibration (calibration slopes [c-slopes] and calibration-in-the-large [CITL]). Briefly, c-statistics measure the model’s ability to differentiate cases from non-cases, with values closer to 1 indicating stronger discrimination. C-slopes assess calibration, with a value of 1 indicating perfect agreement between predicted and observed probabilities. Lower values suggest overfitting. CITL values reflect systematic miscalibration, where negative values indicate overestimation of risk, and positive values indicate underestimation. When average predictions from the model are well-calibrated with observed outcomes, CITL is 0.^23^ Median c-statistics and calibration metrics across the imputed datasets were reported. Calibration plots were generated for each dataset, with median estimates presented.

Internal validation was conducted using non-parametric bootstrapping with 1000 bootstrap samples to estimate optimism and assess model stability. For each bootstrap sample, predictor selection and model building were repeated, and the apparent model performance in the bootstrap sample was compared to its performance in the original dataset. Optimism-corrected (or adjusted) performance metrics were calculated. To mitigate overfitting, penalisation was applied using the optimism-adjusted c-slope as a shrinkage factor in the final model.^21^^,^^22^ The final optimism-adjusted prediction models were transformed into point scoring systems by assigning integer values to coefficients and predicted probabilities were obtained.^24^ All statistical analyses were performed using R version 4.4.2 (The R Foundation for Statistical Computing, Vienna, Austria).

### Role of the funding source

The study was by DBT/Wellcome Trust India Alliance Fellowship IA/E/18/1/503960 awarded to TD. The funders had no role in study design, data collection and analysis, decision to publish, or preparation of the manuscript.

## Results

**Fig. 2:**
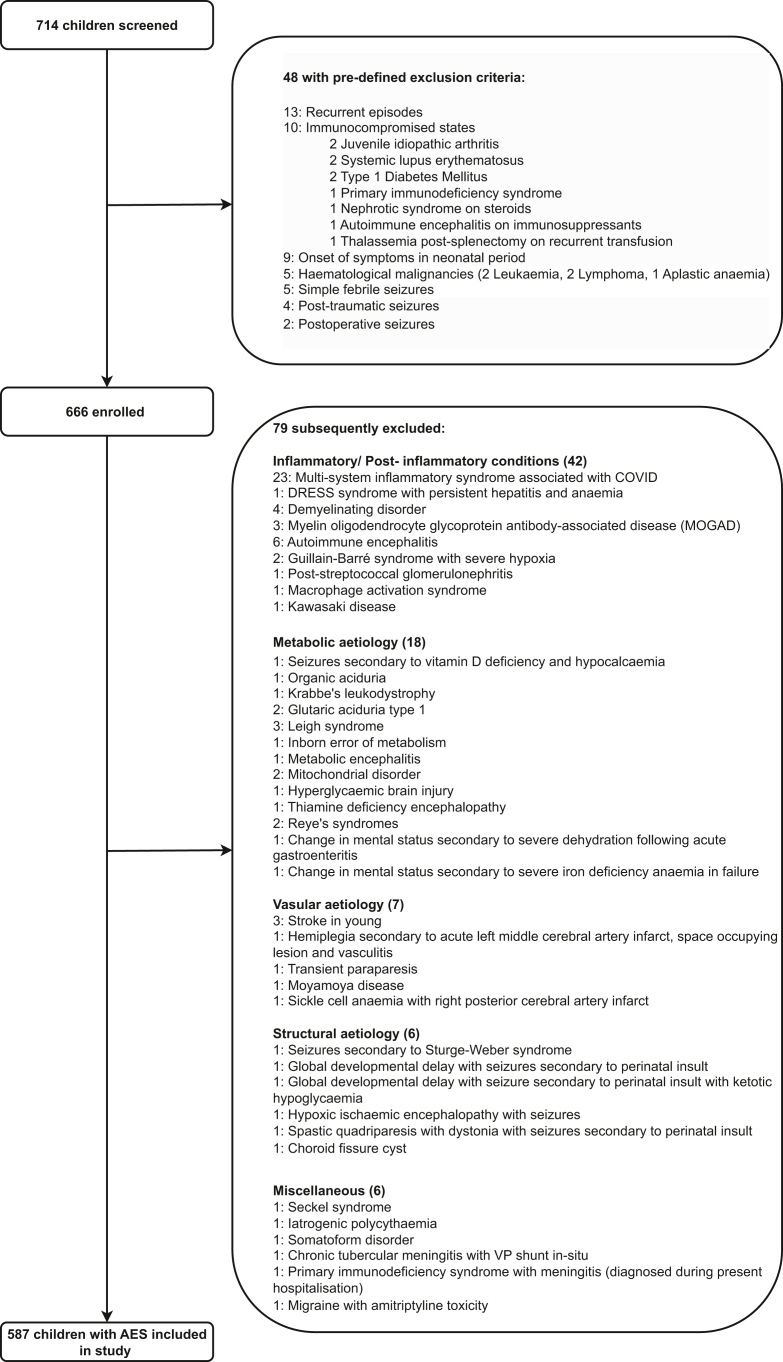
Study flow diagram. AES: Acute encephalitis syndrome, DRESS: Drug Reaction with Eosinophilia and Systemic Symptoms.

Of 714 children screened between Feb 14, 2020, and Feb 28, 2023, 48 were excluded based on predefined exclusion criteria. Of the remaining 666, 79 were subsequently excluded due to alternate diagnoses. Ultimately, 587 children were included in the study (Fig. 2, Supplementary Information, p 9).

A microbiological diagnosis was established in 315 (54%) of 587 children (Table 1). *O. tsutsugamushi* (scrub typhus) was the most common pathogen, defined as a confirmed or probable aetiology in 138/315 (44%), confirmed or probable Japanese encephalitis virus (JEV) infection was identified in 36/315 (11%), dengue virus in 30/315 (10%), *Leptospira* species in 17/315 (5%), and chikungunya virus in 16/315 (5%). Other causative aetiologies included HSV and measles virus in 5 (2%) each, VZV in 4 (1%), enterovirus and *Streptococcus pneumoniae* in 3 (1%) each, mumps virus in 2 (0.6%), and single cases (0.3%) of *Haemophilus influenzae*, *O. tsutsugamushi* and chikungunya co-infection, and of chikungunya and dengue virus co-infection.

**Table 1:** Aetiological distribution of cases with an identifiable infectious aetiology (n = 315).

Microbiological diagnosis	No. patients (%)
*Orientia tsutsugamushi*	138 (43.8)
Japanese encephalitis virus	36 (11.4)
Dengue virus	30 (9.5)
Leptospira sp	17 (5.4)
Chikungunya virus	16 (5.1)
Measles virus	5 (1.6)
Varicella Zoster virus	4 (1.3)
Typhus group *Rickettsia* sp^a^	4 (1.3)
*Mycobacterium tuberculosis*^a^	4 (1.3)
Herpes simplex virus-1	4 (1.3)
*Streptococcus pneumoniae*	3 (1.0)
Enterovirus	3 (1.0)
Mumps virus	2 (0.6)
SARS-CoV2	1 (0.3)
*Salmonella typhi*^a^	1 (0.3)
Methicillin-resistant *Staphylococcus aureus*^a^	1 (0.3)
Herpes simplex virus-2	1 (0.3)
*Haemophilus influenzae*	1 (0.3)
Chikungunya virus & Dengue virus	1 (0.3)
*Orientia tsutsugamushi* & Chikungunya	1 (0.3)
Multiple pathogens	42 (13.3)
**Total identifiable infectious aetiologies**	**315 (100.0)**

a Microbiological diagnosis based on tests performed at referral hospital.

Microbiological testing conducted at referring hospitals further identified *Mycobacterium tuberculosis* and *Rickettsia* species in 4 (1%) each, and single cases (0.3%) of methicillin-resistant *Staphylococcus aureus*, *Salmonella typhi*, and SARS-CoV-2. Probable and/or possible causation by multiple pathogens was identified in 42 (13%) of 315 microbiologically diagnosed cases (Supplementary Information, p 9). The aetiology remained unidentified in 272 (46%) of the 587 children. State and district-wise distribution of cases are shown in Supplementary Information, pp 10–15. Age, year and month-wise distribution of aetiologies are shown in Supplementary Information, pp 16–18.

Clinical features, demographics, and outcomes are detailed in Supplementary Information (pp 19–28). Briefly, the median age was 5 (IQR 2–10) years, with a relative male predominance (58%). Median illness duration before referral hospital presentation was 5 (3–7) days, and 370/587 (63%) were referred from other facilities, where they received at least one dose of antibiotics—most commonly third-generation cephalosporins, with or without other antimicrobials. The median hospitalisation duration was 9 (7–14) days. Among 434/587 (74%) with available Liverpool Outcome Scores, 51 (12%) died (score 1), 34 (8%) had severe sequelae (score 2), 76 (18%) moderate sequelae (score 3), 40 (9%) mild sequelae (score 4), and 233 (54%) achieved complete recovery (score 5).

Among 315 children with microbiological diagnosis, 271 (86%) were included in developing models for diagnosing scrub typhus—127 (40%) with scrub typhus (and without microbiological evidence of other pathogens) and 144 (46%) with other aetiologies (Supplementary Information, pp 29). Children with scrub typhus were older (median 8 vs. 5.5 years, p = 0.003) and had a longer illness duration before hospitalisation (median 6 vs. 4 days, p < 0.001). Eighty-six of 138 (62%) children with scrub typhus received third-generation cephalosporins prior to admission at the referral centre. Lymphadenopathy (17.3% vs. 2.8%, p < 0.001), hepatomegaly (52.8% vs. 22.2%, p < 0.001), and oedema (24.4% vs. 6.9%, p < 0.001) were more frequent in scrub typhus. Laboratory findings showed lower median platelet counts (110.5 × 10^9^/L vs. 239 × 10^9^/L, p < 0.001), lower albumin (2.8 vs. 3.2 mg/dl), and higher CSF protein (57.5 vs. 25.7 mg/dl). Transaminitis (68% vs. 28%, p < 0.001) and CSF pleocytosis (64% vs. 37%, p < 0.001) were more common in scrub typhus (Supplementary Information, pp 30–33). Among 114 children with Liverpool Outcome Scores, 45 (39%) either died (score = 1) or experienced neurological sequelae (score 2–4) (Supplementary Information, pp 24).

All 315 were included in the model for identifying children with doxycycline-treatable causes—193 (61%) with doxycycline-treatable causes and 122 (39%) without (Supplementary Information, pp 34–35). Children with doxycycline-treatable causes had clinical and laboratory findings similar to those with scrub typhus, described above (Supplementary Information, pp 36–39). Proportions of missing data for preselected potential predictors included in the model development ranged between 0 and 10% (Supplementary Information, pp 40–41). Unadjusted associations between these variables and outcomes are shown in Supplementary Information, pp 42.

The final pool of predictors in the developed primary models—selected based on significance in backward selection (p < 0.1) and inclusion in at least 10 of 20 imputed datasets—comprised age, illness duration, oedema, regional lymphadenopathy, hepatomegaly, peripheral blood lymphocyte count, platelet count, and serum albumin levels (Table 2). These models were simplified into point-scoring systems, with predicted probabilities of outcomes ranging from 5% to 99.8% for scores between 0 and 18 in the scrub typhus diagnosis model and from 20% to 99% for scores between 0 and 12 in the model for diagnosing doxycycline-treatable causes of AES (Tables 3 and 4). Presentation-at-hospital and post-LP models are detailed in Supplementary Information pp 43–48. Model performances are summarised in Supplementary Information (p 49).

**Table 2:** Multivariable models adjusted for shrinkage.

Predictor	Comparison	Adjusted Odds ratio (95% CI)
**Primary model for scrub typhus**		
Intercept, log odds ratio (S.E.)		1.37 (0.82)
Age (years)	<6	1.00 (reference)
	≥6	1.26 (1.10–1.44)
Illness duration (days)	≤5	1.00 (reference)
	>5	1.15 (0.97–1.36)
Oedema	No	1.00 (reference)
	Yes	2.20 (0.95–5.06)
Lymphadenopathy	No	1.00 (reference)
	Yes	5.74 (1.94–16.99)
Hepatomegaly	No	1.00 (reference)
	Yes	2.67 (1.15–6.19)
Lymphocyte count (×10ˆ9/L)	<1.5	1.00 (reference)
	1.5-3	1.21 (1.11–1.32)
	>3	2.03 (1.46–2.82)
Platelet count (×10ˆ9/L)	≤150	1.04 (1.00–1.08)
	>150	1.00 (reference)
Serum albumin (mg/dL)	≤3	1.64 (1.18–2.29)
	3-3.5	1.26 (1.08–1.47)
	>3.5	1.00 (reference)
**Primary model for doxycycline-treatable causes**		
Intercept, log odds ratio (S.E.)		1.12 (0.73)
Age (years)	<6	1.00 (reference)
	≥6	1.24 (1.10–1.39)
Illness duration (days)	≤5	1.00 (reference)
	>5	1.17 (1.01–1.35)
Oedema	No	1.00 (reference)
	Yes	1.82 (0.87–3.80)
Lymphadenopathy	No	1.00 (reference)
	Yes	4.09 (1.41–11.83)
Hepatomegaly	No	1.00 (reference)
	Yes	1.52 (0.90–2.56)
Lymphocyte count (×10ˆ9/L)	≤3	1.00 (reference)
	>3	1.37 (1.11–1.69)
Platelet count (×10ˆ9/L)	≤150	1.04 (1.01–1.07)
	>150	1.00 (reference)
Serum albumin (mg/dL)	≤3.5	1.23 (0.97–1.56)
	>3.5	1.00 (reference)

S.E. = standard error.

**Table 3:** Scoring system for probability of diagnosis of scrub typhus and doxycycline-treatable causes in children with AES.

Predictor	Category	Point score
Primary model for scrub typhus		
Age (years)	<6	0
	≥6	2
Illness duration (days)	≤5	0
	>5	1
Oedema	No	0
	Yes	2
Lymphadenopathy	No	0
	Yes	3
Hepatomegaly	No	0
	Yes	2
Lymphocyte count (×10ˆ9/L)	<1.5	0
	1.5-3	1
	>3	4
Platelet count (×10ˆ9/L)	≤150	2
	>150	0
Serum albumin (mg/dL)	≤3	2
	3-3.5	1
	>3.5	0
Primary model for doxycycline-treatable causes		
Age (years)	<6	0
	≥6	2
Illness duration (days)	≤5	0
	>5	1
Oedema	No	0
	Yes	1
Lymphadenopathy	No	0
	Yes	3
Hepatomegaly	No	0
	Yes	1
Lymphocyte count (x10ˆ9/L)	≤3.5	0
	>3	2
Platelet count (x10ˆ9/L)	≤150	1
	>150	0
Serum albumin (mg/dL)	≤3.5	1
	>3.5	0

**Table 4:** Estimate of probability based on scores in primary models.

Scoring	Estimate of probability of scrub typhus in children with AES (%)	Estimate of probability of doxycycline-treatable causes of AES (%)
0	5.4	20.4
1	8.6	29.7
2	13.4	41.1
3	20.4	53.5
4	29.7	65.5
5	41.0	75.8
6	53.4	83.7
7	65.4	89.5
8	75.7	93.3
9	83.7	95.8
10	89.4	97.4
11	93.3	98.4
12	95.8	99.0
13	97.4	–
14	98.4	–
15	99.0	–
16	99.4	–
17	99.6	–
18	99.8	–

The primary model for scrub typhus showed an adjusted c-statistic of 0.83 (95% CI: 0.78–0.87) with an adjusted c-slope of 0.85 (0.82–0.88) and CITL of −0.03 (−0.06–0.00). The presentation-at-hospital model had an adjusted c-statistic of 0.73 (0.67–0.79), with a c-slope of 0.88 (0.83–0.92) and CITL of −0.02 (−0.05–0.01). The post-LP model, which incorporated CSF parameters, had an adjusted c-statistic of 0.84 (95% CI: 0.79–0.88), an adjusted c-slope of 0.83 (0.80–0.86), and CITL of −0.03 (−0.07–0.01) (Supplementary Information pp 49).

For doxycycline-treatable causes, the primary model achieved an adjusted c-statistic of 0.75 (95% CI: 0.70–0.81), with an adjusted c-slope of 0.83 (0.78–0.87) and CITL of 0.05 (0.02–0.09). The presentation-at-hospital model had an adjusted c-statistic of 0.70 (0.64–0.76), with an adjusted c-slope of 0.86 (0.80–0.92) and CITL of 0.05 (0.01–0.09). The post-LP model had an adjusted c-statistic of 0.78 (95% CI: 0.73–0.83), an adjusted c-slope of 0.81 (95% CI: 0.77–0.85), and CITL of 0.05 (95% CI: 0.01–0.09). Calibration plots for all models are presented in Supplementary Information pp 50–52.

## Discussion

Our findings indicate a diverse aetiology of AES in children in the study, the most common being scrub typhus (138/587, 24%). Unlike countries in the western part of the globe, where sporadic encephalitis causes (e.g., Herpes simplex virus) predominate, vector-borne pathogens such as *O. tsutsugamushi*, JEV, dengue virus, chikungunya virus, and *Leptospira* spp., are major causes of AES in India and southeast Asia, as observed in our study.^3^^,^^18^^,^^25^, ^26^, ^27^, ^28^ In contrast to a study from the Greater Mekong region, where JEV caused nearly half (216/425, 51%) of childhood encephalitis cases, our study detected JEV in ∼11% of cases with an identifiable aetiology, likely reflecting the impact of widespread vaccination in India.^5^^,^^29^

Most AES cases with identified pathogens in this study were diagnosed with doxycycline-treatable pathogens (193/315, 61%), predominantly *O. tsutsugamushi*, highlighting the need for heightened awareness and improved diagnostics, particularly in regions where doxycycline not routinely included in empirical treatment. These findings should also prompt consideration of empiric doxycycline for AES in children in South India.

Nearly 40% of children with scrub typhus either died or experienced neurological sequelae, despite appropriate treatment at referral centres. Poor outcomes are often driven by delayed presentation [median (IQR): 6 (4–7) days in this study] and late initiation of appropriate antibiotics, typically after disease progression.^30^, ^31^, ^32^ Rapid and lifesaving responses to doxycycline highlight the critical importance of its early empirical use, as demonstrated in recently established AES protocols in Uttar Pradesh (northern India).^32^, ^33^, ^34^ Following this study, doxycycline has been incorporated into AES treatment protocols at all three referral hospitals.

To support diagnosis and management, this study developed and internally validated clinical prediction models to diagnose scrub typhus-associated AES and guide doxycycline therapy in children. The primary model for scrub typhus demonstrated excellent discrimination (c-stats: 0.83), reflecting its ability to differentiate scrub typhus cases from other AES causes. It had good calibration though slightly overfitted (c-slope: 0.85), with slightly overestimated predictions overall (CITL: −0.03). The presentation-at-hospital model exhibited acceptable discrimination (c-stats: 0.73) and strong calibration (c-slope: 0.88; CITL: −0.02), while the post-LP model, demonstrated excellent discrimination (c-stats: 0.84) and robust calibration (c-slope: 0.83, CITL: −0.03), offering more precise predictions when CSF data are available.^23^

Recognising that LP and CSF analysis can be delayed or difficult to perform, particularly in resource-limited settings, we developed primary models using clinical features and routinely accessible laboratory variables, allowing timely probability estimates to guide decision-making while awaiting CSF results. Presented as point scores, the models are simple to use and interpret. For instance, a three-year-old child (meeting inclusion criteria) with regional lymphadenopathy alone (score = 3) would have a 20% estimated probability of scrub typhus, while an eight-year-old child (score = 2) with a one-week illness duration (score = 1) and a lymphocyte count of 3 ( × 10^9^/L) (score = 4) would have a total score of 7, corresponding to a 65% estimated probability using the primary scrub typhus model.

The models for doxycycline-treatable causes demonstrated moderate discrimination (c-stats: 0.75, 0.70, 0.78 for primary, presentation-at-hospital, and post-LP models, respectively) and acceptable calibration, with a slight underestimation of risk (CITL: 0.05). A 20% baseline probability at a score of 0 using the primary model, likely stems from an overall high prevalence of doxycycline-treatable causes in this population, some of which share overlapping clinical features with other AES causes, as well as referral bias, where undiagnosed doxycycline-treatable cases are more likely to be referred to tertiary centres. While this may lead to some over-treatment with doxycycline, the potential harm is thought to be less common in children with increased use in recent years, and therefore balanced out by the risks of poor outcome with under-treatment and delayed therapy.^13^^,^^32^ We did not systematically collect data on adverse effects related to doxycycline use during the study, although no side effects were mentioned in discharge summaries. Additionally, the moderate performance may also be partly attributable to the limited number of data points at lower scores, reducing model precision in these ranges.^21^ A lower probability of doxycycline-treatable causes using the post-LP model could provide clinicians with valuable guidance for discontinuing doxycycline in cases with confirmed alternative diagnoses or contraindications.

A few studies have compared scrub typhus with other AES causes, but only two have proposed prediction scores.^35^, ^36^, ^37^, ^38^, ^39^ Alam and colleagues developed a scoring system differentiating scrub typhus from dengue encephalitis in children using total leukocyte count >10,000/mm^3^, pneumonia, and absence of myalgia and petechiae.^38^ However, diagnosis of pneumonia likely relied on clinical and radiographic interpretation, which can vary significantly across settings and may introduce observer bias. Subtle findings like petechiae can be overlooked particularly in populations with diverse skin tones or among clinicians with varying levels of experience. The model’s generalisability is further limited in populations like ours where respiratory and musculoskeletal symptoms are less commonly observed.^40^

Another study introduced a machine-learning-based scrub typhus encephalitis assessment tool with fever duration, neutrophil-to-lymphocyte ratio, and CSF protein, as key predictors.^39^ The tool benefits from being developed using a large cohort but was limited to a single centre in Uttar Pradesh (north India). Additionally, the complexity of machine-learning models, requiring specialised tools and skills for interpretation and validation, and their reduced transparency and reproducibility—especially when not aligned with recommended guidelines—are limitations compared to logistic regression models.^20^^,^^41^

Our models align with the scrub typhus encephalitis assessment tool by identifying similar predictors, while offering practical, user-friendly point-scoring systems with actionable probabilities tailored for children. Designed with simple variables, they address both scrub typhus diagnosis and doxycycline treatment decisions while adhering to recommended guidelines and could be suitable for diverse, resource-limited settings in India and southeast Asia with similar AES aetiological patterns.^20^^,^^26^, ^27^, ^28^^,^^42^

This study has some limitations. Its focus on tertiary care hospitals may have introduced referral bias, potentially influencing the mix of AES aetiologies, though this also ensured comprehensive clinical and laboratory data. Delayed microbiological sampling and prior antibiotic use in the majority likely contributed to the 46% (272/587) unidentifiable cases, though this proportion is consistent with or lower than other recent studies.^3^^,^^7^^,^^29^ Certain pathogens, such as *S. pneumoniae*, *H. influenzae*, HSV, and enterovirus, were underrepresented, limiting model applicability in regions where these are more common. However, *H. influenzae* and HSV are now recognised as rare in post-neonatal children globally.^3^^,^^11^ Study recruitment coincided with the onset of the COVID-19 outbreak, limiting admissions across study hospitals and constraining recruitment and variable selection for multivariable models, introducing potential biases due to sample size.^21^ Nevertheless, this remains one of the largest prospective AES studies in children, offering critical insights and a foundation for future validation studies. Variability in age provided a broad distribution to determine aetiologies across age groups but may have influenced biochemical and haematological parameters, as normal reference ranges can vary across different paediatric age groups.^43^ Similarly, pre-admission interventions (e.g., fluids) at referring hospitals could have altered biochemical parameters like serum sodium, leading to the exclusion of sodium from the list of potential predictors. Likewise, prior antibiotic use may have modified AES presentation and impacted model performance. While we prioritised the selection of variables less influenced by recent antibiotic use, residual confounding remains a limitation. However, the diagnostic approach in this study relied on several methods less likely to be affected by antibiotics, such as IgM antibody assays and viral PCR assays. Furthermore, this study may reflect the South Indian paediatric population as encountered in routine clinical practice, and the test performance observed here mirrors expectations in everyday healthcare settings.

While point-scoring systems improve usability, they provide approximate estimates compared to full models, which may impact predictive accuracy.^24^ However, selected predictors align with existing literature, and point scoring improves clinical utility. All participants were enrolled at three tertiary care hospitals in Bengaluru, even though referrals came from Karnataka, Andhra Pradesh, and Tamil Nadu, introducing potential sampling bias. Without external validation, the models should be considered context-specific and not extrapolated to the wider South Indian paediatric AES population. Future work will focus on validating both prediction models, along with their simplified integer score versions, using external data from similar AES studies. Following this, clinical utility of the models, for example for withholding or stopping doxycycline treatment, will be assessed in diverse healthcare settings.

In conclusion, the routine inclusion of testing for scrub typhus and other doxycycline-treatable causes, coupled with prioritising doxycycline in empirical treatment guidelines for AES in this region, has the potential to significantly improve patient outcomes and inform public health strategies. The well-performing clinical prediction models, translated into practical, user-friendly point-scoring systems, provide valuable tools to support this approach, pending external validation.

## Contributors

Conceptualisation: TD, BS, TS, VKG, VR, LT, RKD.

Data Curation: TD, MJ, UVK, AL, SM, NM, AVL, FSD, SVS, MK, VKG.

Formal Analysis: TD, MJ, UVK, BS, ST, CP, PPV, LT, RKD.

Funding Acquisition: TD, TS, RSM, VR, RY.

Investigation: TD, MJ, UVK, ST, AL, SM, NM, CP, AVl, FSD, SVS, MK, VKG, GVB.

Methodology: TD, MJ, BS, ST, PPV, AVL, FSD, SVS, MK, BDM, TS, VKG, GVB,VR, RY, LT, RKD.

Project administration: TD, MJ, ST, SM, NM.

Supervision: TS, RSM, BDM, VR, VR, LT, RKD.

Validation: TD, UVK, BS, MJ, ST.

Visualisation: TD, MJ, MS, ST, BDM, TS, RY, LT, RKD.

Writing- original draft: TD, MJ, UVK, BS, ST, PPV, LT, RKD.

Writing-reviewing & editing: BS, AVL, FSD, SVS, MK, BDM, RSM, TS, VKG, GVB, RV, RY, LT, RKD.

## Data sharing statement

De-identified participant data, the study protocol, and the statistical analysis plan will be made available to qualified researchers upon reasonable request to the corresponding author (TD; tinadamodar86@gmail.com). Applicants must submit a methodologically sound proposal outlining a valuable research question. Data will be provided once the proposal is approved, and a data-access agreement has been signed.

## Editor note

The Lancet Group takes a neutral position with respect to territorial claims in published maps and institutional affiliations.

## Declaration of interests

BS reports grant funding from the Medical Research Council ([MRC], grant MR/V033441/1) and UK National Institute for Health and Care Research ([NIHR], grant 17/63/110). CP was supported by the DBT/Wellcome Trust India Alliance Fellowship (IA/E/15/1/502336). BDM is supported to conduct COVID-19 neuroscience research by the UKRI/MRC (MR/V03605X/1) and by the NIHR Health Protection Research Unit (HPRU) in Emerging and Zoonotic Infections at University of Liverpool. BDM is also supported for additional neurological inflammation research due to viral infection by grants from: the NIHR, the MRC [MC_PC_19059] the MRC/UKRI (MR/V007181/1), MRC (MR/T028750/1), Wellcome (ISSF201902/3) and Medical Research Foundation (MRF) [MRF-CPP-R2-2022-100003]. TS reports grant funding from NIHR (IS-HPU-1112-10117, 17/63/110), MRC (MR/V033441/1); royalties from Oxford University Press, Elsevier, Liverpool University Press, and Cambridge University Press; consulting fees from GSK, Siemens, and the MHRA, and Valneva; support from The Pandemic Institute, Liverpool; and has a patent filed for a test for bacterial meningitis (GB1606537.7). LT is supported by the NIHR HPRU in Emerging and Zoonotic Infections (NIHR200907) at the University of Liverpool in partnership with Public Health England (PHE), in collaboration with Liverpool School of Tropical Medicine and the University of Oxford. LT has also received consulting fees from MHRA and from AstraZeneca and Synairgen, paid to the University of Liverpool; speakers’ fees from Eisai Ltd; and support for conference attendance from AstraZeneca. RY has received grants from Indian Council of Medical Research and Pratiksha Trust. RY has also received royalties from Jaypee Publishers (New Delhi). The other authors declare no conflicts of interest.

## Appendix ASupplementary data

Supplementary Information

Tripod-Checlist_TLRH
