# Supplementary material for: Development and internal validation of clinical prediction models for scrub typhus and doxycycline-treatable causes in paediatric acute encephalitis syndrome in Karnataka, India: a multicentre, prospective study
Source: Lancet Reg Health Southeast Asia. 2025 Jun 26;39:100626. doi: 10.1016/j.lansea.2025.100626 (PMC12266524; doi:10.1016/j.lansea.2025.100626)
Supplement: Supplementary Information [file mmc1.docx]

**SUPPLEMENTARY INFORMATION**

CONTENTS

1. Referral centre description…………………………………………………………………………………………………………………………... 1
2. Data collection………………………………………………………………………………………………………………………………………. 2
3. Microbiological testing methods and serological assays……………………………………………………………………………………………. 2
4. Table S1: Target oligonucleotide sequences and their details………………………………………………………………………………………..6
5. Table S2: Recruitment period and number of patients enrolled from each site …………………………………………………………………….. 9
6. Table S3: Diagnostic classification of multiple positive cases……………………………………………………………………………...………..9
7. Figure S1: State-wise distribution of cases………………………………………………………………………………………………………….. 10
8. Figure S2: District-wise distribution of cases……………………………………………………………………………………………………….. 11
9. Figure S3: Age-wise distribution of cases…………………………………………………………………………………………………………... 16
10. Figure S4: Year-wise distribution of aetiologies ……………………………………………………………………………………………………. 17
11. Figure S5: Monthly distribution of aetiologies……………………………………………………………………………………………………… 18
12. Clinical intervals…………………………………………………………………………………………………………………………………….. 18
13. Table S4: Demographic and clinical characteristics of the study population, according to respective causes of AES …………………………….. 19
14. Patient outcome………………………………………………………………………………………………………………………………….… 24
15. Table S5: Demographic, clinical characteristics and laboratory parameters of cases with favourable and unfavourable outcomes…………….… 25
16. Table S6: Distribution of aetiologies included in the development of model for scrub typhus diagnosis………………………………………..… 28
17. Table S7: Demographics and clinical characteristics of children with scrub typhus and other causes of AES………………………………...….. 29
18. Table S8: Distribution of aetiologies included in the development of model for diagnosis of doxycycline-treatable causes……………………… 34
19. Table S9: Demographics and clinical characteristics of children with and without doxycycline-treatable causes………………………………… 36
20. Figure S6: No. (%) of missing values in the dataset used for developing the model for scrub typhus diagnosis……………………………..…… 40
21. Figure S7: No. (%) of missing values in the dataset used for developing the model for doxycycline-treatable causes…………………………… 41
22. Table S10: Univariable odds ratios for risk of scrub typhus and doxycycline-treatable cause…………………………………………………….. 42
23. Details of additional models developed…………………………………………………………………………………………………………….. 43
24. Table S11: Multivariable presentation-at-hospital models, adjusted for shrinkage………………………………………………………………… 43
25. Table S12: Multivariable post-LP models, adjusted for shrinkage…………………………………………………………………………………. 44
26. Table S13: Scoring system for probability of outcome, using presentation-at-hospital models…………………………………………………… 45
27. Table S14: Scoring system for probability of outcome, using post-LP models……….…………………………………………………………... 46
28. Table S15: Estimate of risk based on scores of presentation-at-hospital models…………………………………………………………………. 47
29. Table S16: Estimate of risk based on scores of post-LP models…………………………………………………………………………………. 48
30. Table S17: Model performance………………………………………………………………………………………………………………….… 49
31. Figure S8: Calibration plots for the models…………………………………………………………………………………………………..…… 50
32. References……………………………………………………………………………………………………………………………………….… 52

**Referral centre description**

Indira Gandhi Institute of Child Health (IGICH), located in Bengaluru, Karnataka, was established in 1991 as an autonomous public health institution under the Rajiv Gandhi University of Health Sciences (RGUHS). Situated within the South Hospital Complex, IGICH is a 450-bed tertiary care referral centre providing comprehensive paediatric multispecialty care. IGICH offers a wide array of services, including general paediatric clinics, vaccination programmes, paediatric neurology, neonatal and paediatric critical care, rheumatology, endocrinology, haemato-oncology, nephrology, paediatric surgeries, and laboratory services. The institution is not only a hub for advanced patient care but also a centre for postgraduate paediatric medical training and research. As a referral centre, IGICH caters to patients from Karnataka as well as neighbouring states like Andhra Pradesh, Telangana, and Tamil Nadu, and occasionally from distant states such as West Bengal. Annually, its paediatric outpatient clinics and emergency services see approximately 25,000 to 30,000 patients, while the paediatric intensive care unit (PICU) admits 6,000 to 7,000 patients. Among these, the hospital handles an average of 20 to 25 cases of acute encephalitis syndrome per month, amounting to around 300 cases per year. However, during the COVID-19 pandemic in 2020 and 2021, IGICH was designated as a paediatric COVID care centre, which significantly impacted non-COVID admissions during those two years. The hospital provides basic diagnostic services and collaborates with institutions such as the National Institute of Virology (NIV) and NIMHANS for advanced microbiological and virological diagnostics. The in-house microbiology laboratory also supports routine diagnostic services, such as serological assays for viral (rapid tests) and rickettsial pathogens (Weil-Felix test) and bacterial culture.

St. John’s Medical College Hospital is in Bengaluru and was established in 1963. Adults and children are admitted to this hospital, which during the study period had a capacity of 1600 beds for adults, 100 beds for children in general paediatric wards and 15 beds in the PICUs. Each year, around 67,000 adults and 3600 children are hospitalised. Of these children, 1200 are admitted in intensive care. Children with acute and chronic neurological problems especially from Karnataka, Tamil Nadu, Andhra Pradesh (states) are referred to St. John’s Medical College Hospital. Patients included in the study were hospitalised in intensive care units or general paediatric wards. The microbiology Laboratory of the hospital facilitates laboratory diagnostics.

Vani Vilas hospital is one of the oldest maternity and children’s hospitals run by the government, attached to Bangalore Medical College and Research Institute, and inaugurated by the Maharaja of Mysore, Sri Krishna Rajendra Wodeyar in 1935. Women and children are admitted to this hospital, which during the study period had a capacity of 536 beds in general and 116 beds for children. Each year, more than thirty thousand patients are admitted to the hospital of which around 9000 are children and neonates. Of these children, around 3000 children are admitted in the PICU or paediatric wards. The paediatric neurology unit is also located here and this is the one of the referral paediatric neurology units in Bengaluru, therefore, children with acute and chronic neurological problems especially from Karnataka, Tamil Nadu, Andhra Pradesh, West Bengal are referred to this hospital. The viral diagnostic and research laboratory (VRDL) partners with this hospital to provide diagnostic support. In the 2020 and 2021 COVID outbreak, paediatric admissions due to non-COVID causes were reduced as the hospital was a designated centre for COVID-related admissions.

**Data collection**

The comprehensive case report included: (i) socio-demographic information; (ii) clinical findings, including neurological examination at admission (iii) Baseline laboratory data (iv) Liverpool outcome score (LOS) performed three months after discharge. An on-site research office was in-charge of the case report form collection with the physician in charge of the patient and was also in charge of the data completion and data entry. Data were entered online using an online platform (<https://www.clappia.com>). Patient’s names were already anonymised before data entry. The data was audited against discharge summaries by the principal investigator later.

**Microbiological testing**

Briefly, the first line testes included serological assays for IgM antibodies detection to following pathogens: Japanese encephalitis virus (JEV) (JEV IgM Capture ELISA; National Institute of Virology, Pune, India), *O. tsutsugamushi* (Scrub Typhus Detect IgM ELISA; InBios, Seattle, WA, USA), dengue virus (Panbio Dengue IgM Capture ELISA, Brisbane, Australia), chikungunya virus (CHIKjj Detect IgM; InBios, Seattle, WA, USA), and *Leptospira* (Panbio Leptospira IgM ELISA, Brisbane, Australia), and rapid diagnostic test for malarial antigen (Bioline Malaria Ag P.f/P.v test). For children with illness duration of one week or less, serum samples were also tested for Dengue NS1 antigen using ELISA (Dengue NS1 antigen MICROLISA, J. Mitra and Co. Pvt. Ltd. India). Second line tests included multiplex real-time PCR for viral pathogens- HSV-1 & 2, varicella zoster virus (VZV), mumps virus, enterovirus, parechovirus (Fast-track Diagnostics, Luxembourg); and in-house real-time PCR for *Streptococcus pneumoniae, Neisseria meningitidis* and *Haemophilus influenzae*. Third line tests included IgM ELISA for West Nile virus (West Nile Detect IgM Capture ELISA; InBios, Seattle, WA, USA) and measles virus (Measles IgM ELISA, Calbiotech, USA).

Additionally, CSF samples of patients with IgM-positive ELISA in serum were diluted in 1:10 proportion for detection of IgM bodies against respective pathogens by ELISA. We also performed confirmatory tests on IgM-positive patients, including real-time PCR for *O. tsutsugamushi*, chikungunya virus, JEV, and dengue virus (1–4) on CSF and blood samples, and dengue NS1 antigen test (Dengue NS1 antigen MICROLISA, J. Mitra and Co. Pvt. Ltd. India). All samples were stored at -80 degree celsius. Stored samples from patients with no diagnosis using the laboratory algorithm were tested for *Mycobacterium*

*tuberculosis* (CSF), *O. tsutsugamushi* (Blood & CSF) and chikungunya virus (Blood & CSF) using real-time PCR. Additionally, CSF of patients with no diagnosis were also subjected to IgM ELISA for DENV, CHIKV, OT and Leptospira. Based on the results from 2020 to 2022, the algorithm was revised in April 2022 to incorporate simultaneous testing for *O. tsutsugamushi* and chikungunya (in both blood and cerebrospinal fluid) using real-time PCR as second-line tests.

**Serological assays**

Malarial parasites- Whole blood was tested for *Plasmodium vivax* and *P. falciparum* antigens with Malaria Ag P.f/Pan cassettes (SD BIOLINE). All rapid diagnostic tests followed manufacturers’ protocols and result interpretation

JEV: IgM antibodies in serum and CSF samples were detected using the National Institute of Virology (NIV) kit, as recommended by National Vector Borne Disease Control Programme (NVBDCP) using the manufacturer’s instructions.

Dengue virus: IgM antibodies in serum and CSF samples were detected using a commercial capture IgM ELISA kit (Panbio. Australia) approved by the European union for in vitro diagnostic use. Dengue NS1 antigen was detected by ELISA in serum using a commercial kit (J Mitra, India) according to manufacturer’s instructions. For real-time serotype PCR, CSF/ blood samples were subjected to extraction using the QIAamp Viral RNA Mini Kit (QIAGEN, Germany) and subsequently subjected to the CDC DENV-1–4 Real-Time RT-PCR Assay following the manufacturer's instructions.

*O. tsutsugamushi*: IgM antibodies in serum and CSF samples were detected using a commercial indirect IgM ELISA kit (Inbios, USA). Any serum and CSF sample that had an OD value equal to or above 0·800 and 0·500, respectively, were considered positive for scrub typhus based on recent studies in southern India. For PCR, DNA was extracted from CSF/ blood samples using the QIAamp DNA mini kit (QIAGEN, Germany) and real-time PCR targeting the 47kDa protein gene.

West Nile virus: IgM antibodies in serum samples were detected using an IgM capture ELISA kit (Inbios, USA). All samples that had a ratio of >0·400 were considered positive.

Chikungunya virus: IgM antibodies in serum and CSF samples were detected using a commercial capture IgM ELISA kit (Inbios, USA) approved by the European Union for in vitro diagnostic use. For real-time serotype PCR, CSF/ blood samples were subjected to extraction using the QIAamp Viral RNA Mini Kit (QIAGEN, Germany) and TaqMan real-time RT-PCR targeting the non-structural protein 4 region of CHIKV.

*Leptospira* sp: IgM antibodies in serum samples were detected using a commercial kit (Panbio, Australia) approved by the European union for in vitro diagnostic use. An AES case was considered as leptospira if IgM antibodies were positive in the serum.

Bacterial meningitis pathogens: Detection of *S. pneumoniae*, *H. influenzae* and *N. meningitidis*, DNA was carried out on CSF samples using a real time PCR (RT PCR) assay. DNA was extracted from CSF samples using QIAamp DNA mini kit (QIAGEN, Germany) and subjected to individual real time PCR for the three bacterial pathogens using in-house specific primers and probes. A sample was considered positive for bacterial DNA if the Ct values obtained in the CSF sample was <35 for the respective pathogen.

Herpes simplex virus: For confirming diagnosis of HSV-1 infection, detection of HSV DNA was carried out on CSF samples using a real time PCR assay. A sample was considered positive for HSV DNA if the Ct values obtained in the CSF sample was <38.

Enterovirus (EV): For confirming diagnosis of EV infection, detection of EV RNA was carried out in CSF samples using a real time RT-PCR (RT PCR) pan-enteroviral assay (genus specific). A sample was considered positive for EV RNA if the Ct value obtained in the CSF sample was <38.

*Mycobacterium tuberculosis* (MTb): For the diagnosis of tuberculosis, detection of MTb DNA was carried out on CSF samples using a real time PCR assay. A sample was considered positive for MTb DNA if the Ct values obtained in the CSF sample was <38.

Varicella Zoster virus (VZV): The RealStar® VZV PCR Kit 1·0 was used to detect viral nucleic acid for confirming VZV infection. Briefly, 10 µL of nucleic acid and 1 µL of internal control were added to each multiplex master mix for a final volume of 21 µL. Negative and positive controls were supplied with the kit and tested in each run. Results were interpreted according to the manufacturer's instructions

FTD Viral meningitis (Fast-track Diagnostics, Luxembourg, FTD-13) PCRs were used to detect – viral meningitis pathogens in a multiplex format. Briefly, 10 µL of nucleic acid was added to each multiplex master mix for a final volume of 25µL. Negative controls were extracted in tandem with sample extractions and the supplied positive controls were tested in each run. Results were interpreted according to the manufacturer's instructions.

**Table S1: Target oligonucleotide sequences and their details**

| **Virus Target** | **Direction** | **Primer/Probe name** | **Sequence (5'-3')** | **Labels** | **Reference** |
| --- | --- | --- | --- | --- | --- |
| **JEV*** | Sense | JEVF | GGCTCTTATCACGTTCTTCAAGTTT |  |  |
|  | Reverse | JEVR | ACTAGTAAGATGTTTCATTGCCACACTCT |  | (1) |
|  | Probe | JEV Probe | ATTAGCCCCGACCAAGGCGCTTT | 6FAM/BHQ1 |  |
| **DENV1** | Sense | DEN-1 F8973 | CAAAAGGAAGTCGTGCAATA |  |  |
|  | Reverse | DEN-1 C9084 | CTGAGTGAATTCTCTCTACTGAACC |  | (2) |
|  | Probe | DEN-1 PRB8998 | CATGTGGTTGGGAGCACGC | 6FAM/BHQ1 |  |
| **DENV2** | Sense | DEN-2 F1506 | CAGGTTATGGCACTGTCACGAT |  | (2) |
|  | Reverse | DEN-2 C1583 | CCATCTGCAGCAACACCATCTC |  |  |
|  | Probe | DEN-2 PRB1534 | CTCTCCGAGAACAGGCCTCGACTTCAA | Hex/BHQ1 |  |
| **DENV3** | Sense | DEN-3 F740 | GGACTGGACACACGCACTCA |  | (2) |
|  | Reverse | DEN-3 C813 | CATGTCTCTACCTTCTCGACTTGTCT |  |  |
|  | Probe | DEN-3 PRB762TR | ACCTGGATGTCGGCTGAAGGAGCTTG | TexasRed/BHQ2 | |
| **DENV4** | Sense | DEN-4 F904 | TTGTCCTAATGATGCTGGTCG |  |  |
|  | Reverse | DEN-4C992 | TCCACCTGAGACTCCTTCCA |  | (2) |
|  | Probe | DEN-4 PRB960 | TTCCTACTCCTACGCATCGCATTCCG | Cy5/BHQ3 |  |
| **Chikungunya** | Sense | CHIV6856F | TCACTCCCTGTTGGACTTGATAGA |  |  |
|  | Reverse | CHIV6981R | TTGACGAACAGAGTTAGGAACATACC |  | (3) |
|  | Probe | CHIV6919Pr | AGGTACGCGCTTCAAGTTCGGCG | 6FAM/BHQ1 |  |
| **Enterovirus** | Sense | SCDCV-F | CCCTGAATGCGGCTAATCC |  |  |
|  | Reverse | SCDCV-R | ATTGTCACCATAAGCAGCCA |  | (4) |
|  | Probe | SCDCV-P | AACCGACTACTTTGGGTGTCCGTGTTTC | 6FAM/TAMRA |  |
| **HSV-1** | Sense | HSV-1F | TTCTCGTTCCTCACTGCCTCCC |  |  |
|  | Reverse | HSV-1R | GCAGGCACACGTAACGCACGCT |  | (5) |
|  | Probe | HSV-1P | CGTCTGGACCAACCGCCACACAGGT | 6FAM/BHQ1 |  |
|  |  |  |  |  |  |
| **Bacteria Target** | **Direction** | **Primer/Probe name** | **Sequence (5'-3')** | **Labels** | **Reference** |
| ***Streptococcus pneumoniae*** | Sense | S.Pneumo-Forward | GCCCTAATAAATTGGAGGATCTAATGA |  | (6) |
|  | Reverse | S.Pneumo-Reverse | GACCAGAAGTTGTATCTTTTTTTCCG |  |  |
|  | Probe | S.Pneumo-Probe-FAM | CTAGCACATGCTACAAGAATGATTGCAGAAAGAAA | 6FAM/BHQ1 |  |
| ***Haemophilus influenzae*** | Sense | Hpd3H.InfhpdF7 | AGATTGGAAAGAAACACAAGAAAAAGA |  | (7) |
|  | Reverse | Hpd3H.InfhpdR | CACCATCGGCATATTTAACCACT |  |  |
|  | Probe | Hpd3 | AAACATCCAATCGTAATTATAGTTTACCCAATAACCC | 6FAM/BHQ1 |  |
| ***Neisseria meningitidis*** | Sense | N.men-Forward | GCTGCGGTAGGTGGTTCAA |  |  |
|  | Reverse | N.men-Reverse | TTGTCGCGGATTTGCAACTA |  | (8) |
|  | Probe | N.men-Probe-FAM | CATTGCCACGTGTCAGCTGCACAT | 6FAM/BHQ1 |  |
| ***Mycobacterium tuberculosis*** | Sense | M.Tb-Forward | GGGTAGCAGACCTCACCTATGTG |  | (9) |
|  | Reverse | M.Tb-Reverse | TAGGCGTCGGTGACAAAGG |  |  |
|  | Probe | M.Tb-Probe-FAM | TCGCCTACGTGGCCTTT | 6FAM/BHQ1 |  |
| ***Orientia tsutsugamushi*** | Sense | Ori-F | AACTGATTTTATTCAAACTAATGCTGCT |  | (10) |
|  | Reverse | Ori-R | TATGCCTGAGTAAGATACRTGAATRGAATT |  |  |
|  | Probe | Ori-Pr | TGGGTAGCTTTGGTGACCGATGTTTAATCT | 6FAM/BHQ1 |  |
|  |  |  |  |  |  |

*The PCR primers for JEV Taqman assay were provided by Dr. Barbara W. Johnson, Diagnostic & Reference Laboratory, Arbovirus Diseases Branch, Division of Vector-Borne Infectious Diseases, centres for Disease Control and Prevention (CDC), Fort Collins, Colorado, USA. Primers and fluorogenic probes were designed by using the PrimerExpress version 2.0.0 (PE Applied Biosystems, Foster City, CA) based on the JEV SA14 complete genome sequence (Genbank accession number M55506).

**Table S2: Recruitment period and number of patients enrolled from each site (N=714)**

| **Hospital** | **Recruitment period** | **No. recruited (%)** |
| --- | --- | --- |
| IGICH | Aug 27, 2020–Feb 28, 2023 | 476 (66) |
| SJMC | Feb 14, 2020–Feb 28, 2023 | 174 (24) |
| BMC | July 18, 2021–Feb 28, 2023 | 64 (9) |

**Table S3: Diagnostic classification of multiple positive cases (n=42)**

| **Distribution of microbiological diagnosis in cases with multiple aetiologies** | **No. patients** | **%** |
| --- | --- | --- |
| Possible JEV, Possible Dengue virus | 9 | 21• 4 |
| Possible JEV, Possible *Orientia tsutsugamushi* | 7 | 16·7 |
| Possible *Orientia tsutsugamushi*, Possible *Leptospira sp*· | 3 | 7·1 |
| Probable JEV, Probable *Orientia tsutsugamushi* | 2 | 4·8 |
| Probable Chikungunya virus, Probable *Orientia tsutsugamushi* | 2 | 4·8 |
| Possible JEV, Possible *Leptospira sp·* | 2 | 4·8 |
| Possible Dengue virus, Possible WNV | 2 | 4·8 |
| Possible Dengue virus, Possible *Orientia tsutsugamushi* | 2 | 4·8 |
| Possible Dengue virus, Possible *Leptospira sp·* | 2 | 4·8 |
| Possible Chikungunya virus, Possible Dengue virus | 2 | 4·8 |
| Probable *Orientia tsutsugamushi*, Probable *Leptospira sp·* | 1 | 2·4 |
| Probable *Orientia tsutsugamushi*, Probable Chikungunya virus | 1 | 2·4 |
| Probable JEV, Probable WNV | 1 | 2·4 |
| Probable JEV, Probable Dengue virus, Probable *Orientia tsutsugamushi* | 1 | 2·4 |
| Probable Dengue virus, Probable *Orientia tsutsugamushi*,Possible JEV | 1 | 2·4 |
| Probable Dengue virus, Probable *Orientia tsutsugamushi*, Possible JEV | 1 | 2·4 |
| Probable Chikungunya virus, Probable *Orientia tsutsugamushi*, Possible *Leptospira sp·* | 1 | 2·4 |
| Possible JEV, Possible Dengue virus, Possible *Orientia tsutsugamushi* | 1 | 2·4 |
| Possible JEV, Possible Dengue virus, Possible *Leptospira sp*· | 1 | 2·4 |
| Total | 42 | 100 |

**Figure S1: State wise distribution of cases (n=561)**


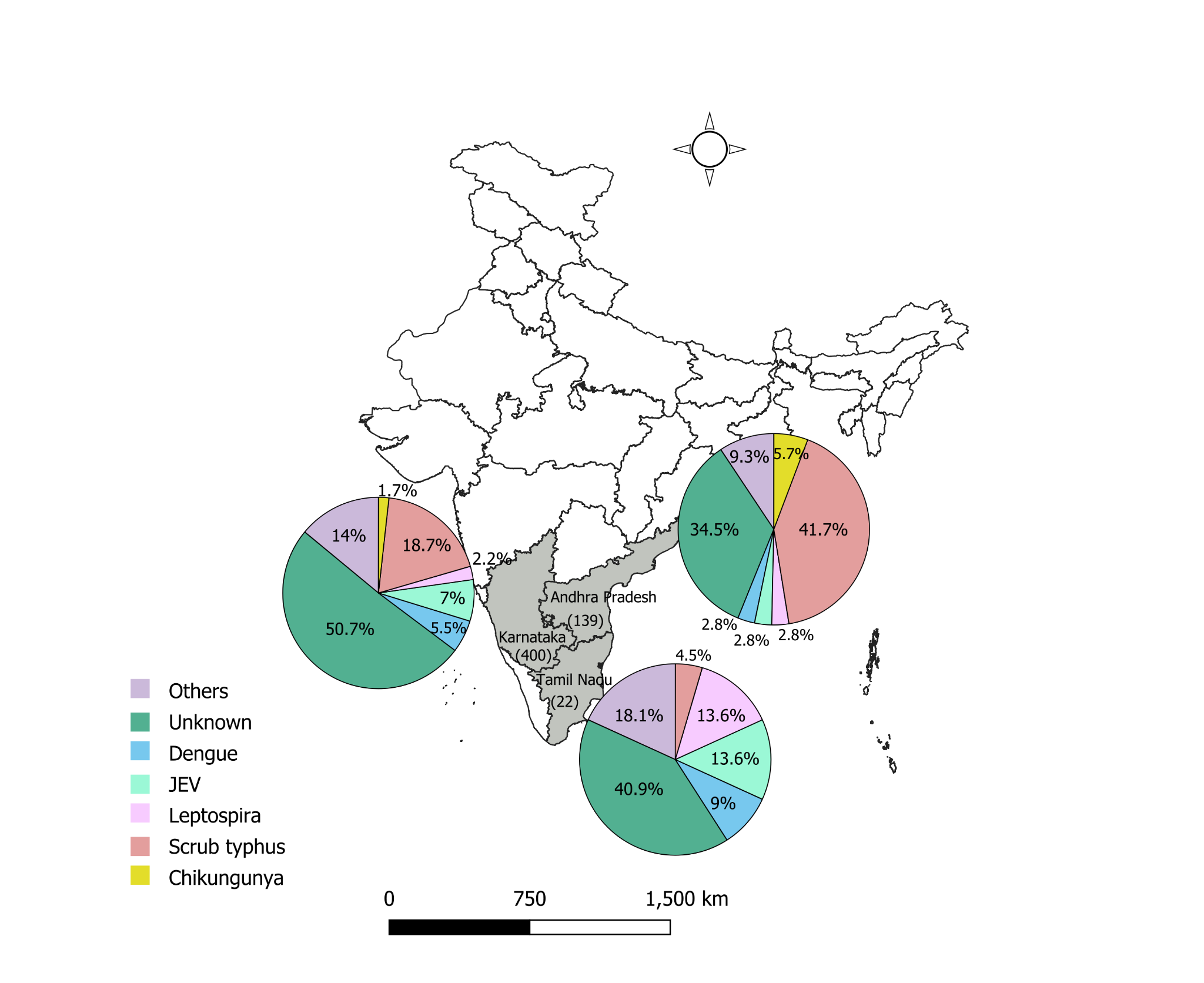


*Of the 570 children with available information on their resident state, 399 (70%) were from Karnataka, 139 (24%) from Andhra Pradesh, 23 (4%) from Tamil Nadu, and 9 (1·6%) from northern and eastern states such as Uttar Pradesh, Bihar, and West Bengal. Not shown in the figure- cases from states other than Karnataka, Andhra Pradesh and Tamil Nadu

**Figure S2: District-wise distribution of AES cases**

| 1. All aetiologies | 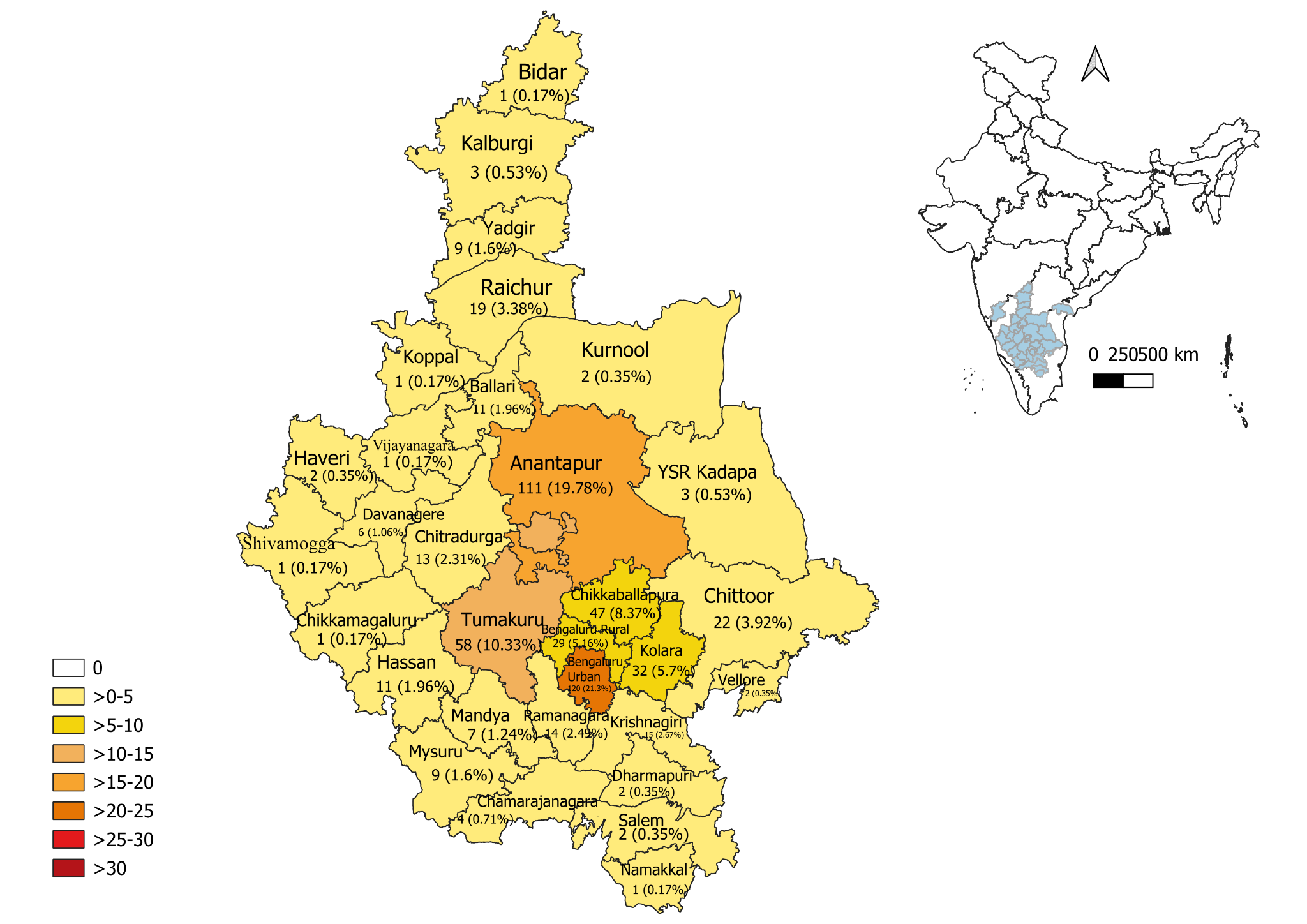 |
| --- | --- |
| B. Scrub typhus | 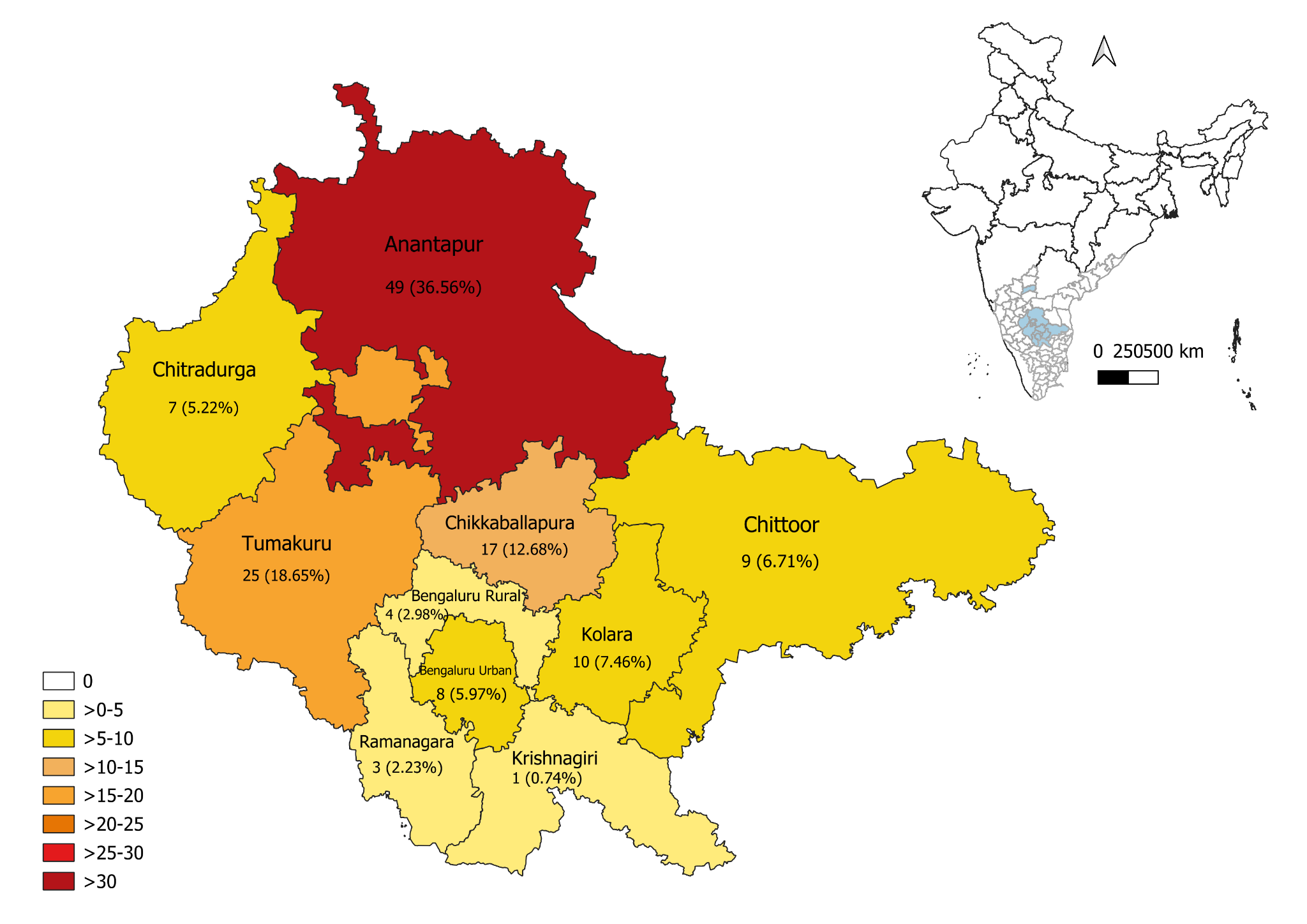 |
| C. JEV | 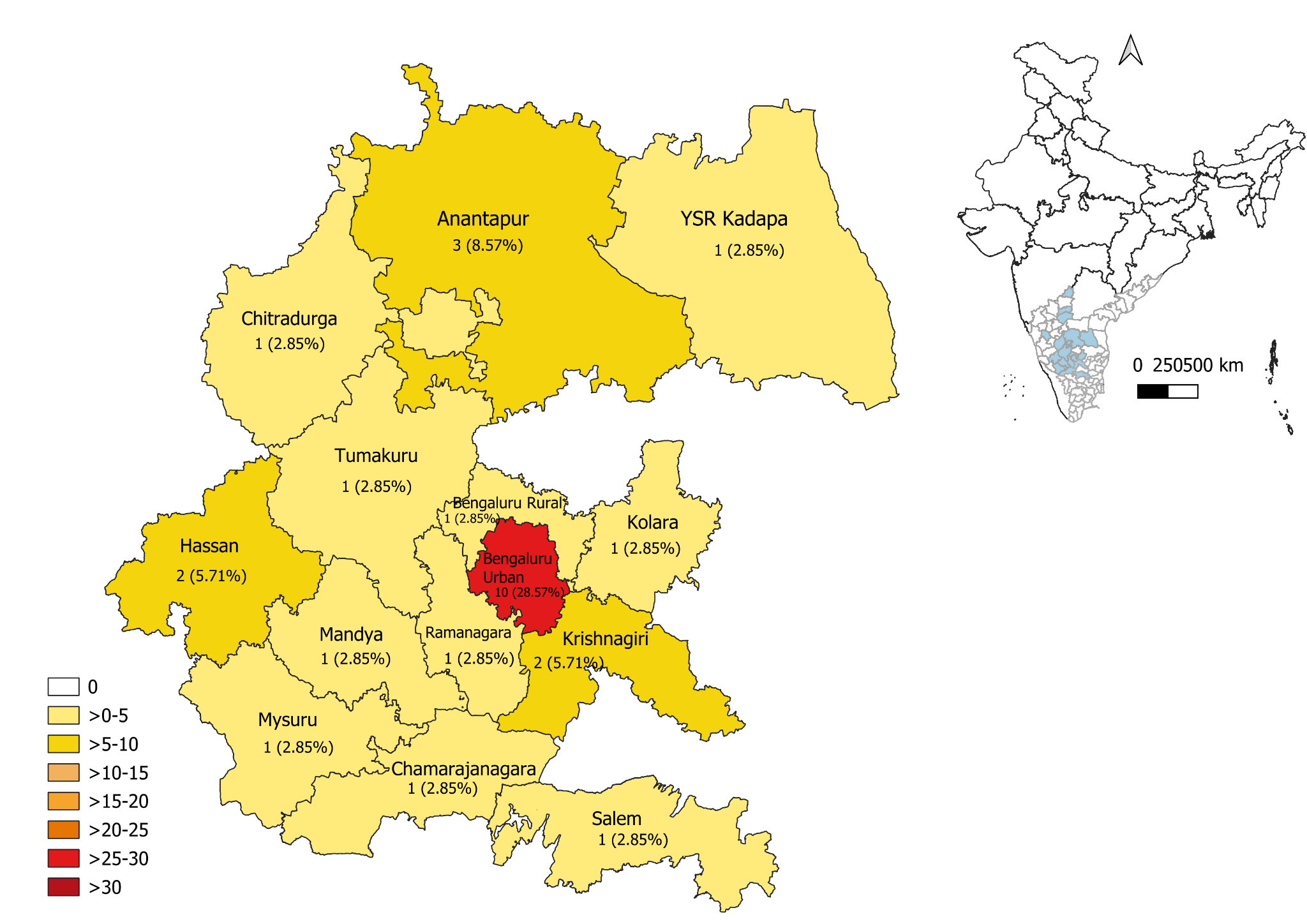 |
| D. Dengue | 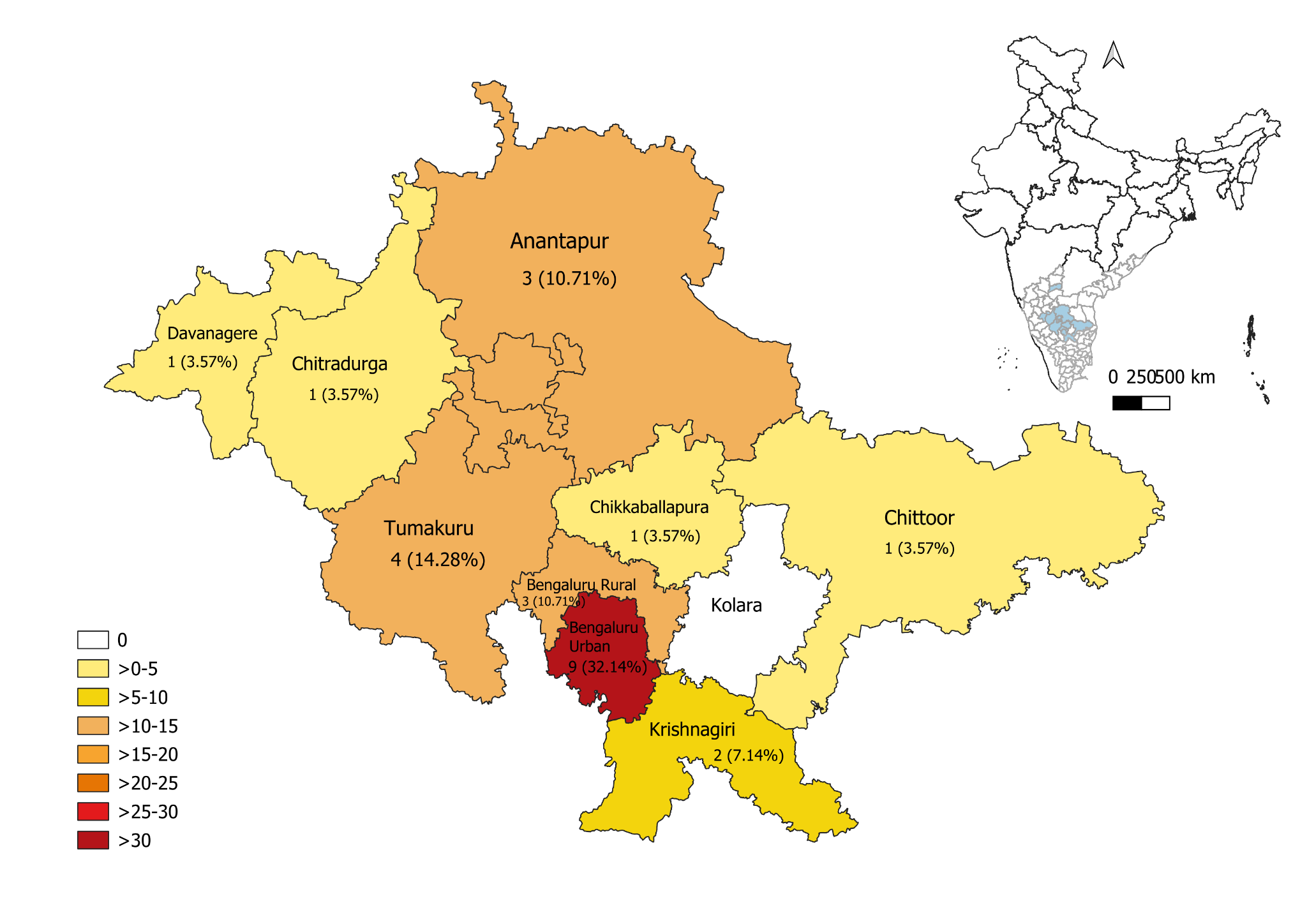 |
| E. Unknown | 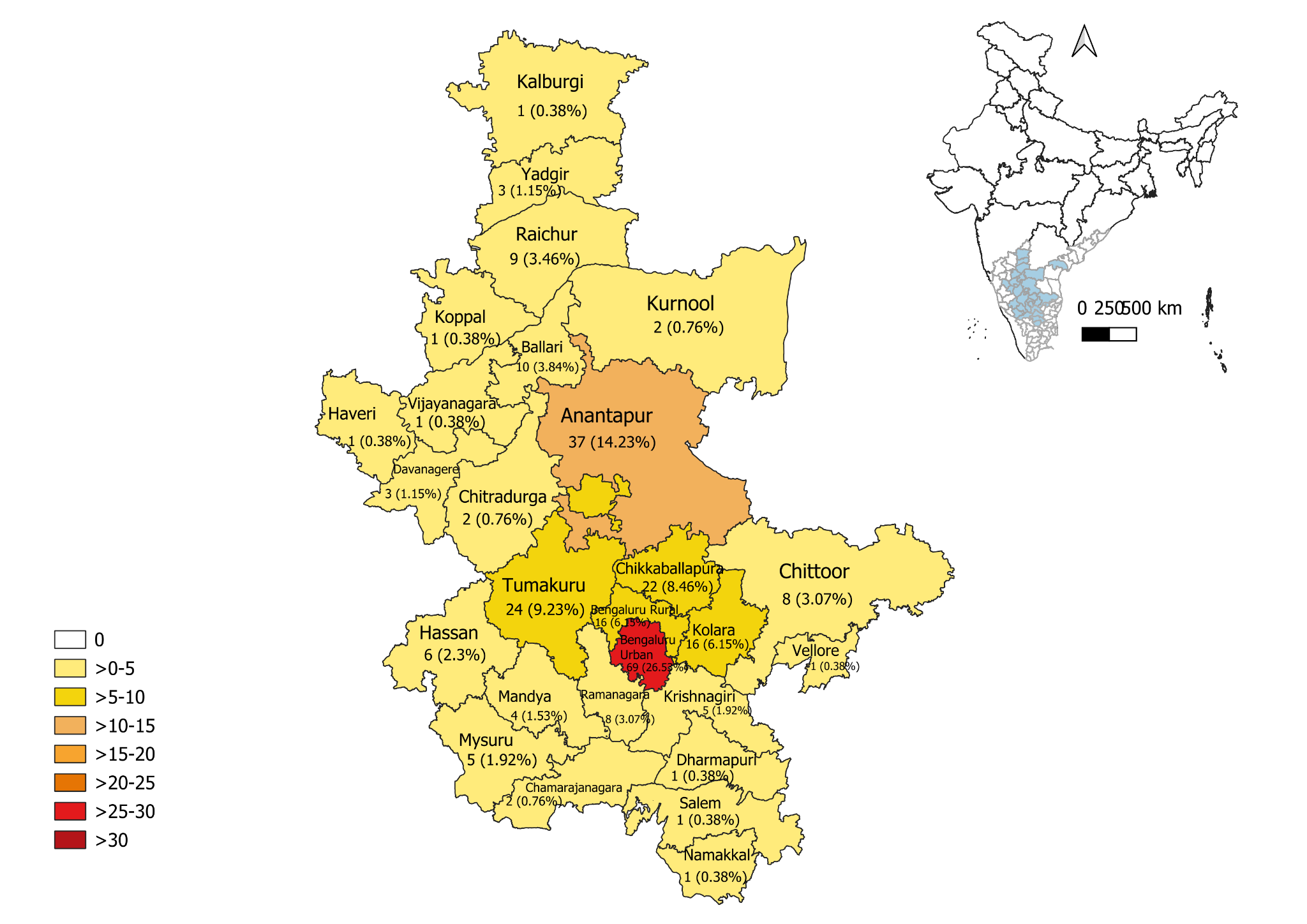 |

Percentage-based gradient map showing district-wise distribution of cases due to

A. all aetiologies [Not shown- additional two cases from Karnataka district Belagavi (1) and Andhra Pradesh district Guntur (1)],

B. Scrub typhus [Not shown- additional one case from Karnataka district Yadgir],

C. JEV [Not shown- additional eight cases from Karnataka districts Raichur (4), Yadgir (2), Haveri (1), Bidar (1)],

D. Dengue [Not shown- additional three cases from Karnataka districts Yadgir (3)],

E. Unknown/ Unidentifiable infectious aetiologies [Not shown- one case from Andhra Pradesh district Guntur (1)]

**Figure S3: Age-wise distribution of all aetiologies (n=587)**

**
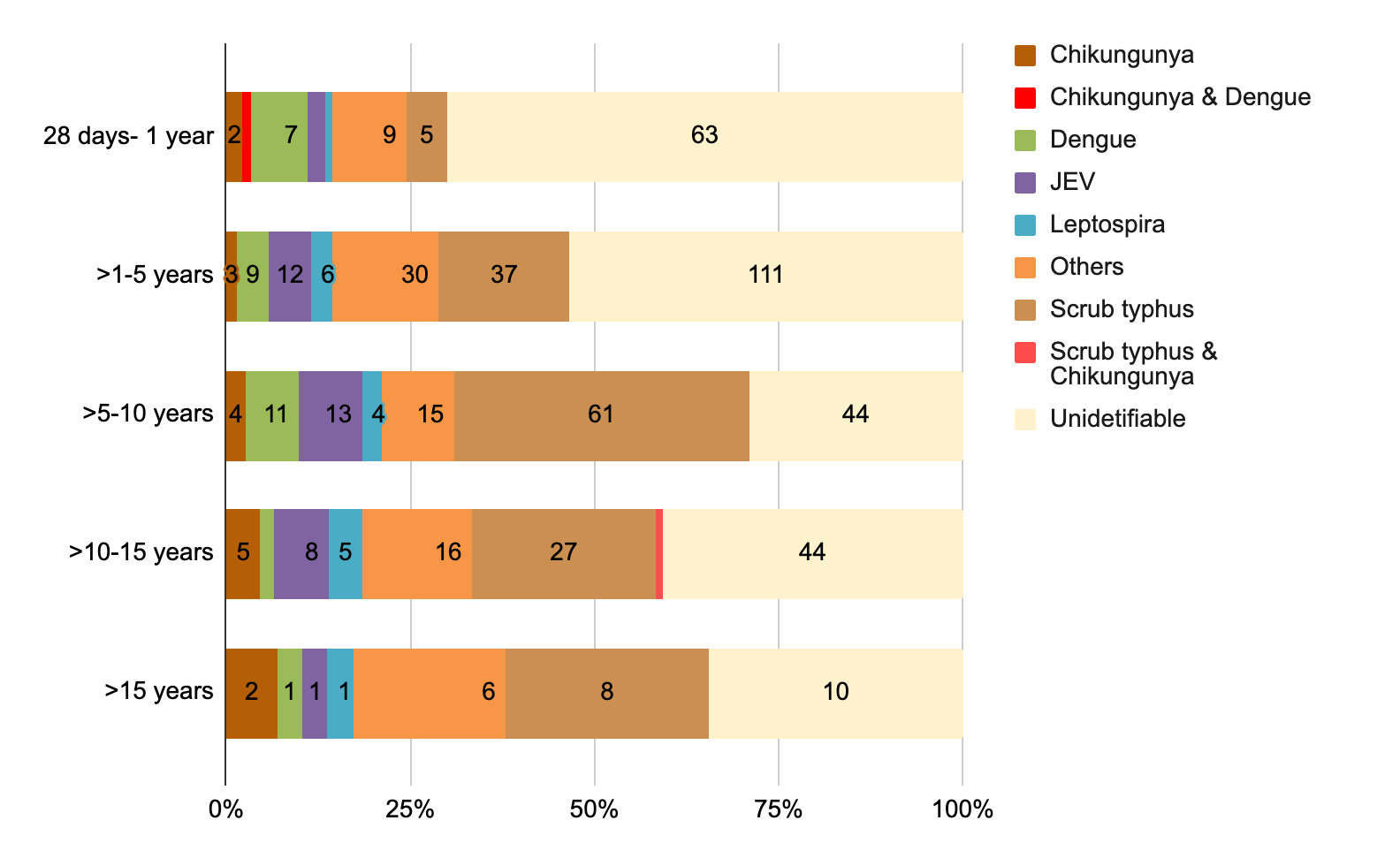
**

**Figure S4: Year-wise distribution of aetiologies (n=587)**
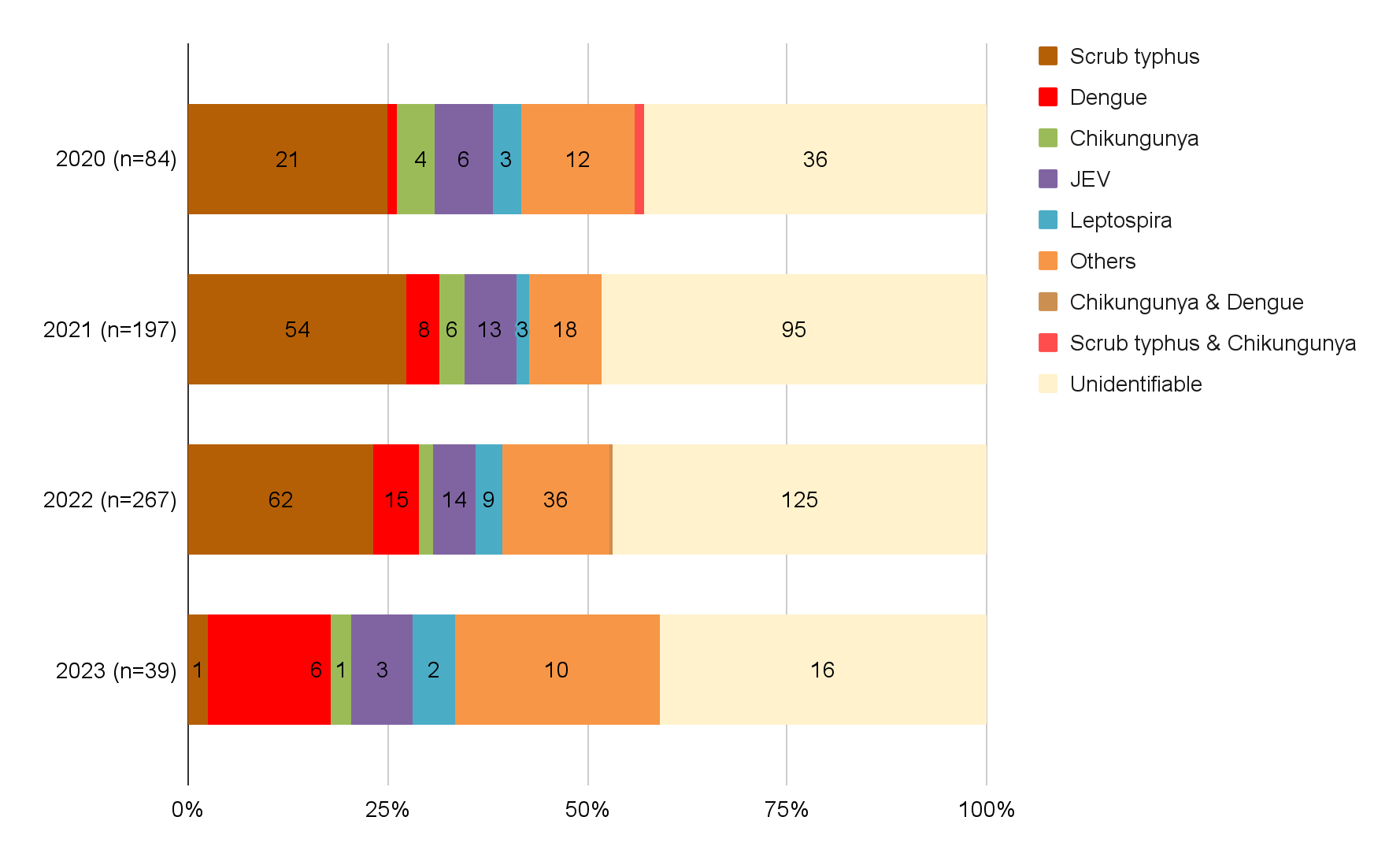


**Figure S5: Monthly distribution of aetiologies (n=587)**

| 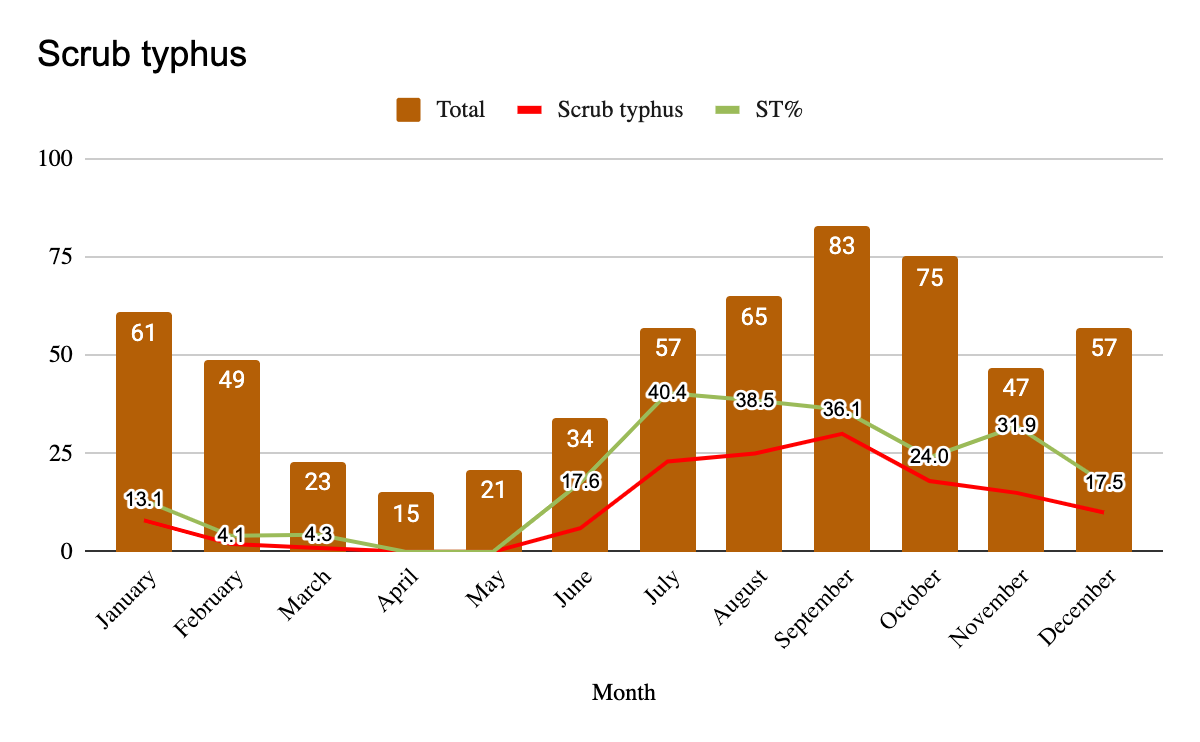 | 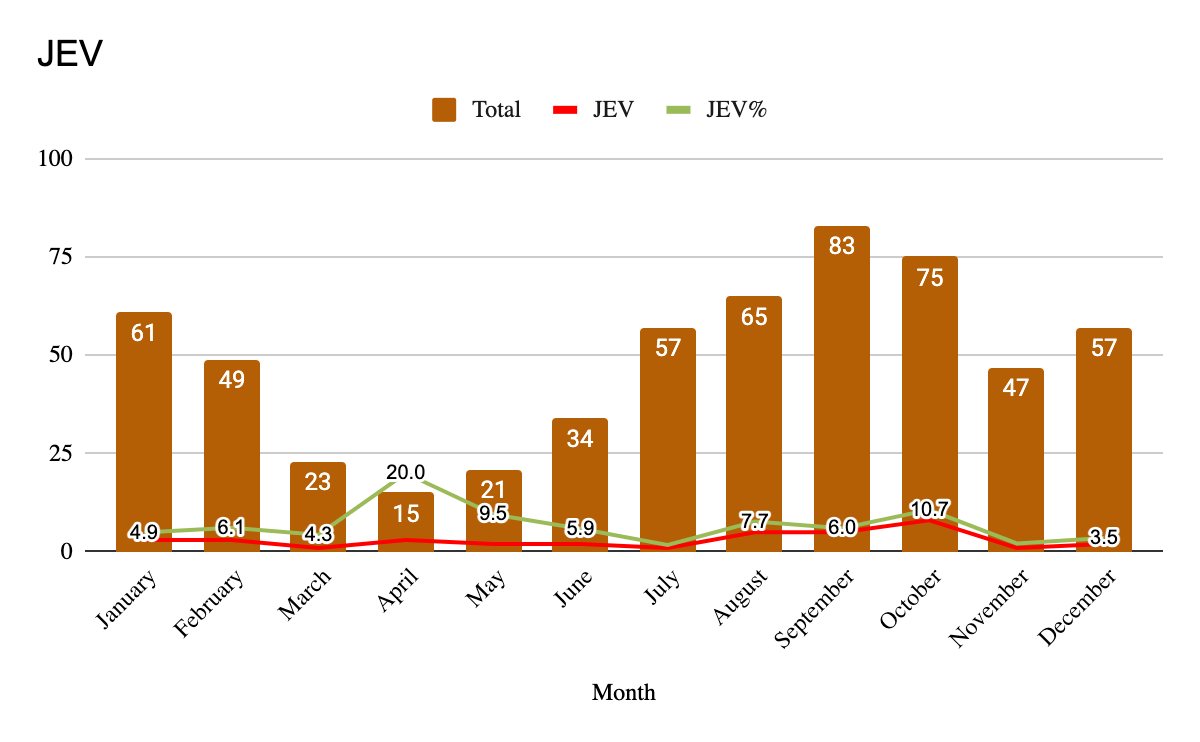 | 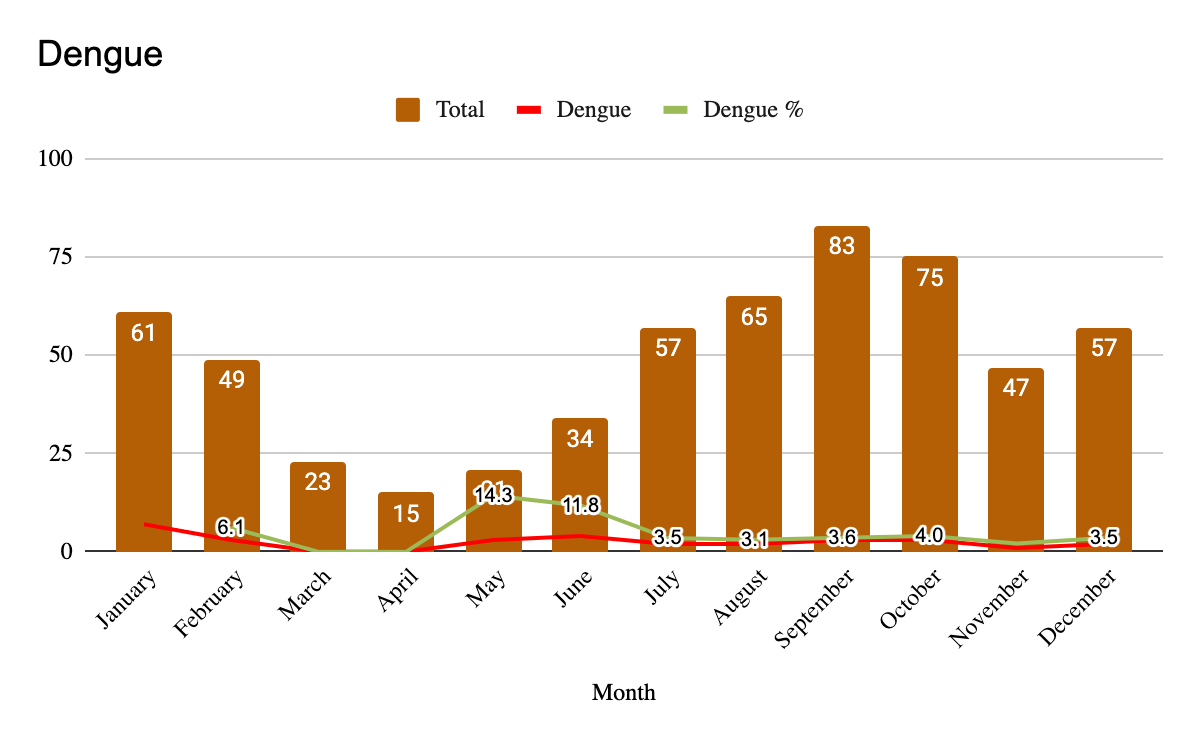 |
| --- | --- | --- |
| 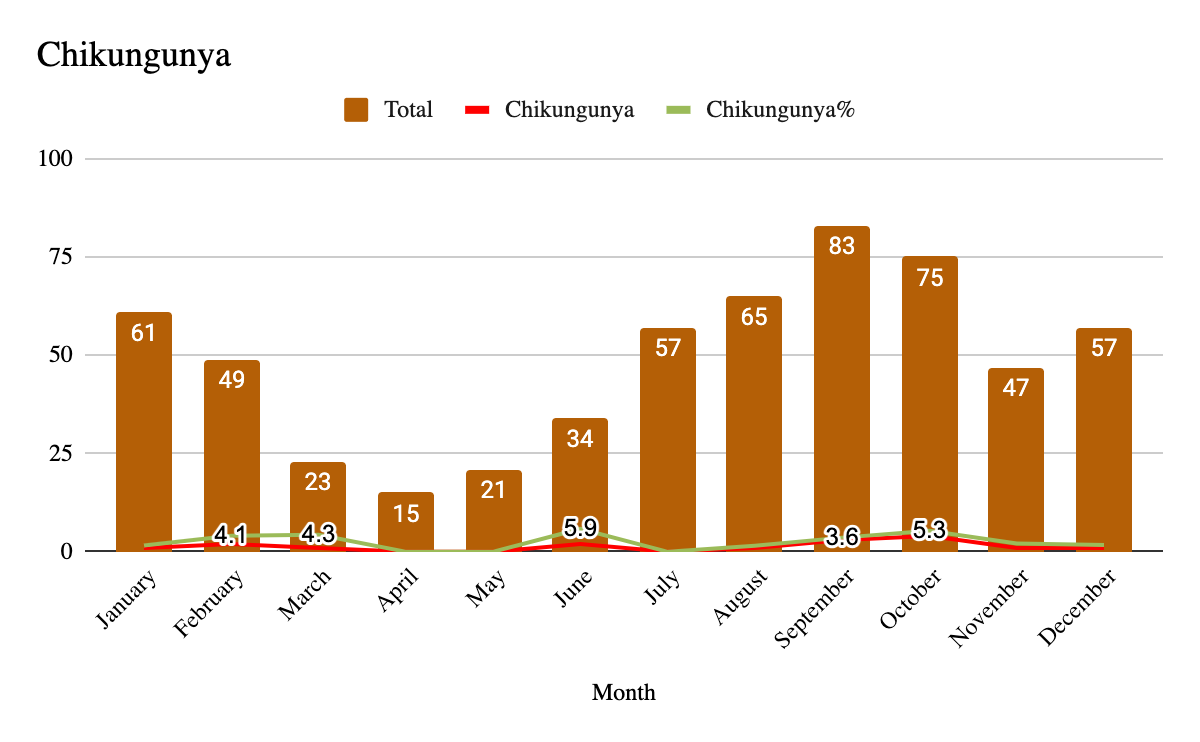 | 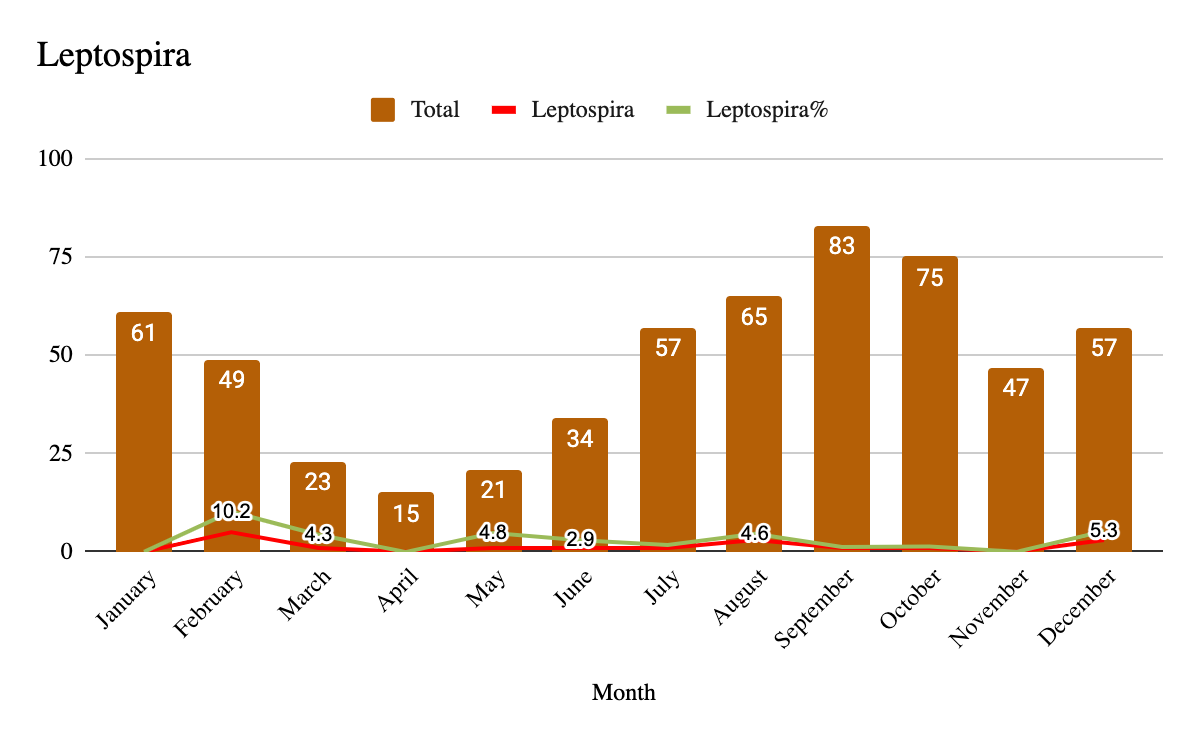 | 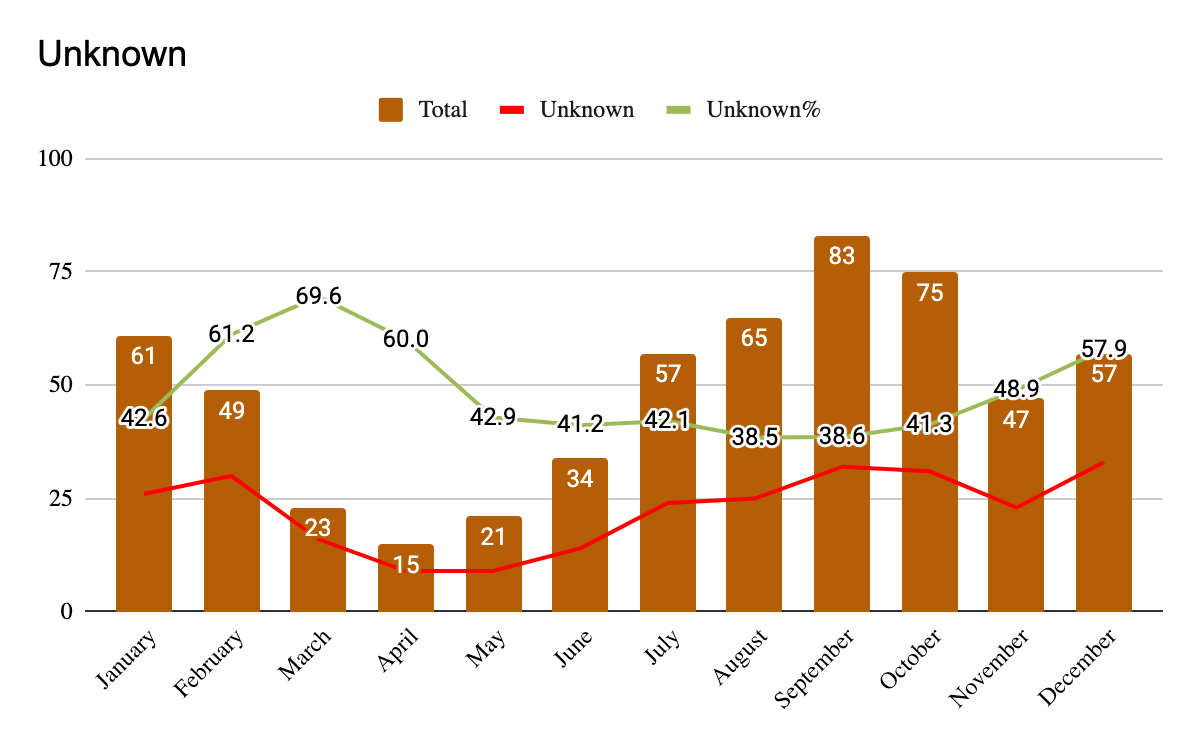 |

**Clinical intervals**

The median interval from symptom onset to hospital admission was 5 days (IQR 3–7), from symptom onset to lumbar puncture was 7 days (IQR 4–11), and from symptom onset to the submission of samples for microbiological testing was 9 days (IQR 6–13). The median hospitalisation duration was 9 days (IQR 7–15).

**Table S4- Demographic and clinical characteristics of the study population, according to respective causes of AES (n=587)**

| **Baseline Clinical/ Laboratory Variables** | **No patients (%)** |  | **ST** | **DEN** | **CHIK** | **JEV** | **Leptospira** | **Others** | **Unknown** | **Total** |
| --- | --- | --- | --- | --- | --- | --- | --- | --- | --- | --- |
| Total N (%) |  |  | 138 (23·5) | 30 (5·1) | 16 (2·7) | 36 (6·1) | 17 (2·9) | 78 (13·3) | 272 (46·3) | 587 |
| Age (years) | 587 (100·0) | Median (IQR) | 8·0 (4·2–10·8) | 4·5 (1·2–8·0) | 9·0 (4·2–14·2) | 7·0 (4·0–10·2) | 8·0 (3·0–12·0) | 5·0 (2·1–11·0) | 3·0 (1·2–8·0) | 5·0 (2·0–10·0) |
| Age group | 587 (100·0) | 28 days-1year | 4 (2·9) | 7 (23·3) | 1 (6·2) | 0 (0·0) | 1 (5·9) | 7 (9·0) | 44 (16·2) | 64 (10·9) |
|  |  | 1-5 years | 31 (22·5) | 8 (26·7) | 3 (18·8) | 12 (33·3) | 6 (35·3) | 29 (37·2) | 115 (42·3) | 204 (34·8) |
|  |  | 5-10 years | 53 (38·4) | 12 (40·0) | 4 (25·0) | 14 (38·9) | 3 (17·6) | 16 (20·5) | 53 (19·5) | 155 (26·4) |
|  |  | 10-15 year | 42 (30·4) | 2 (6·7) | 6 (37·5) | 9 (25·0) | 6 (35·3) | 20 (25·6) | 50 (18·4) | 135 (23·0) |
|  |  | >15 years | 8 (5·8) | 1 (3·3) | 2 (12·5) | 1 (2·8) | 1 (5·9) | 6 (7·7) | 10 (3·7) | 29 (4·9) |
| Gender | 587 (100·0) | Male | 76 (55·1) | 18 (60·0) | 12 (75·0) | 23 (63·9) | 10 (58·8) | 46 (59·0) | 157 (57·7) | 342 (58·3) |
|  |  | Female | 62 (44·9) | 12 (40·0) | 4 (25·0) | 13 (36·1) | 7 (41·2) | 32 (41·0) | 115 (42·3) | 245 (41·7) |
| Duration of Illness (days) | 587 (100·0) | Median (IQR) | 6·0 (4·0–7·0) | 4·5 (3·0–7·0) | 4·0 (3·0–5·5) | 4·5 (2·8–7·2) | 5·0 (3·0–5·0) | 5·0 (2·0–8·0) | 4·0 (2·0–7·0) | 5·0 (3·0–7·0) |
| Duration of Illness (>/ =5 days) | 587 (100·0) | No | 74 (53·6) | 12 (40·0) | 4 (25·0) | 16 (44·4) | 4 (23·5) | 36 (46·2) | 98 (36·0) | 244 (41·6) |
|  |  | Yes | 64 (46·4) | 18 (60·0) | 12 (75·0) | 20 (55·6) | 13 (76·5) | 42 (53·8) | 174 (64·0) | 343 (58·4) |
| Glasgow Coma Scale (GCS) score at admission | 490 (83·5) | 15 | 47 (34·1) | 7 (23·3) | 4 (25·0) | 4 (11·1) | 2 (11·8) | 23 (29·5) | 56 (20·6) | 143 (24·4) |
|  |  | >8-14 | 54 (39·1) | 5 (16·7) | 8 (50·0) | 22 (61·1) | 7 (41·2) | 29 (37·2) | 102 (37·5) | 227 (38·7) |
|  |  | </=8 | 22 (15·9) | 8 (26·7) | 3 (18·8) | 5 (13·9) | 6 (35·3) | 9 (11·5) | 67 (24·6) | 120 (20·4) |
| Fever as first presentation | 587 (100·0) | No | 5 (3·6) | 6 (20·0) | 1 (6·2) | 8 (22·2) | 3 (17·6) | 11 (14·1) | 51 (18·8) | 85 (14·5) |
|  |  | Yes | 133 (96·4) | 24 (80·0) | 15 (93·8) | 28 (77·8) | 14 (82·4) | 67 (85·9) | 221 (81·2) | 502 (85·5) |
| Seizure | 587 (100·0) | No | 53 (38·4) | 5 (16·7) | 8 (50·0) | 12 (33·3) | 9 (52·9) | 24 (30·8) | 73 (26·8) | 184 (31·3) |
|  |  | Yes | 85 (61·6) | 25 (83·3) | 8 (50·0) | 24 (66·7) | 8 (47·1) | 54 (69·2) | 199 (73·2) | 403 (68·7) |
| Personality/ Behavioural changes | 587 (100·0) | No | 108 (78·3) | 26 (86·7) | 11 (68·8) | 30 (83·3) | 14 (82·4) | 57 (73·1) | 197 (72·4) | 443 (75·5) |
|  |  | Yes | 30 (21·7) | 4 (13·3) | 5 (31·2) | 6 (16·7) | 3 (17·6) | 21 (26·9) | 75 (27·6) | 144 (24·5) |
| Irrelevant/Abnormal talk | 587 (100·0) | No | 123 (89·1) | 27 (90·0) | 12 (75·0) | 31 (86·1) | 13 (76·5) | 67 (85·9) | 247 (90·8) | 520 (88·6) |
|  |  | Yes | 15 (10·9) | 3 (10·0) | 4 (25·0) | 5 (13·9) | 4 (23·5) | 11 (14·1) | 25 (9·2) | 67 (11·4) |
| New abnormal speech (e·g·, slurred) including the inability to speak | 587 (100·0) | No | 114 (82·6) | 28 (93·3) | 10 (62·5) | 29 (80·6) | 12 (70·6) | 54 (69·2) | 219 (80·5) | 466 (79·4) |
|  |  | Yes | 24 (17·4) | 2 (6·7) | 6 (37·5) | 7 (19·4) | 5 (29·4) | 24 (30·8) | 53 (19·5) | 121 (20·6) |
| Musculoskeletal symptoms (muscle/ joint pain) | 587 (100·0) | No | 128 (92·8) | 29 (96·7) | 12 (75·0) | 33 (91·7) | 15 (88·2) | 72 (92·3) | 265 (97·4) | 554 (94·4) |
|  |  | Yes | 10 (7·2) | 1 (3·3) | 4 (25·0) | 3 (8·3) | 2 (11·8) | 6 (7·7) | 7 (2·6) | 33 (5·6) |
| Respiratory symptoms (cough and/or difficulty breathing) | 587 (100·0) | No | 119 (86·2) | 22 (73·3) | 15 (93·8) | 32 (88·9) | 17 (100·0) | 68 (87·2) | 217 (79·8) | 490 (83·5) |
|  |  | Yes | 19 (13·8) | 8 (26·7) | 1 (6·2) | 4 (11·1) | 0 (0·0) | 10 (12·8) | 55 (20·2) | 97 (16·5) |
| Diarrhoea | 587 (100·0) | No | 127 (92·0) | 29 (96·7) | 14 (87·5) | 34 (94·4) | 13 (76·5) | 73 (93·6) | 240 (88·2) | 530 (90·3) |
|  |  | Yes | 11 (8·0) | 1 (3·3) | 2 (12·5) | 2 (5·6) | 4 (23·5) | 5 (6·4) | 32 (11·8) | 57 (9·7) |
| Abdominal pain | 587 (100·0) | No | 99 (71·7) | 28 (93·3) | 14 (87·5) | 35 (97·2) | 17 (100·0) | 69 (88·5) | 256 (94·1) | 518 (88·2) |
|  |  | Yes | 39 (28·3) | 2 (6·7) | 2 (12·5) | 1 (2·8) | 0 (0·0) | 9 (11·5) | 16 (5·9) | 69 (11·8) |
| Abdominal distension | 587 (100·0) | No | 117 (84·8) | 26 (86·7) | 13 (81·2) | 33 (91·7) | 16 (94·1) | 68 (87·2) | 253 (93·0) | 526 (89·6) |
|  |  | Yes | 21 (15·2) | 4 (13·3) | 3 (18·8) | 3 (8·3) | 1 (5·9) | 10 (12·8) | 19 (7·0) | 61 (10·4) |
| Gastrointestinal symptoms (diarrhoea/ abdominal pain/ abdominal distension) | 587 (100·0) | No | 82 (59·4) | 23 (76·7) | 10 (62·5) | 31 (86·1) | 12 (70·6) | 59 (75·6) | 209 (76·8) | 426 (72·6) |
|  |  | Yes | 56 (40·6) | 7 (23·3) | 6 (37·5) | 5 (13·9) | 5 (29·4) | 19 (24·4) | 63 (23·2) | 161 (27·4) |
| Lymphadenopathy | 587 (100·0) | No | 114 (82·6) | 29 (96·7) | 15 (93·8) | 34 (94·4) | 17 (100·0) | 74 (94·9) | 265 (97·4) | 548 (93·4) |
|  |  | Yes | 24 (17·4) | 1 (3·3) | 1 (6·2) | 2 (5·6) | 0 (0·0) | 4 (5·1) | 7 (2·6) | 39 (6·6) |
| Icterus | 587 (100·0) | No | 128 (92·8) | 29 (96·7) | 16 (100·0) | 35 (97·2) | 16 (94·1) | 76 (97·4) | 266 (97·8) | 566 (96·4) |
|  |  | Yes | 10 (7·2) | 1 (3·3) | 0 (0·0) | 1 (2·8) | 1 (5·9) | 2 (2·6) | 6 (2·2) | 21 (3·6) |
| Oedema | 587 (100·0) | No | 102 (73·9) | 26 (86·7) | 16 (100·0) | 34 (94·4) | 16 (94·1) | 71 (91·0) | 254 (93·4) | 519 (88·4) |
|  |  | Yes | 36 (26·1) | 4 (13·3) | 0 (0·0) | 2 (5·6) | 1 (5·9) | 7 (9·0) | 18 (6·6) | 68 (11·6) |
| Conjunctival involvement (conjunctivitis/ subconjunctival haemorrhage) | 587 (100·0) | No | 120 (87·0) | 28 (93·3) | 14 (87·5) | 35 (97·2) | 17 (100·0) | 70 (89·7) | 261 (96·0) | 545 (92·8) |
|  |  | Yes | 18 (13·0) | 2 (6·7) | 2 (12·5) | 1 (2·8) | 0 (0·0) | 8 (10·3) | 11 (4·0) | 42 (7·2) |
| Rash (including eschar and purpura fulminans) | 587 (100·0) | No | 116 (84·1) | 25 (83·3) | 13 (81·2) | 29 (80·6) | 14 (82·4) | 66 (84·6) | 240 (88·2) | 503 (85·7) |
|  |  | Yes | 22 (15·9) | 5 (16·7) | 3 (18·8) | 7 (19·4) | 3 (17·6) | 12 (15·4) | 32 (11·8) | 84 (14·3) |
| Hepatomegaly | 587 (100·0) | No | 70 (50·7) | 21 (70·0) | 11 (68·8) | 29 (80·6) | 11 (64·7) | 62 (79·5) | 211 (77·6) | 415 (70·7) |
|  |  | Yes | 68 (49·3) | 9 (30·0) | 5 (31·2) | 7 (19·4) | 6 (35·3) | 16 (20·5) | 61 (22·4) | 172 (29·3) |
| Splenomegaly | 587 (100·0) | No | 112 (81·2) | 27 (90·0) | 12 (75·0) | 33 (91·7) | 17 (100·0) | 76 (97·4) | 259 (95·2) | 536 (91·3) |
|  |  | Yes | 26 (18·8) | 3 (10·0) | 4 (25·0) | 3 (8·3) | 0 (0·0) | 2 (2·6) | 13 (4·8) | 51 (8·7) |
| Hepatosplenomegaly | 587 (100·0) | No | 112 (81·2) | 27 (90·0) | 12 (75·0) | 33 (91·7) | 17 (100·0) | 76 (97·4) | 259 (95·2) | 536 (91·3) |
|  |  | Yes | 26 (18·8) | 3 (10·0) | 4 (25·0) | 3 (8·3) | 0 (0·0) | 2 (2·6) | 13 (4·8) | 51 (8·7) |
| Ascites | 587 (100·0) | No | 133 (96·4) | 29 (96·7) | 16 (100·0) | 36 (100·0) | 17 (100·0) | 76 (97·4) | 271 (99·6) | 578 (98·5) |
|  |  | Yes | 5 (3·6) | 1 (3·3) | 0 (0·0) | 0 (0·0) | 0 (0·0) | 2 (2·6) | 1 (0·4) | 9 (1·5) |
| Presence of cerebellar signs (truncal ataxia/ gait abnormality/ finger-nose incoordination/ nystagmus/ dysdiadochokinesia/ dysarthria) | 587 (100·0) | No | 119 (86·2) | 30 (100·0) | 12 (75·0) | 30 (83·3) | 17 (100·0) | 69 (88·5) | 256 (94·1) | 533 (90·8) |
|  |  | Yes | 19 (13·8) | 0 (0·0) | 4 (25·0) | 6 (16·7) | 0 (0·0) | 9 (11·5) | 16 (5·9) | 54 (9·2) |
| Signs of meningeal irritation ( | 587 (100·0) | No | 79 (57·2) | 26 (86·7) | 11 (68·8) | 23 (63·9) | 11 (64·7) | 47 (60·3) | 195 (71·7) | 392 (66·8) |
| nuchal rigidity, Kernig’s sign, Brudzinski sign, bulging of anterior fontanelle in infants) |  | Yes | 59 (42·8) | 4 (13·3) | 5 (31·2) | 13 (36·1) | 6 (35·3) | 31 (39·7) | 77 (28·3) | 195 (33·2) |
| Cranial Nerve Abnormality | 587 (100·0) | No | 129 (93·5) | 30 (100·0) | 13 (81·2) | 31 (86·1) | 13 (76·5) | 70 (89·7) | 253 (93·0) | 539 (91·8) |
|  |  | Yes | 9 (6·5) | 0 (0·0) | 3 (18·8) | 5 (13·9) | 4 (23·5) | 8 (10·3) | 19 (7·0) | 48 (8·2) |
| Involuntary movements | 587 (100·0) | No | 129 (93·5) | 28 (93·3) | 14 (87·5) | 32 (88·9) | 17 (100·0) | 70 (89·7) | 250 (91·9) | 540 (92·0) |
|  |  | Yes | 9 (6·5) | 2 (6·7) | 2 (12·5) | 4 (11·1) | 0 (0·0) | 8 (10·3) | 22 (8·1) | 47 (8·0) |
| Haemoglobin(g/dL) | 578 (98·5) | Median (IQR) | 10·3 (9·1–11·2) | 10·2 (9·1–11·4) | 11·2 (10·0–13·3) | 11·8 (10·5–12·9) | 10·9 (8·5–11·9) | 11·0 (9·6–12·0) | 10·8 (9·4–11·8) | 10·7 (9·4–11·7) |
| Total white blood cell count (× 10^9^/L) | 583 (99·3) | Median (IQR) | 11·5 (7·8–14·4) | 11·0 (8·1–12·3) | 9·2 (6·9–19·4) | 10·6 (7·1–13·2) | 11·0 (6·9–14·3) | 10·3 (7·2–14·4) | 11·3 (7·8–15·8) | 11·1 (7·7–15·0) |
| Absolute Lymphocyte Count (× 10^9^/L) | 577 (98·3) | Median (IQR) | 4·0 (2·4–5·5) | 2·5 (1·5–3·9) | 2·1 (1·5–4·2) | 2·4 (1·3–3·4) | 1·9 (1·0–2·9) | 2·4  (1·6–3.6) | 2·4 (1·5–4·3) | 2·6 (1·6–4·5) |
| Absolute Neutrophil Count (× 10^9^/L) | 576 (98·1) | Median (IQR) | 6·3 (4·2–8·6) | 7·0 (5·1–9·5) | 6·1 (4·8–13·1) | 7·0 (4·9–9·9) | 6·9 (4·2–10·3) | 6·3 (3·8–9·4) | 7·1 (4·2–11·3) | 6·6 (4·2–10·0) |
| Neutrophil/ Lymphocyte ratio | 575 (98·0) | Median (IQR) | 1·7  (1·0–2·6) | 2·7 (1·6–3·6) | 2·7 (2·1–4·6) | 3·0 (1·6–5·5) | 2·7 (2·1–7·6) | 2·5 (1·2–4·4) | 2·7 (1·3–5·6) | 2·4 (1·2–4·8) |
| Platelets count (× 10^9^/L) | 582 (99·1) | Median (IQR) | 123·0 (63·0–191·0) | 186·0 (58·2–244·0) | 254·0 (168·5–416·8) | 271·5 (172·5–367·5) | 209·0 (135·0–279·0) | 244·5 (138·8–352·0) | 282·5 (164·2–393·8) | 216·0 (117·2–338·8) |
| Total Bilirubin (mg/dL) | 503 (85·7) | Median (IQR) | 0·5 (0·3–1·3) | 0·5 (0·3–0·8) | 0·3 (0·2–0·7) | 0·3 (0·2–0·4) | 0·4 (0·3–0·7) | 0·3 (0·2–0·4) | 0·3 (0·2–0·6) | 0·4 (0·2–0·7) |
| Direct Bilirubin (mg/dL) | 505 (86·0) | Median (IQR) | 0·3 (0·2–1·0) | 0·2 (0·1–0·4) | 0·1 (0·1–0·3) | 0·1 (0·1–0·2) | 0·2 (0·1–0·5) | 0·1 (0·1–0·2) | 0·1 (0·1–0·3) | 0·2 (0·1–0·3) |
| Aspartate transaminase (AST) (IU/L) | 552 (94·0) | Median (IQR) | 106·9 (63·1–226·1) | 61·0 (43·3–205·4) | 40·0 (24·1–86·9) | 29·8 (25·4–43·0) | 41·5 (21·1–92·0) | 43·0 (28·3–102·1) | 42·2 (29·0–73·4) | 51·1 (31·3–111·0) |
| Alanine transaminase (ALT) (IU/L) | 552 (94·0) | Median (IQR) | 69·0 (47·0–125·4) | 37·2 (22·0–231·8) | 29·0 (16·9–68·2) | 21·5 (12·9–36·8) | 40·9 (14·7–65·0) | 22·7 (13·5–59·7) | 22·0 (12·9–39·0) | 30·2 (15·4–69·2) |
| Transaminitis | 552 (94·0) | No | 44 (31·9) | 14 (46·7) | 10 (62·5) | 28 (77·8) | 11 (64·7) | 49 (62·8) | 191 (70·2) | 347 (59·1) |
| (AST/ ALT > 80 IU/L) |  | Yes | 91 (65·9) | 15 (50·0) | 5 (31·2) | 7 (19·4) | 6 (35·3) | 20 (25·6) | 61 (22·4) | 205 (34·9) |
| Total Protein (g/dL) | 509 (86·7) | Median (IQR) | 5·9 (5·3–6·4) | 6·1 (4·5–6·7) | 6·6 (5·7–6·9) | 6·8 (6·1–7·1) | 6·1 (5·4–6·4) | 6·5 (5·7–7·1) | 6·3 (5·8–6·8) | 6·2 (5·6–6·9) |
| Serum albumin (g/dL) | 509 (86·7) | Median (IQR) | 2·8 (2·5–3·2) | 3·3 (2·7–4·0) | 3·7 (3·3–4·2) | 3·9 (3·2–4·4) | 3·7 (3·4–3·9) | 3·6 (2·9–4·1) | 3·8 (3·3–4·3) | 3·5 (2·9–4·1) |
| Urea (mg/dL) | 521 (88·8) | Median (IQR) | 25·0 (18·0–33·3) | 33·7 (17·2–43·7) | 29·3 (22·7–47·6) | 26·1 (18·9–39·4) | 23·2 (20·1–36·0) | 22·3 (17·1–32·4) | 22·4 (15·6–34·5) | 23·5 (17·0–34·7) |
| Creatinine (mg/dL) | 566 (96·4) | Median (IQR) | 0·4 (0·3–0·5) | 0·4 (0·3–0·6) | 0·7 (0·5–0·8) | 0·5 (0·4–0·7) | 0·5 (0·3–0·6) | 0·4 (0·3–0·6) | 0·4 (0·3–0·5) | 0·4 (0·3–0·6) |
| Sodium (mEq/L) | 581 (99·0) | Median (IQR) | 136·0 (133·0–138·0) | 137·0 (131·2–141·5) | 137·5 (135·8–142·2) | 136·0 (134·0–140·0) | 137·0 (134·0–138·0) | 135·5 (133·0–138·0) | 137·0 (135·0–140·0) | 136·0 (134·0–139·0) |
| Potassium (mEq/L) | 581 (99·0) | Median (IQR) | 4·4 (4·1 – 4·8) | 4·5 (4·3–5·2) | 4·3 (3·7–4·9) | 4·5 (4·0–4·6) | 4·3 (4·0–4·4) | 4·4 (4·1–4·7) | 4·4 (3·9–4·8) | 4·4 (4·0–4·8) |
| Chloride (mEq/L) | 570 (97·1) | Median (IQR) | 101·9 (98·0–105·6) | 103·5 (99·9–111·5) | 108·4 (100·8–112·0) | 100·4 (97·5–105·5) | 101·5 (99·9–104·0) | 100·7 (97·1–103·6) | 103·2 (99·1–106·8) | 102·3 (98·5–106·0) |
| CSF total leucocyte count (cells/µL) | 529 (90·1) | Median (IQR) | 14·0 (4·0 – 49·2) | 1·0 (0·0–5·5) | 3·5 (2·0–8·0) | 3·0 (0·0–9·0) | 9·0 (1·0–50·0) | 6·0 (1·0–41·0) | 2·0 (0·0–12·0) | 4·0 (1·0–27·0) |
| CSF lymphocyte count (cells/µL) | 529 (90·1) | Median (IQR) | 13·0 (4·0–36·0) | 1·0 (0·0–5·5) | 3·0 (2·0–7·8) | 3·0 (0·0–6·0) | 7·0 (1·0–25·0) | 5·0 (1·0–35·0) | 2·0 (0·0–10·0) | 4·0 (1·0–22·0) |
| CSF neutrophil count (cells/µL) | 529 (90·1) | Median (IQR) | 0·0 (0·0–3·0) | 0·0 (0·0–0·0) | 0·0 (0·0–0·8) | 0·0 (0·0–0·0) | 0·0 (0·0–0·0) | 0·0 (0·0–4·0) | 0·0 (0·0–0·0) | 0·0 (0·0–2·0) |
| CSF pleocytosis | 529 (90·1) | No | 32 (23·2) | 15 (50·0) | 9 (56·2) | 21 (58·3) | 8 (47·1) | 33 (42·3) | 160 (58·8) | 278 (47·4) |
|  |  | Yes | 92 (66·7) | 8 (26·7) | 5 (31·2) | 12 (33·3) | 9 (52·9) | 40 (51·3) | 85 (31·2) | 251 (42·8) |
| CSF protein concentration (mg/dL) | 536 (91·3) | Median (IQR) | 60·5 (43·8–94·6) | 19·6 (15·8–39·9) | 25·0 (17·2–40·0) | 31·4 (18·7–46·0) | 42·7 (21·2–58·0) | 31·0 (17·9–57·0) | 26·9 (16·8–50·0) | 33·4 (19·0–62·2) |
| CSF Glucose (mg/dL) | 537 (91·5) | Median (IQR) | 53·8 (48·8–61·9) | 61·6 (48·6–69·6) | 61·5 (55·2–68·6) | 66·5 (57·5–71·7) | 59·4 (51·0–72·2) | 58·8 (48·6–66·7) | 63·6 (54·8–73·9) | 60·4 (51·1–70·0) |
| Duration of hospitalisation (days) | 547 (93·2) | Median (IQR) | 9·0 (6·0–11·0) | 10·0 (7·0–14·2) | 7·5 (6·0–9·0) | 13·0 (6·0–21·8) | 19·0 (9·0–28·0) | 10·0 (7·5–15·5) | 9·0 (6·0–15·0) | 9·0 (7·0–15·0) |
| Managed in intensive care unit during hospitalisation | 587 (100·0) | No | 60 (43·5) | 10 (33·3) | 5 (31·2) | 18 (50·0) | 6 (35·3) | 35 (44·9) | 101 (37·1) | 235 (40·0) |
|  |  | Yes | 78 (56·5) | 20 (66·7) | 11 (68·8) | 18 (50·0) | 11 (64·7) | 43 (55·1) | 171 (62·9) | 352 (60·0) |
| Liverpool Outcome Score | 434 (73·9) | Death | 8 (5·8) | 6 (20·0) | 2 (12·5) | 3 (8·3) | 0 (0·0) | 3 (3·8) | 29 (10·7) | 51 (8·7) |
|  |  | Severe | 3 (2·2) | 0 (0·0) | 1 (6·2) | 3 (8·3) | 3 (17·6) | 6 (7·7) | 18 (6·6) | 34 (5·8) |
|  |  | Moderate | 23 (16·7) | 4 (13·3) | 1 (6·2) | 5 (13·9) | 3 (17·6) | 9 (11·5) | 31 (11·4) | 76 (12·9) |
|  |  | Mild | 11 (8·0) | 1 (3·3) | 0 (0·0) | 1 (2·8) | 1 (5·9) | 8 (10·3) | 18 (6·6) | 40 (6·8) |
|  |  | Normal | 69 (50·0) | 12 (40·0) | 8 (50·0) | 10 (27·8) | 8 (47·1) | 31 (39·7) | 95 (34·9) | 233 (39·7) |

*ST= Scrub typhus, DEN= Dengue, CHIK= Chikungunya

**Patient outcome**

In the Liverpool outcome scoring system, a score of 1 indicates death, scores of 2–4 represent varying degrees of neurological sequelae (with 2 being severe, 3 moderate, and 4 minor), and a score of 5 denotes complete recovery.

The study identified several significant differences between children with favourable (LOS 4 & 5) and unfavourable outcomes (LOS 1-3) after three months of discharge. A longer duration of hospitalisation was associated with unfavourable outcomes (median 13 days) compared to favourable ones [13 IQR ( 8-21) vs 9 (6-12) days (p-value < 0·001). Children admitted to intensive care had a significantly higher likelihood of an unfavourable outcome (67·1%) than those not requiring intensive care (44%) (p = 0·030). Neurological assessments showed that a Glasgow Coma Scale score of ≤8 at admission was more frequent in unfavourable cases (31·7%) than favourable ones (13·2%), (p < 0·001). Conjunctival involvement, rash, hepatomegaly and signs of meningeal irritation were more common in children with favourable outcomes, whereas involuntary movements were more commonly associated in children with unfavourable outcomes. There were no statistically significant differences in the proportion of unfavorable outcomes across different aetiologies; however, among those with unfavorable outcomes, 34 children (21·1%) were diagnosed with scrub typhus, and 78 children (48%) had an unidentifiable aetiology (**Table S5**).

Among the 51 children who died, 41 (80%) succumbed during hospitalisation. The causes of death, as documented in discharge summaries, were predominantly multiorgan dysfunction with shock or severe sepsis. Of the remaining 10 children (20%), three were on ventilatory support and were discharged against medical advice; they died within 1-2 days after discharge. For the remaining seven children, the cause of death and the median duration from hospital discharge to death could not be ascertained.

**Table S5**: **Demographic, clinical characteristics and laboratory parameters of cases with favourable and unfavourable outcomes after 3 months of discharge (n=434)**

| **Clinical/ Laboratory Variables** | **No patients (%)** |  | **Favourable Outcome** | **Unfavourable Outcome** | **Total** | **P value** |
| --- | --- | --- | --- | --- | --- | --- |
| Total N (%) |  |  | 273 (62·9) | 161 (37·1) | 434 |  |
| Admitted to | 434 (100·0) | IGICH | 164 (60·1) | 120 (74·5) | 284 (65·4) | **<0·001** |
|  |  | SJMC | 88 (32·2) | 23 (14·3) | 111 (25·6) |  |
|  |  | BMC | 21 (7·7) | 18 (11·2) | 39 (9·0) |  |
| Age (years) | 434 (100·0) | Median (IQR) | 6·0 (2·5–10·0) | 5·0 (1·7–9·5) | 6·0 (2·0–10·0) | 0·162 |
| Age categories | 434 (100·0) | 28 days-1 year | 26 (9·5) | 17 (10·6) | 43 (9·9) | 0·598 |
|  |  | 1-5 years | 85 (31·1) | 61 (37·9) | 146 (33·6) |  |
|  |  | 5-10 years | 84 (30·8) | 43 (26·7) | 127 (29·3) |  |
|  |  | 10-15 year | 65 (23·8) | 32 (19·9) | 97 (22·4) |  |
|  |  | >15 years | 13 (4·8) | 8 (5·0) | 21 (4·8) |  |
| Duration of Illness (days) | 434 (100·0) | Median (IQR) | 5·0 (3·0–7·0) | 5·0 (3·0–7·0) | 5·0 (3·0–7·0) | 0·697 |
| Duration of Illness (>/=5days) | 434 (100·0) | No | 122 (44·7) | 69 (42·9) | 191 (44·0) | 0·786 |
|  |  | Yes | 151 (55·3) | 92 (57·1) | 243 (56·0) |  |
| Managed in intensive care unit during hospitalisation | 434 (100·0) | No | 120 (44·0) | 53 (32·9) | 173 (39·9) | **0·030** |
|  |  | Yes | 153 (56·0) | 108 (67·1) | 261 (60·1) |  |
| Duration of hospitalisation (days) | 408 (94·0) | Median (IQR) | 9·0 (6·0–12·0) | 13·0 (8·0–21·0) | 9·0 (7·0–15·0) | **<0·001** |
| Diagnosis | 434 (100·0) | Scrub typhus | 80 (29·3) | 34 (21·1) | 114 (26·3) | 0·362 |
|  |  | Dengue | 13 (4·8) | 10 (6·2) | 23 (5·3) |  |
|  |  | Chikungunya | 8 (2·9) | 4 (2·5) | 12 (2·8) |  |
|  |  | JEV | 11 (4·0) | 11 (6·8) | 22 (5·1) |  |
|  |  | Leptospira | 9 (3·3) | 6 (3·7) | 15 (3·5) |  |
|  |  | Others | 39 (14·3) | 18 (11·2) | 57 (13·1) |  |
|  |  | Unknown | 113 (41·4) | 78 (48·4) | 191 (44·0) |  |
| GCS score at admission | 361 (83·2) | 15 | 82 (30·0) | 31 (19·3) | 113 (26·0) | **<0·001** |
|  |  | >8-14 | 104 (38·1) | 57 (35·4) | 161 (37·1) |  |
|  |  | </=8 | 36 (13·2) | 51 (31·7) | 87 (20·0) |  |
| Anti-infective treatment received before presentation to hospital | 426 (98·2) | No | 141 (51·6) | 84 (52·2) | 225 (51·8) | 0·994 |
|  |  | Yes | 127 (46·5) | 74 (46·0) | 201 (46·3) |  |
| Referred from another hospital | 426 (98·2) | No | 105 (38·5) | 54 (33·5) | 159 (36·6) | 0·588 |
|  |  | Yes | 163 (59·7) | 104 (64·6) | 267 (61·5) |  |
| Gender | 434 (100·0) | Male | 151 (55·3) | 89 (55·3) | 240 (55·3) | 1·000 |
|  |  | Female | 122 (44·7) | 72 (44·7) | 194 (44·7) |  |
| Seizure | 434 (100·0) | No | 94 (34·4) | 49 (30·4) | 143 (32·9) | 0·453 |
|  |  | Yes | 179 (65·6) | 112 (69·6) | 291 (67·1) |  |
| Fever as first presentation | 434 (100·0) | No | 33 (12·1) | 29 (18·0) | 62 (14·3) | 0·118 |
|  |  | Yes | 240 (87·9) | 132 (82·0) | 372 (85·7) |  |
| Personality/ Behavioural changes | 434 (100·0) | No | 214 (78·4) | 116 (72·0) | 330 (76·0) | 0·168 |
|  |  | Yes | 59 (21·6) | 45 (28·0) | 104 (24·0) |  |
| Irrelevant/Abnormal Talk | 434 (100·0) | No | 243 (89·0) | 138 (85·7) | 381 (87·8) | 0·389 |
|  |  | Yes | 30 (11·0) | 23 (14·3) | 53 (12·2) |  |
| New abnormal speech | 434 (100·0) | No | 219 (80·2) | 123 (76·4) | 342 (78·8) | 0·412 |
|  |  | Yes | 54 (19·8) | 38 (23·6) | 92 (21·2) |  |
| Musculoskeletal symptoms | 434 (100·0) | No | 252 (92·3) | 155 (96·3) | 407 (93·8) | 0·148 |
|  |  | Yes | 21 (7·7) | 6 (3·7) | 27 (6·2) |  |
| Respiratory symptoms | 434 (100·0) | No | 228 (83·5) | 137 (85·1) | 365 (84·1) | 0·766 |
|  |  | Yes | 45 (16·5) | 24 (14·9) | 69 (15·9) |  |
| Gastrointestinal symptoms | 434 (100·0) | No | 193 (70·7) | 117 (72·7) | 310 (71·4) | 0·741 |
|  |  | Yes | 80 (29·3) | 44 (27·3) | 124 (28·6) |  |
| Lymphadenopathy | 434 (100·0) | No | 250 (91·6) | 153 (95·0) | 403 (92·9) | 0·247 |
|  |  | Yes | 23 (8·4) | 8 (5·0) | 31 (7·1) |  |
| Icterus | 434 (100·0) | No | 266 (97·4) | 151 (93·8) | 417 (96·1) | 0·102 |
|  |  | Yes | 7 (2·6) | 10 (6·2) | 17 (3·9) |  |
| Oedema | 434 (100·0) | No | 235 (86·1) | 146 (90·7) | 381 (87·8) | 0·207 |
|  |  | Yes | 38 (13·9) | 15 (9·3) | 53 (12·2) |  |
| Conjunctival involvement | 434 (100·0) | No | 248 (90·8) | 156 (96·9) | 404 (93·1) | **0·027** |
|  |  | Yes | 25 (9·2) | 5 (3·1) | 30 (6·9) |  |
| Rash | 434 (100·0) | No | 219 (80·2) | 147 (91·3) | 366 (84·3) | **0·003** |
|  |  | Yes | 54 (19·8) | 14 (8·7) | 68 (15·7) |  |
| Hepatomegaly | 434 (100·0) | No | 175 (64·1) | 119 (73·9) | 294 (67·7) | **0·045** |
|  |  | Yes | 98 (35·9) | 42 (26·1) | 140 (32·3) |  |
| Splenomegaly | 434 (100·0) | No | 243 (89·0) | 145 (90·1) | 388 (89·4) | 0·855 |
|  |  | Yes | 30 (11·0) | 16 (9·9) | 46 (10·6) |  |
| Hepatosplenomegaly | 434 (100·0) | No | 243 (89·0) | 145 (90·1) | 388 (89·4) | 0·855 |
|  |  | Yes | 30 (11·0) | 16 (9·9) | 46 (10·6) |  |
| Ascites | 434 (100·0) | No | 266 (97·4) | 159 (98·8) | 425 (97·9) | 0·559 |
|  |  | Yes | 7 (2·6) | 2 (1·2) | 9 (2·1) |  |
| Presence of cerebellar signs | 434 (100·0) | No | 243 (89·0) | 147 (91·3) | 390 (89·9) | 0·548 |
|  |  | Yes | 30 (11·0) | 14 (8·7) | 44 (10·1) |  |
| Signs of meningeal irritation | 434 (100·0) | No | 175 (64·1) | 120 (74·5) | 295 (68·0) | **0·032** |
|  |  | Yes | 98 (35·9) | 41 (25·5) | 139 (32·0) |  |
| Cranial Nerve Abnormality | 434 (100·0) | No | 254 (93·0) | 140 (87·0) | 394 (90·8) | 0·052 |
|  |  | Yes | 19 (7·0) | 21 (13·0) | 40 (9·2) |  |
| Involuntary movements | 434 (100·0) | No | 260 (95·2) | 142 (88·2) | 402 (92·6) | **0·012** |
|  |  | Yes | 13 (4·8) | 19 (11·8) | 32 (7·4) |  |
| Haemoglobin(g/dL) | 428 (98·6) | Median (IQR) | 10·7 (9·4–11·7) | 10·5 (8·9–11·5) | 10·6 (9·3–11·6) | 0·205 |
| –Total white blood cell count (× 10^9^/L) | 431 (99·3) | Median (IQR) | 11·5 (7·7–15·4) | 11·1 (7·6–14·6) | 11·2 (7·7–15·0) | 0·396 |
| Absolute Lymphocyte Count (× 10^9^/L) | 428 (98·6) | Median (IQR) | 2·8 (1·6–4·6) | 2·5 (1·6–4·6) | 2·7 (1·6–4·6) | 0·414 |
| Absolute Neutrophil Count (× 10^9^/L) | 426 (98·2) | Median (IQR) | 7·0 (4·6–10·0) | 6·5 (3·7–10·3) | 6·9 (4·2–10·1) | 0·256 |
| Neutrophil/ Lymphocyte ratio | 426 (98·2) | Median (IQR) | 2·5 (1·3–4·2) | 2·1 (1·1–4·8) | 2·3 (1·2–4·4) | 0·555 |
| Platelets count (× 10^9^/L) | 430 (99·1) | Median (IQR) | 200·0 (105·0–330·0) | 234·0 (137·0–366·0) | 212·0 (119·2–343·8) | 0·052 |
| Total Bilirubin(mg/dL) | 372 (85·7) | Median (IQR) | 0·4 (0·2–0·8) | 0·3 (0·2–0·7) | 0·4 (0·2–0·8) | 0·060 |
| Direct Bilirubin (mg/dL) | 375 (86·4) | Median (IQR) | 0·2 (0·1–0·4) | 0·1 (0·1–0·3) | 0·2 (0·1–0·3) | **0·010** |
| AST (IU/L) | 411 (94·7) | Median (IQR) | 54·7 (32·8–115·8) | 48·0 (27·2–104·0) | 53·2 (32·0–112·7) | 0·204 |
| ALT (IU/L) | 411 (94·7) | Median (IQR) | 36·1 (17·4–68·8) | 27·0 (14·7–71·8) | 32·0 (16·0–71·0) | 0·183 |
| Transaminitis | 411 (94·7) | No | 163 (59·7) | 96 (59·6) | 259 (59·7) | 0·109 |
|  |  | Yes | 91 (33·3) | 61 (37·9) | 152 (35·0) |  |
| Total Protein (g/dL) | 376 (86·6) | Median (IQR) | 6·2 (5·4–6·7) | 6·4 (5·7–7·1) | 6·3 (5·5–6·9) | **0·020** |
| Serum albumin (g/dL) | 375 (86·4) | Median (IQR) | 3·4 (2·8–4·0) | 3·5 (3·0–4·1) | 3·4 (2·8–4·1) | 0·214 |
| Urea (mg/dL) | 384 (88·5) | Median (IQR) | 23·6 (17·4–32·6) | 23·9 (16·5–36·9) | 23·8 (17·1–34·1) | 0·529 |
| Creatinine(mg/dL) | 420 (96·8) | Median (IQR) | 0·4 (0·3–0·5) | 0·4 (0·3–0·6) | 0·4 (0·3–0·6) | 0·592 |
| Sodium (mEq/L) | 430 (99·1) | Median (IQR) | 136·0 (134·0–138·0) | 137·0 (134·0–140·0) | 137·0 (134·0–139·0) | **0·002** |
| Potassium (mEq/L) | 430 (99·1) | Median (IQR) | 4·4 (4·1–4·8) | 4·4 (4·0–4·7) | 4·4 (4·0–4·8) | 0·420 |
| Chloride (mEq/L) | 423 (97·5) | Median (IQR) | 102·0 (98·3–105·6) | 102·4 (99·0–107·0) | 102·2 (98·3–106·0) | 0·171 |
| CSF –total leucocyte count (cells/µL) | 396 (91·2) | Median (IQR) | 5·0 (1·0–37·0) | 5·0 (1·0–21·0) | 5·0 (1·0–28·2) | 0·220 |
| CSF lymphocyte count (cells/µL) | 396 (91·2) | Median (IQR) | 5·0 (1·0–26·0) | 4·0 (1·0–20·0) | 5·0 (1·0–23·2) | 0·173 |
| CSF neutrophil count (cells/µL) | 396 (91·2) | Median (IQR) | 0·0 (0·0–1·0) | 0·0 (0·0–2·0) | 0·0 (0·0–2·0) | 0·622 |
| CSF pleocytosis | 396 (91·2) | No | 121 (44·3) | 74 (46·0) | 195 (44·9) | 0·691 |
|  |  | Yes | 130 (47·6) | 71 (44·1) | 201 (46·3) |  |
| CSF protein concentration (mg/dL) | 401 (92·4) | Median (IQR) | 36·7 (18·3–64·9) | 32·0 (21·2–60·9) | 33·4 (19·6–64·0) | 0·964 |
| CSF Glucose(mg/dL) | 401 (92·4) | Median (IQR) | 59·5 (51·0–67·5) | 62·1 (52·2–73·4) | 60·0 (51·4–69·5) | **0·042** |

**Table S6: Distribution of aetiologies included in the development of model for scrub typhus diagnosis (n=271)**

| **Final diagnosis** | **Comparator group** | **Scrub typhus group** | **Total** |
| --- | --- | --- | --- |
| Probable *Orientia tsutsugamushi* |  | 99 | 99 |
| Confirmed *Orientia tsutsugamushi* |  | 28 | 28 |
| Probable JEV | 29 |  | 29 |
| Probable *Leptospira sp* | 17 |  | 17 |
| Probable Dengue virus | 11 |  | 11 |
| Possible JEV, Possible Dengue virus | 9 |  | 9 |
| Possible JEV, Confirmed Dengue virus | 7 |  | 7 |
| Probable Chikungunya virus | 6 |  | 6 |
| Probable Measles | 5 |  | 5 |
| Confirmed JEV | 5 |  | 5 |
| Confirmed Dengue virus | 5 |  | 5 |
| Confirmed HSV-1 | 4 |  | 4 |
| Confirmed *Streptococcus pneumoniae* | 3 |  | 3 |
| Confirmed Enterovirus | 3 |  | 3 |
| Confirmed Chikungunya virus | 3 |  | 3 |
| Possible JEV, Possible *Leptospira sp* | 2 |  | 2 |
| Possible Dengue virus, Possible WNV | 2 |  | 2 |
| Possible Dengue virus, Possible *Leptospira sp* | 2 |  | 2 |
| Possible Chikungunya virus, Possible Dengue virus | 2 |  | 2 |
| Confirmed Mumps | 2 |  | 2 |
| Confirmed Dengue virus, Possible WNV | 2 |  | 2 |
| Probable *Mycobacterium tuberculosis* | 1 |  | 1 |
| Probable JEV, Probable WNV | 1 |  | 1 |
| Probable SARS CoV-2 | 1 |  | 1 |
| Possible *Mycobacterium tuberculosis* | 1 |  | 1 |
| Possible JEV, Probable Dengue virus | 1 |  | 1 |
| Possible JEV, Possible WNV, Probable Dengue virus | 1 |  | 1 |
| Possible JEV, Possible Dengue virus, Confirmed Chikungunya virus | 1 |  | 1 |
| Possible JEV, Confirmed Chikungunya virus | 1 |  | 1 |
| Possible Japanese encephalitis virus, Possible Dengue virus, Possible *Leptospira sp* | 1 |  | 1 |
| Possible Chikungunya virus, Confirmed Dengue virus | 1 |  | 1 |
| Confirmed VZV, Possible JEV | 1 |  | 1 |
| Confirmed VZV | 1 |  | 1 |
| Confirmed TB, Possible Dengue virus | 1 |  | 1 |
| Confirmed TB | 1 |  | 1 |
| Confirmed *Salmonella typhi* | 1 |  | 1 |
| Confirmed MRSA | 1 |  | 1 |
| Confirmed JEV, Possible WNV | 1 |  | 1 |
| Confirmed JEV, Possible Dengue virus | 1 |  | 1 |
| Confirmed HSV-2 | 1 |  | 1 |
| Confirmed *Haemophilus influenzae* | 1 |  | 1 |
| Confirmed Dengue virus, Possible JEV | 1 |  | 1 |
| Confirmed Chikungunya virus, Probable WNV | 1 |  | 1 |
| Confirmed Chikungunya virus, Possible JEV | 1 |  | 1 |
| Confirmed Chikungunya virus, Possible Dengue virus | 1 |  | 1 |
| Confirmed Chikungunya virus, Confirmed Dengue virus | 1 |  | 1 |
| Total | 144 | 127 | 271 |

**Table S7: Demographics and clinical characteristics of children with scrub typhus and other causes of AES (n=271)**

| **Baseline Clinical/ Laboratory variables** | **No patients (%)** |  | **Other causes of AES** | **Scrub typhus** | **Total** | **p value** |
| --- | --- | --- | --- | --- | --- | --- |
| Total N (%) |  |  | 144 (53·1) | 127 (46·9) | 271 |  |
| Age (years) | 271 (100·0) | Median (IQR) | 5·5 (2·1–10·2) | 8·0 (4·0–10·5) | 7·0 (3·0–10·5) | **0·003** |
| Gender | 271 (100·0) | Male | 90 (62·5) | 72 (56·7) | 162 (59·8) | 0·396 |
|  |  | Female | 54 (37·5) | 55 (43·3) | 109 (40·2) |  |
| Duration of Illness (days) | 271 (100·0) | Median (IQR) | 4·0 (2·0–7·0) | 6·0 (4·0–7·0) | 5·0 (3·0–7·0) | **<0·001** |
| GCS score at admission | 227 (83·8) | 15 | 32 (22·2) | 44 (34·6) | 76 (28·0) | **0·026** |
|  |  | 8-14 | 55 (38·2) | 50 (39·4) | 105 (38·7) |  |
|  |  | </=8 | 26 (18·1) | 20 (15·7) | 46 (17·0) |  |
| Seizure | 271 (100·0) | No | 44 (30·6) | 46 (36·2) | 90 (33·2) | 0·390 |
|  |  | Yes | 100 (69·4) | 81 (63·8) | 181 (66·8) |  |
| Personality/  Behavioural changes | 271 (100·0) | No | 115 (79·9) | 101 (79·5) | 216 (79·7) | 1·000 |
|  |  | Yes | 29 (20·1) | 26 (20·5) | 55 (20·3) |  |
| Irrelevant/Abnormal  talk | 271 (100·0) | No | 124 (86·1) | 113 (89·0) | 237 (87·5) | 0·598 |
|  |  | Yes | 20 (13·9) | 14 (11·0) | 34 (12·5) |  |
| New abnormal speech | 271 (100·0) | No | 114 (79·2) | 107 (84·3) | 221 (81·5) | 0·358 |
|  |  | Yes | 30 (20·8) | 20 (15·7) | 50 (18·5) |  |
| Musculoskeletal  symptoms | 271 (100·0) | No | 132 (91·7) | 118 (92·9) | 250 (92·3) | 0·877 |
|  |  | Yes | 12 (8·3) | 9 (7·1) | 21 (7·7) |  |
| Respiratory symptoms | 271 (100·0) | No | 124 (86·1) | 109 (85·8) | 233 (86·0) | 1·000 |
|  |  | Yes | 20 (13·9) | 18 (14·2) | 38 (14·0) |  |
| Diarrhoea | 271 (100·0) | No | 131 (91·0) | 116 (91·3) | 247 (91·1) | 1·000 |
|  |  | Yes | 13 (9·0) | 11 (8·7) | 24 (8·9) |  |
| Gastrointestinal symptoms | 271 (100·0) | No | 110 (76·4) | 75 (59·1) | 185 (68·3) | **0·003** |
|  |  | Yes | 34 (23·6) | 52 (40·9) | 86 (31·7) |  |
| Lymphadenopathy | 271 (100·0) | No | 140 (97·2) | 105 (82·7) | 245 (90·4) | **<0·001** |
|  |  | Yes | 4 (2·8) | 22 (17·3) | 26 (9·6) |  |
| Oedema | 271 (100·0) | No | 134 (93·1) | 96 (75·6) | 230 (84·9) | **<0·001** |
|  |  | Yes | 10 (6·9) | 31 (24·4) | 41 (15·1) |  |
| Conjunctival involvement | 271 (100·0) | No | 136 (94·4) | 110 (86·6) | 246 (90·8) | **0·044** |
|  |  | Yes | 8 (5·6) | 17 (13·4) | 25 (9·2) |  |
| Rash | 271 (100·0) | No | 124 (86·1) | 106 (83·5) | 230 (84·9) | 0·662 |
|  |  | Yes | 20 (13·9) | 21 (16·5) | 41 (15·1) |  |
| Hepatomegaly | 271 (100·0) | No | 112 (77·8) | 60 (47·2) | 172 (63·5) | **<0·001** |
|  |  | Yes | 32 (22·2) | 67 (52·8) | 99 (36·5) |  |
| Splenomegaly | 271 (100·0) | No | 136 (94·4) | 102 (80·3) | 238 (87·8) | **0·001** |
|  |  | Yes | 8 (5·6) | 25 (19·7) | 33 (12·2) |  |
| Hepatosplenomegaly | 271 (100·0) | No | 136 (94·4) | 102 (80·3) | 238 (87·8) | **0·001** |
|  |  | Yes | 8 (5·6) | 25 (19·7) | 33 (12·2) |  |
| Presence of cerebellar signs | 271 (100·0) | No | 133 (92·4) | 111 (87·4) | 244 (90·0) | 0·247 |
|  |  | Yes | 11 (7·6) | 16 (12·6) | 27 (10·0) |  |
| Signs of meningeal irritation | 271 (100·0) | No | 98 (68·1) | 74 (58·3) | 172 (63·5) | 0·123 |
|  |  | Yes | 46 (31·9) | 53 (41·7) | 99 (36·5) |  |
| Cranial nerve Abnormality | 271 (100·0) | No | 130 (90·3) | 118 (92·9) | 248 (91·5) | 0·577 |
|  |  | Yes | 14 (9·7) | 9 (7·1) | 23 (8·5) |  |
| Involuntary movements | 271 (100·0) | No | 132 (91·7) | 118 (92·9) | 250 (92·3) | 0·877 |
|  |  | Yes | 12 (8·3) | 9 (7·1) | 21 (7·7) |  |
| Haemoglobin (g/dL) | 267 (98·5) | Median (IQR) | 10·9 (9·5–12·5) | 10·3 (9·0–11·2) | 10·6 (9·4–11·8) | **0·002** |
| Total white blood cell count (×10^9^/L) | 270 (99·6) | Median (IQR) | 10·7 (7·2–14·1) | 11·6 (8·2–14·7) | 11·0 (7·5–14·4) | 0·212 |
| Absolute lymphocyte count (×10^9^/L) | 270 (99·6) | Median (IQR) | 24·9 (14·3–36·4) | 33·7 (26·4–47·6) | 30·3 (19·1–41·0) | **<0·001** |
| Absolute neutrophil count (×10^9^/L) | 269 (99·3) | Median (IQR) | 68·8 (56·9–80·8) | 60·6 (48·2–69·4) | 64·0 (51·9–75·5) | **<0·001** |
| Neutrophil/ Lymphocyte ratio | 269 (99·3) | Median (IQR) | 2·7 (1·6–5·4) | 1·8 (1·0–2·6) | 2·1 (1·3–3·9) | **<0·001** |
| Platelets count (×10^9^/L) | 269 (99·3) | Median (IQR) | 239·0 (138·5–344·0) | 110·5 (54·0–189·0) | 170·0 (89·0–279·0) | **<0·001** |
| Total bilirubin(mg/dL) | 237 (87·5) | Median (IQR) | 0·3 (0·2–0·5) | 0·5 (0·3–1·4) | 0·4 (0·2–0·9) | **<0·001** |
| Direct bilirubin (mg/dL) | 239 (88·2) | Median (IQR) | 0·2 (0·1–0·3) | 0·3 (0·1–1·1) | 0·2 (0·1–0·6) | **<0·001** |
| AST (IU/L) | 257 (94·8) | Median (IQR) | 39·9 (25·7–91·4) | 114·5 (69·3–237·6) | 72·8 (34·8–162·2) | **<0·001** |
| ALT (IU/L) | 257 (94·8) | Median (IQR) | 25·0 (13·7–45·0) | 70·1 (49·6–130·8) | 45·0 (19·9–108·3) | **<0·001** |
| Transaminitis | 257 (94·8) | No | 93 (64·6) | 38 (29·9) | 131 (48·3) | **<0·001** |
|  |  | Yes | 40 (27·8) | 86 (67·7) | 126 (46·5) |  |
| Total protein (g/dL) | 243 (89·7) | Median (IQR) | 6·4 (5·6–7·1) | 5·8 (5·2–6·4) | 6·1 (5·4–6·9) | **0·002** |
| Serum albumin (g/dL) | 243 (89·7) | Median (IQR) | 3·8 (3·0–4·2) | 2·8 (2·5–3·2) | 3·2 (2·7–3·9) | **<0·001** |
| Urea (mg/dL) | 243 (89·7) | Median (IQR) | 26·1 (18·2–37·1) | 25·4 (18·7–33·4) | 25·7 (18·4–35·1) | 0·388 |
| Creatinine (mg/dL) | 266 (98·2) | Median (IQR) | 0·5 (0·3–0·7) | 0·4 (0·3–0·5) | 0·4 (0·3–0·6) | **0·046** |
| Sodium (mEq/L) | 269 (99·3) | Median (IQR) | 136·0 (133·0–139·0) | 136·0 (133·0–138·0) | 136·0 (133·0–139·0) | 0·981 |
| Potassium (mEq/L) | 269 (99·3) | Median (IQR) | 4·4 (4·0–4·7) | 4·4 (4·1–4·8) | 4·4 (4·0–4·8) | 0·848 |
| Chloride (mEq/L) | 264 (97·4) | Median (IQR) | 102·0 (97·9–106·0) | 102 (98·1–105·6) | 102·0 (98·1–106·0) | 0·775 |
| CSF total leucocyte count (cells/µL) | 245 (90·4) | Median (IQR) | 3·0 (0·0–21·2) | 13·0 (4·0–50·0) | 6·0 (1·0–38·0) | **<0·001** |
| CSF lymphocyte count (cells/µL) | 245 (90·4) | Median (IQR) | 3·0 (0·0–15·2) | 12·0 (4·0–36·0) | 6·0 (1·0–27·0) | **<0·001** |
| CSF neutrophil count (cells/µL) | 245 (90·4) | Median (IQR) | 0·0 (0·0–0·0) | 0·0 (0·0–3·0) | 0·0 (0·0–2·0) | 0·100 |
| CSF pleocytosis | 245 (90·4) | No | 79 (54·9) | 31 (24·4) | 110 (40·6) | **<0·001** |
|  |  | Yes | 53 (36·8) | 82 (64·6) | 135 (49·8) |  |
| CSF protein concentration (mg/dL) | 247 (91·1) | Median (IQR) | 25·7 (16·8–48·9) | 57·5 (41·5–95·4) | 44·0 (21·8–74·8) | **<0·001** |
| CSF Glucose (mg/dL) | 249 (91·9) | Median (IQR) | 61·0 (51·0–70·8) | 53·9 (49·0–62·0) | 57·7 (50·0–67·0) | **0·002** |
| Duration of hospitalisation (days) | 257 (94·8) | Median (IQR) | 10·0 (7·0–19·0) | 9·0 (6·0–11·0) | 9·0 (7·0–14·0) | **0·009** |
| Managed in intensive care unit during hospitalisation | 271 (100·0) | No | 60 (41·7) | 52 (40·9) | 112 (41·3) | 1·000 |
|  |  | Yes | 84 (58·3) | 75 (59·1) | 159 (58·7) |  |
| Liverpool Outcome Score | 206 (76·0) | Death | 13 (9·0) | 7 (5·5) | 20 (7·4) | **0·015** |
|  |  | Severe | 11 (7·6) | 3 (2·4) | 14 (5·2) |  |
|  |  | Moderate | 18 (12·5) | 20 (15·7) | 38 (14·0) |  |
|  |  | Mild | 9 (6·2) | 11 (8·7) | 20 (7·4) |  |
|  |  | Normal | 50 (34·7) | 64 (50·4) | 114 (42·1) |  |

**Table S8: Distribution of aetiologies included in the development of model for diagnosis of doxycycline-treatable causes (n=315)**

| **Final diagnosis** | **Comparator group** | **Doxycycline group** | **Total** |
| --- | --- | --- | --- |
| Probable Orientia tsutsugamushi |  | 99 | 99 |
| Confirmed Orientia tsutsugamushi |  | 28 | 28 |
| Probable Leptospira sp |  | 17 | 17 |
| Possible JEV, Possible Orientia tsutsugamushi |  | 7 | 7 |
| Probable Rickettsia (Typhus Group) |  | 4 | 4 |
| Probable Orientia tsutsugamushi, Possible Leptospira sp |  | 4 | 4 |
| Possible Orientia tsutsugamushi, Possible Leptospira sp |  | 5 | 5 |
| Probable JEV, Probable Orientia tsutsugamushi |  | 2 | 2 |
| Probable Chikungunya virus, Probable Orientia tsutsugamushi |  | 2 | 2 |
| Probable Chikungunya virus, Confirmed Orientia tsutsugamushi |  | 2 | 2 |
| Possible JEV, Possible Leptospira sp |  | 2 | 2 |
| Possible Dengue virus, Possible Orientia tsutsugamushi |  | 2 | 2 |
| Possible Dengue virus, Possible Leptospira sp |  | 2 | 2 |
| Probable Orientia tsutsugamushi, Probable Leptospira sp |  | 1 | 1 |
| Probable Orientia tsutsugamushi, Probable Chikungunya virus |  | 1 | 1 |
| Probable JEV, Probable Dengue virus, Probable Orientia tsutsugamushi |  | 1 | 1 |
| Probable JEV, Confirmed Orientia tsutsugamushi |  | 1 | 1 |
| Probable Dengue virus, Probable Orientia tsutsugamushi,Possible JEV |  | 1 | 1 |
| Probable Dengue virus, Probable Orientia tsutsugamushi, Possible JEV |  | 1 | 1 |
| Probable Chikungunya virus, Probable Orientia tsutsugamushi, Possible Leptospira sp |  | 1 | 1 |
| Probable Chikungunya virus, Possible Orientia tsutsugamushi |  | 1 | 1 |
| Possible JEV, Possible Dengue virus, Possible Orientia tsutsugamushi |  | 1 | 1 |
| Possible Japanese encephalitis virus, Possible Dengue virus, Possible Leptospira sp |  | 1 | 1 |
| Possible Dengue virus, Probable Orientia tsutsugamushi |  | 1 | 1 |
| Confirmed Orientia tsutsugamushi, Probable Dengue virus |  | 1 | 1 |
| Confirmed Orientia tsutsugamushi, Probable Chikungunya virus |  | 1 | 1 |
| Confirmed Orientia tsutsugamushi, Possible Japanese encephalitis, Possible Dengue virus, Possible Chikungunya virus |  | 1 | 1 |
| Confirmed Dengue virus, Possible Orientia tsutsugamushi |  | 1 | 1 |
| Confirmed Chikungunya virus, Probable Orientia tsutsugamushi |  | 1 | 1 |
| Confirmed Chikungunya virus, Confirmed Orientia tsutsugamushi |  | 1 | 1 |
| Probable SARS CoV-2 | 1 |  | 1 |
| Probable Mycobacterium tuberculosis | 1 |  | 1 |
| Probable Measles | 5 |  | 5 |
| Probable JEV, Probable WNV | 1 |  | 1 |
| Probable JEV | 29 |  | 29 |
| Probable Dengue virus | 11 |  | 11 |
| Probable Chikungunya virus | 6 |  | 6 |
| Possible Mycobacterium tuberculosis | 1 |  | 1 |
| Possible JEV, Probable Dengue virus | 1 |  | 1 |
| Possible JEV, Possible WNV, Probable Dengue virus | 1 |  | 1 |
| Possible JEV, Possible Dengue virus, Confirmed Chikungunya virus | 1 |  | 1 |
| Possible JEV, Possible Dengue virus | 9 |  | 9 |
| Possible JEV, Confirmed Dengue virus | 7 |  | 7 |
| Possible JEV, Confirmed Chikungunya virus | 1 |  | 1 |
| Possible Dengue virus, Possible WNV | 2 |  | 2 |
| Possible Chikungunya virus, Possible Dengue virus | 2 |  | 2 |
| Possible Chikungunya virus, Confirmed Dengue virus | 1 |  | 1 |
| Confirmed VZV, Possible JEV | 1 |  | 1 |
| Confirmed VZV | 1 |  | 1 |
| Confirmed Streptococcus pneumoniae | 3 |  | 3 |
| Confirmed *Salmonella typhi* | 1 |  | 1 |
| Confirmed Mycobacterium tuberculosis, Possible Dengue virus | 1 |  | 1 |
| Confirmed Mycobacterium tuberculosis | 1 |  | 1 |
| Confirmed Mumps | 2 |  | 2 |
| Confirmed MRSA | 1 |  | 1 |
| Confirmed JEV, Possible WNV | 1 |  | 1 |
| Confirmed JEV, Possible Dengue virus | 1 |  | 1 |
| Confirmed JEV | 5 |  | 5 |
| Confirmed HSV-2 | 1 |  | 1 |
| Confirmed HSV-1 | 4 |  | 4 |
| Confirmed *Haemophilus influenzae* | 1 |  | 1 |
| Confirmed Enterovirus | 3 |  | 3 |
| Confirmed Dengue virus, Possible WNV | 2 |  | 2 |
| Confirmed Dengue virus, Possible JEV | 1 |  | 1 |
| Confirmed Dengue virus | 5 |  | 5 |
| Confirmed Chikungunya virus, Probable WNV | 1 |  | 1 |
| Confirmed Chikungunya virus, Possible JEV | 1 |  | 1 |
| Confirmed Chikungunya virus, Possible Dengue virus | 1 |  | 1 |
| Confirmed Chikungunya virus, Confirmed Dengue virus | 1 |  | 1 |
| Confirmed Chikungunya virus | 3 |  | 3 |
| **Grand Total** | 122 | 193 | 315 |

**Table S9: Demographics and clinical characteristics of children with and without doxycycline-treatable causes (n=315)**

| **Baseline Clinical/ Laboratory variables** | **No patients (%)** |  | **Without doxycycline-treatable causes** | **With doxycycline-treatable causes** | **Total** | **p value** |
| --- | --- | --- | --- | --- | --- | --- |
| Total N (%) |  |  | 122 (38·7) | 193 (61·3) | 315 |  |
| Age (years) | 315 (100·0) | Median (IQR) | 5·0 (2·0–9·0) | 8·0 (4·3–11·0) | 7·0 (3·0–11·0) | **<0·001** |
| Gender | 315 (100·0) | Male | 76 (62·3) | 109 (56·5) | 185 (58·7) | 0·366 |
|  |  | Female | 46 (37·7) | 84 (43·5) | 130 (41·3) | |
| Duration of Illness (days) | 315 (100·0) | Median (IQR) | 4·0 (2·0–7·0) | 6·0 (4·0–8·0) | 5·0 (3·0–7·0) | **<0·001** |
| GCS score at admission | 265 (84·1) | 15 | 29 (23·8) | 58 (30·1) | 87 (27·6) | **0·024** |
|  |  | 8–14 | 45 (36·9) | 80 (41·5) | 125 (39·7) | |
|  |  | </=8 | 19 (15·6) | 34 (17·6) | 53 (16·8) |  |
| Seizure | 315 (100·0) | No | 33 (27·0) | 78 (40·4) | 111 (35·2) | **0·022** |
|  |  | Yes | 89 (73·0) | 115 (59·6) | 204 (64·8) | |
| Personality/ Behavioural  changes | 315 (100·0) | No | 99 (81·1) | 147 (76·2) | 246 (78·1) | 0·367 |
|  |  | Yes | 23 (18·9) | 46 (23·8) | 69 (21·9) |  |
| Irrelevant/Abnormal talk | 315 (100·0) | No | 106 (86·9) | 167 (86·5) | 273 (86·7) | 1 |
|  |  | Yes | 16 (13·1) | 26 (13·5) | 42 (13·3) |  |
| New abnormal speech | 315 (100·0) | No | 99 (81·1) | 148 (76·7) | 247 (78·4) | 0·425 |
|  |  | Yes | 23 (18·9) | 45 (23·3) | 68 (21·6) |  |
| Musculoskeletal symptoms | 315 (100·0) | No | 112 (91·8) | 177 (91·7) | 289 (91·7) | 1 |
|  |  | Yes | 10 (8·2) | 16 (8·3) | 26 (8·3) |  |
| Respiratory symptoms | 315 (100·0) | No | 102 (83·6) | 171 (88·6) | 273 (86·7) | 0·271 |
|  |  | Yes | 20 (16·4) | 22 (11·4) | 42 (13·3) |  |
| Diarrhoea | 315 (100·0) | No | 113 (92·6) | 177 (91·7) | 290 (92·1) | 0·938 |
|  |  | Yes | 9 (7·4) | 16 (8·3) | 25 (7·9) |  |
| Gastrointestinal symptoms | 315 (100·0) | No | 93 (76·2) | 124 (64·2) | 217 (68·9) | **0·035** |
|  |  | Yes | 29 (23·8) | 69 (35·8) | 98 (31·1) |  |
| Lymphadenopathy | 315 (100·0) | No | 119 (97·5) | 164 (85·0) | 283 (89·8) | **0·001** |
|  |  | Yes | 3 (2·5) | 29 (15·0) | 32 (10·2) |  |
| Oedema | 315 (100·0) | No | 113 (92·6) | 152 (78·8) | 265 (84·1) | **0·002** |
|  |  | Yes | 9 (7·4) | 41 (21·2) | 50 (15·9) |  |
| Conjunctival involvement | 315 (100·0) | No | 114 (93·4) | 170 (88·1) | 284 (90·2) | 0·173 |
|  |  | Yes | 8 (6·6) | 23 (11·9) | 31 (9·8) |  |
| Rash (including eschar and  purpura fulminans) | 315 (100·0) | No | 107 (87·7) | 156 (80·8) | 263 (83·5) | 0·148 |
|  |  | Yes | 15 (12·3) | 37 (19·2) | 52 (16·5) |  |
| Hepatomegaly | 315 (100·0) | 0 | 96 (78·7) | 104 (53·9) | 200 (63·5) | **<0·001** |
|  |  | 1 | 26 (21·3) | 89 (46·1) | 115 (36·5) | |
| Splenomegaly | 315 (100·0) | 0 | 114 (93·4) | 163 (84·5) | 277 (87·9) | **0·027** |
|  |  | 1 | 8 (6·6) | 30 (15·5) | 38 (12·1) |  |
| Hepatosplenomegaly | 315 (100·0) | No | 114 (93·4) | 163 (84·5) | 277 (87·9) | **0·027** |
|  |  | Yes | 8 (6·6) | 30 (15·5) | 38 (12·1) |  |
| Presence of cerebellar signs | 315 (100·0) | No | 112 (91·8) | 165 (85·5) | 277 (87·9) | 0·134 |
|  |  | Yes | 10 (8·2) | 28 (14·5) | 38 (12·1) |  |
| Signs of meningeal irritation | 315 (100·0) | No | 84 (68·9) | 113 (58·5) | 197 (62·5) | 0·085 |
|  |  | Yes | 38 (31·1) | 80 (41·5) | 118 (37·5) | |
| Cranial Nerve Abnormality | 315 (100·0) | No | 112 (91·8) | 174 (90·2) | 286 (90·8) | 0·77 |
|  |  | Yes | 10 (8·2) | 19 (9·8) | 29 (9·2) |  |
| Involuntary movements | 315 (100·0) | No | 112 (91·8) | 178 (92·2) | 290 (92·1) | 1 |
|  |  | Yes | 10 (8·2) | 15 (7·8) | 25 (7·9) |  |
| Haemoglobin(g/dL) | 310 (98·4) | Median (IQR) | 10·9 (9·5–12·6) | 10·5 (9·4–11·3) | 10·6 (9·4–11·7) | **0·015** |
| Total white blood cell count  (× 10^9^/L) | 313 (99·4) | Median (IQR) | 10·7 (7·2–14·0) | 11·3 (7·6–14·4) | 11·0 (7·5–14·4) | 0·49 |
| Absolute Lymphocyte Count  (× 10^9^/L) | 313 (99·4) | Median (IQR) | 2·4 (1·5–3·8) | 3·2 (2·0–5·2) | 2·8 (1·8–4·7) | **<0·001** |
| Absolute Neutrophil Count  (× 10^9^/L) | 312 (99·0) | Median (IQR) | 6·5 (4·4–9·8) | 6·4 (4·2–9·2) | 6·4 (4·3–9·5) | 0·406 |
| Neutrophil/ Lymphocyte ratio | 312 (99·0) | Median (IQR) | 2·7 (1·5–5·3) | 1·9 (1·1–3·2) | 2·1 (1·2–3·8) | **<0·001** |
| Platelets count (× 10^9^/L) | 312 (99·0) | Median (IQR) | 238·0 (131·0–348·0) | 145·0 (76·0–224·0) | 174·5 (93·8–285·2) | **<0·001** |
| Total Bilirubin(mg/dL) | 276 (87·6) | Median (IQR) | 0·3 (0·2–0·5) | 0·4 (0·3–1·1) | 0·4 (0·2–0·9) | **0·001** |
| Direct Bilirubin (mg/dL) | 277 (87·9) | Median (IQR) | 0·1 (0·1–0·2) | 0·2 (0·1–0·8) | 0·2 (0·1–0·6) | **<0·001** |
| Aspartate transaminase (AST) (IU/L) | 300 (95·2) | Median (IQR) | 39·7 (26·7–88·2) | 91·7 (49·7–191·6) | 69·5 (35·0–155·0) | **<0·001** |
| Alanine transaminase (ALT)  (IU/L) | 300 (95·2) | Median (IQR) | 24·0 (13·6–43·8) | 60·0 (33·9–113·9) | 45·8 (20·7–97·0) | **<0·001** |
| Transaminitis | 300 (95·2) | No | 78 (63·9) | 78 (40·4) | 156 (49·5) | **<0·001** |
|  |  | Yes | 34 (27·9) | 110 (57·0) | 144 (45·7) | |
| Total Protein (g/dL) | 282 (89·5) | Median (IQR) | 6·5 (5·6–7·1) | 6·0 (5·4–6·6) | 6·2 (5·4–6·9) | **0·02** |
| Serum albumin (g/dL) | 282 (89·5) | Median (IQR) | 3·8 (3·0–4·2) | 2·9 (2·6–3·5) | 3·2 (2·7–3·9) | **<0·001** |
| Urea (mg/dL) | 281 (89·2) | Median (IQR) | 26·1 (18·0–37·6) | 24·9 (18·0–33·0) | 25·0 (18·0–35·0) | 0·174 |
| Creatinine (mg/dL) | 309 (98·1) | Median (IQR) | 0·5 (0·3–0·7) | 0·4 (0·3–0·5) | 0·4 (0·3–0·6) | 0·245 |
| Sodium (mEq/L) | 313 (99·4) | Median (IQR) | 136·0 (133·0–139·0) | 136·0 (133·0–139·0) | 136·0 (133·0–139·0) | 0·687 |
| Potassium (mEq/L) | 313 (99·4) | Median (IQR) | 4·5 (4·0–4·7) | 4·4 (4·0–4·8) | 4·4 (4·0–4·8) | 0·657 |
| Chloride (mEq/L) | 308 (97·8) | Median (IQR) | 102·0 (98·2–106·4) | 101·5 (97·6–105·2) | 101·5 (97·8–105·6) | 0·327 |
| CSF total leucocyte count  (cells/µL) | 284 (90·2) | Median (IQR) | 3·0 (0·0–17·0) | 13·0 (4·0–49·0) | 7·5 (2·0–38·2) | **<0·001** |
| CSF lymphocyte count  (cells/µL) | 284 (90·2) | Median (IQR) | 3·0 (0·0–13·0) | 11·0 (3·0–36·0) | 7·0 (2·0–27·0) | **<0·001** |
| CSF neutrophil count  (cells/µL) | 284 (90·2) | Median (IQR) | 0·0 (0·0–0·0) | 0·0 (0·0–3·0) | 0·0 (0·0–2·0) | 0·112 |
| CSF pleocytosis | 284 (90·2) | No | 68 (55·7) | 50 (25·9) | 118 (37·5) | **<0·001** |
|  |  | Yes | 43 (35·2) | 123 (63·7) | 166 (52·7) | |
| CSF protein concentration  (mg/dL) | 287 (91·1) | Median (IQR) | 24·8 (16·2–46·0) | 54·5 (33·4–86·6) | 46·0 (22·0–76·7) | **<0·001** |
| CSF Glucose (mg/dL) | 289 (91·7) | Median (IQR) | 61·0 (51·0–70·0) | 55·6 (49·1–64·7) | 57·7 (49·6–67·0) | **0·018** |
| Duration of hospitalisation (days) | 300 (95·2) | Median (IQR) | 9·5 (7·0–16·8) | 9·0 (7·0–13·0) | 9·0 (7·0–14·0) | 0·365 |
| Managed in intensive care unit during  hospitalisation | 315 (100·0) | No | 53 (43·4) | 81 (42·0) | 134 (42·5) | 0·888 |
|  |  | Yes | 69 (56·6) | 112 (58·0) | 181 (57·5) | |
| Liverpool Outcome Score | 243 (77·1) | Unfavorable | 35 (28·7) | 48 (24·9) | 83 (26·3) | **0·002** |
|  |  | Favorable | 48 (39·3) | 112 (58·0) | 160 (50·8) | |

**Figure S6: No. (%) of missing values in the dataset used for developing the model for scrub typhus diagnosis
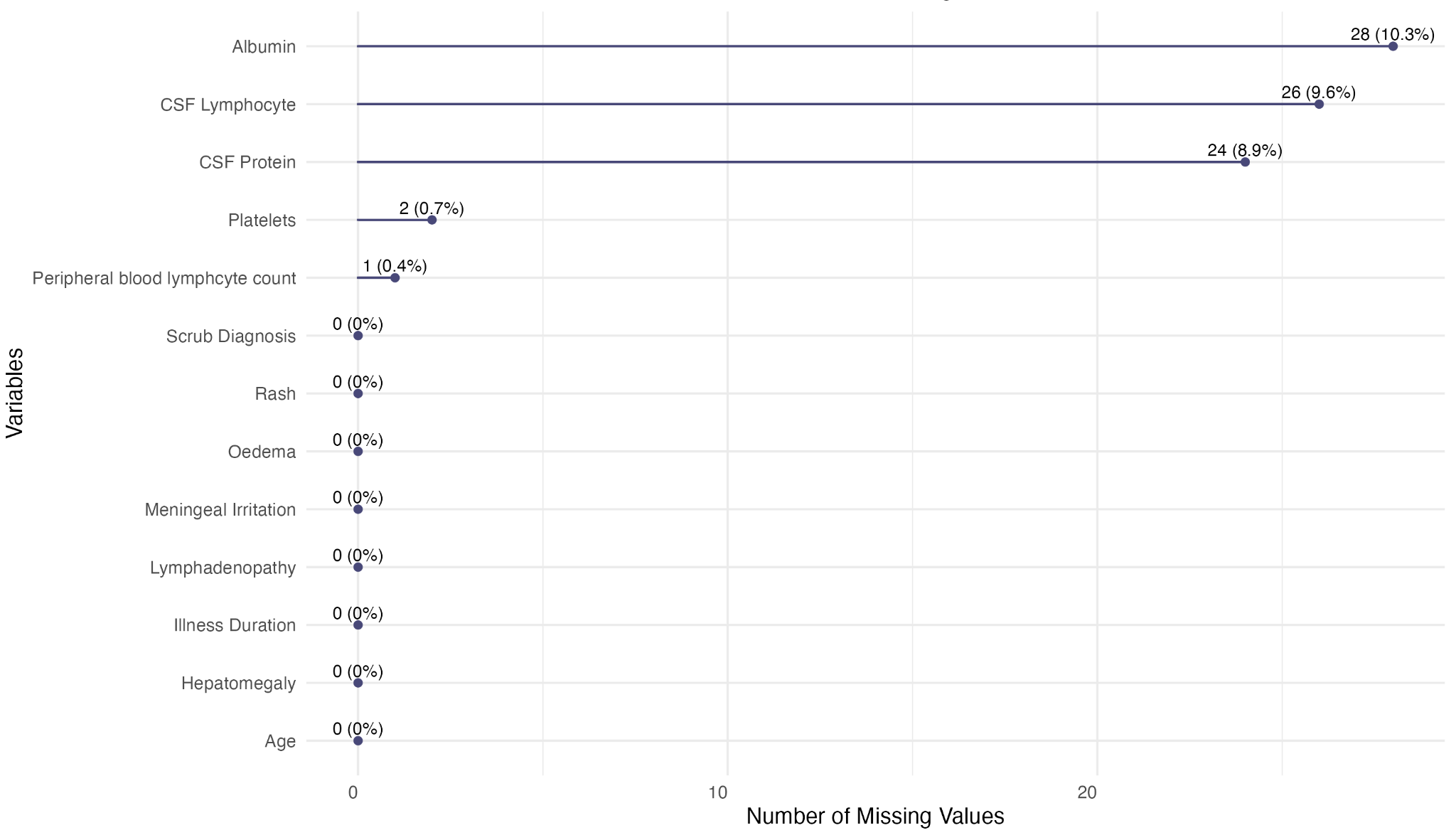
**

Missing data are presented as numbers (percentage). Missing values ranged between 1 (0.4%) to 28 (10.3%).

**Figure S7: No. (%) of missing values in the dataset used for developing the model for doxycycline-treatable causes**

**
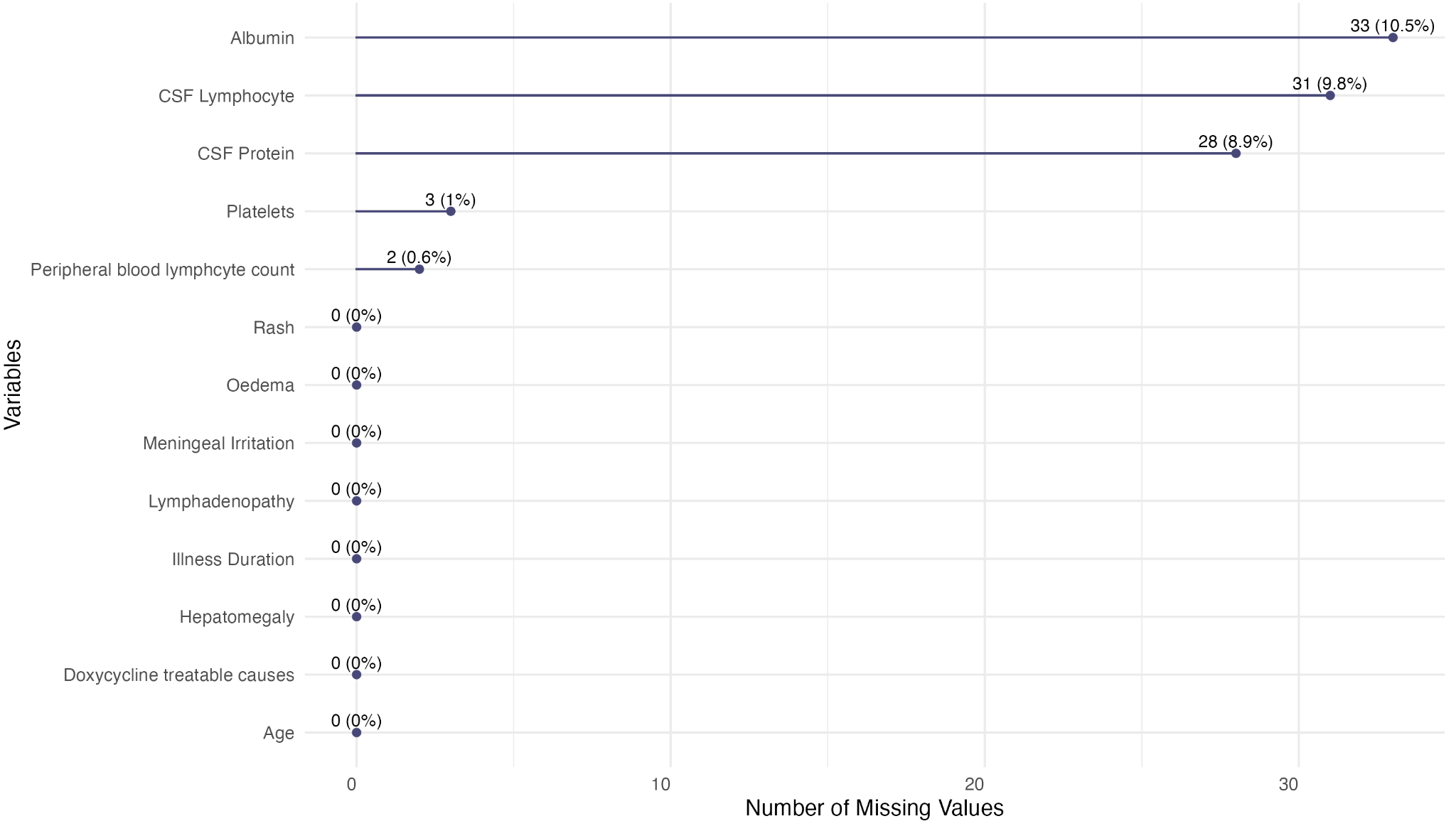
**

Missing data are presented as numbers (percentage). Missing values ranged between 2 (0.6%) to 33 (10.5%).

**Table S10: Univariable odds ratios for risk of scrub typhus and doxycycline-treatable cause**

| **Predictor** | **Scrub typhus,**  **OR (95% CI)** | **p value** | **Doxycycline-treatable causes,**  **OR (95% CI)** | **p value** |
| --- | --- | --- | --- | --- |
| Age (years) | 1·07 (1·01–1·12) | **0.01** | 1·09 (1·04–1·15) | **0·001** |
| Duration of illness (days) | 1·02 (0·98 –1·07) | 0·32 | 1·04 (0·99–1·10) | 0·089 |
| Hepatomegaly | 3·91 (2·31–6·61) | <**0·0001** | 3·16 (1·88–5·30) | **<0·0001** |
| Lymphadenopathy | 7·33 (2·45–21·92) | **0·0004** | 7·01 (2·09–23·56) | **0·002** |
| Oedema | 4·33 (2·02–9·25) | **0·0002** | 3·39 (1·58–7·25) | **0·002** |
| Rash | 1·23 (0·63–2·39) | 0·54 | 1·69 (0·88–3·24) | 0·112 |
| Signs of meningeal irritation | 1·53 (0·93–2·51) | 0·10 | 1·56 (0·97–2·53) | 0·067 |
| Absolute Lymphocyte Count (×10^9^/L) | 1·31 (1·17–1·47) | <**0·0001** | 1·19 (1·08–1·33) | **0·001** |
| Platelets count (×10^9^/L) | 0·99 (0·99–1·00) | <**0·0001** | 1·00 (0·99–1·00) | <**0·0001** |
| Serum albumin (g/dL) | 0·24 (0·16–0·37) | <**0·0001** | 0·37 (0·26–0·53) | <**0·0001** |
| CSF lymphocyte count (cells/µL) | 1·00 (1·00–1·00) | 0·60 | 1·00 (1·00–1·00) | 0·401 |
| CSF protein concentration (mg/dL) | 1·02 (1·01–1·02) | <**0·0001** | 1·02 (1·01–1·02) | <**0·0001** |

**Details of additional models developed**

Two additional models were developed for diagnosis of scrub typhus and doxycycline-treatable causes of AES, tailored for different clinical scenarios: a “Presentation-at-hospital”, which used only clinical variables for immediate application at hospital presentation, and a “post-lumbar puncture (post-LP) model” that incorporated CSF lymphocyte count and CSF protein concentration alongside the preselected clinical and laboratory variables· The predictors for these models are outlined in Tables 7 and 8· These models were simplified into point scoring systems· Scores for the scrub typhus presentation-at-hospital model ranged from 0 to 9, predicting probabilities of outcomes between 24% and 97%, while that for the post-LP model ranged from 0 to 20, predicting probabilities between 1·6% and 99·7%. Similarly, scores for the presentation-at-hospital model for doxycycline-treatable causes ranged from 0 to 9, with predicted probabilities between 36% and 98%, and for the post-LP model ranged from 0 to 15, predicting probabilities from 17% to 99·7%. The scoring systems and estimated risks for outcomes based on these scores are detailed in Tables 10 to 13. Calibration plots (Fig. 6) demonstrated strong agreement between observed and predicted probabilities across all models. Post-imputation and internal validation, the models exhibited robust performance metrics, including strong discrimination and calibration, as shown in Table 14.

**Table S11: Multivariable presentation-at-hospital models, adjusted for shrinkage**

| **Predictor** | **Comparison** | **Adjusted Odds ratio** | |
| --- | --- | --- | --- |
|  |  | **Scrub typhus** | **Doxycycline-treatable causes** |
| Intercept, log odds ratio (s.e.) |  | 0·23 (0·03) | 0·39 (0·26) |
| Age (years) | <6 | 1·00 (reference) | 1·00 (reference) |
|  | ≥ 6 | 1·20 (1·07–1·34) | 1·23 (1·10–1·37) |
| Illness duration (days) | ≤ 5 | 1·00 (reference) | 1·00 (reference) |
|  | >5 | 1·08 (0·93–1·25) | 1·13 (0·98–1·30) |
| Oedema | No | 1·00 (reference) | 1·00 (reference) |
|  | Yes | 2·83 (1·36–5·89) | 2·38 (1·17–4·84) |
| Lymphadenopathy | No | 1·00 (reference) | 1·00 (reference) |
|  | Yes | 4·64 (1·70–12·64) | 4·28 (1·46–12·50) |
| Hepatomegaly | No | 1·00 (reference) | 1·00 (reference) |
|  | Yes | 2·82 (1·71–4·65) | 2·22 (1·37–3·60) |

**Table S12: Multivariable post-LP models, adjusted for shrinkage**

| **Predictor** | **Comparison** | **Adjusted Odds ratio** |
| --- | --- | --- |
| **Model for scrub typhus** | | |
| Intercept, log odds ratio (s.e.) | | 1·37 (0·82) |
| Age (years) | <6 | 1·00 (reference) |
|  | ≥ 6 | 1·28 (1·11–1·47) |
| Lymphadenopathy | No | 1·00 (reference) |
|  | Yes | 5·30 (1·77–15·83) |
| Hepatomegaly | No | 1.00 (reference) |
|  | Yes | 1·81 (1·00–3·27) |
| Lymphocyte count (× 10^9/L) | <1·5 | 1·00 (reference) |
|  | 1·5-3 | 1·22 (1·11–1·33) |
|  | >3 | 2·06 (1·46–2·90) |
| Platelet count (× 10^9/L) | ≤ 150 | 1·04 (1·00–1·08) |
|  | >150 | 1·00 (reference) |
| Serum albumin (mg/dL) | ≤3 | 1·95 (1·49–2·56) |
|  | 3-3·5 | 1·37 (1·07–1·46) |
|  | >3·5 | 1·00 (reference) |
| CSF lymphocyte (cells/ul) | 0-100 | 1·05 (0·99–1·11) |
|  | >100 | 1·00 (reference) |
| CSF protein (mg/dL) | <50 | 1·00 (reference) |
|  | 50-100 | 1·06 (1·03–1·10) |
|  | >100 | 1·16 (1·07–1·26) |
| **Model for doxycycline-treatable causes** | | |
| Intercept, log odds ratio (s.e.) | | 1·12 (0·73) |
| Age (years) | <6 | 1·00 (reference) |
|  | ≥ 6 | 1·23 (1·10–1·39) |
| Lymphadenopathy | No | 1·00 (reference) |
|  | Yes | 3·22 (1·13–9·16) |
| Hepatomegaly | No | 1·00 (reference) |
|  | Yes | 1·89 (1·16–3·07) |
| Lymphocyte count (× 10^9/L) | ≤3 | 1·00 (reference) |
|  | >3 | 1·44 (1·42–2·42) |
| Platelet count (× 10^9/L) | ≤ 150 | 1·04 (1·01–1·07) |
|  | >150 | 1·00 (reference) |
| CSF lymphocyte (cells/ul) | 0-100 | 1·05 (0·99–1·11) |
|  | >100 | 1·00 (reference) |
| CSF protein (mg/dL) | <50 | 1·00 (reference) |
|  | >50-100 | 1·07 (1·03–1·11) |
|  | >100 | 1·17 (1·06–1·25) |

**Table S13: Scoring system for probability of diagnosis of scrub typhus and doxycycline-treatable causes in children with AES, using presentation-at-hospital models**

| **Predictor** | **Category** | **Score** |
| --- | --- | --- |
| Age (years) | <6 | 0 |
|  | >/=6 | 1 |
| Illness Duration (days) | </=5 | 0 |
|  | >5 | 1 |
| Oedema | No | 0 |
|  | Yes | 2 |
| Lymphadenopathy | No | 0 |
|  | Yes | 3 |
| Hepatomegaly | No | 0 |
|  | Yes | 2 |

**Table S14**: **Scoring system for probability of diagnosis of scrub typhus and doxycycline-treatable causes in children with AES, using post-LP models**

| **Predictor** | **Category** | **Point score** |
| --- | --- | --- |
| **Model for scrub typhus** | | |
| Age (years) | <6 | 0 |
|  | ≥ 6 | 2 |
| Lymphadenopathy | No | 0 |
|  | Yes | 3 |
| Hepatomegaly | No | 0 |
|  | Yes | 1 |
| Lymphocyte count (× 10^9/L) | <1·5 | 0 |
|  | 1·5-3 | 1 |
|  | >3 | 4 |
| Platelet count (× 10^9/L) | ≤ 150 | 2 |
|  | >150 | 0 |
| Serum albumin (mg/dL) | ≤3 | 2 |
|  | 3-3·5 | 1 |
|  | >3·5 | 0 |
| CSF lymphocyte (cells/ul) | 0-100 | 2 |
|  | >100 | 0 |
| CSF protein (mg/dL) | <50 | 0 |
|  | 50-100 | 1 |
|  | >100 | 4 |
| **Model for doxycycline-treatable causes** | | |
| Age (years) | <6 | 0 |
|  | ≥ 6 | 2 |
| Lymphadenopathy | No | 0 |
|  | Yes | 3 |
| Hepatomegaly | No | 0 |
|  | Yes | 1 |
| Lymphocyte count (× 10^9/L) | ≤3 | 0 |
|  | >3 | 2 |
| Platelet count (× 10^9/L) | ≤ 150 | 1 |
|  | >150 | 0 |
| CSF lymphocyte (cells/ul) | 0-100 | 2 |
|  | >100 | 0 |
| CSF protein (mg/dL) | ≤50 | 0 |
|  | >50-100 | 1 |
|  | >100 | 4 |

**Table S15: Estimate of risk based on scores using presentation-at-hospital models**

| **Point score** | **Estimate of risk of scrub typhus (%)** | **Estimate of risk of doxycycline-treatable causes (%)** |
| --- | --- | --- |
| 0 | 23·8 | 35·9 |
| 1 | 34·0 | 48·0 |
| 2 | 45·9 | 60·3 |
| 3 | 58·3 | 71·5 |
| 4 | 69·7 | 80·5 |
| 5 | 79·2 | 87·2 |
| 6 | 86·2 | 91·8 |
| 7 | 91·2 | 94·9 |
| 8 | 94·5 | 96·8 |
| 9 | 96·6 | 98·1 |

**Table S16: Estimate of risk based on scores using post-LP models**

| **Point score** | **Estimate of risk of scrub typhus (%)** | **Estimate of risk of doxycycline-treatable causes (%)** |
| --- | --- | --- |
| 0 | 1·6 | 17·2 |
| 1 | 2·7 | 25·5 |
| 2 | 4·3 | 36·1 |
| 3 | 7·0 | 48·2 |
| 4 | 11·0 | 60·6 |
| 5 | 16·9 | 71·7 |
| 6 | 25·1 | 80·7 |
| 7 | 35·6 | 87·3 |
| 8 | 47·7 | 91·9 |
| 9 | 60·1 | 94·9 |
| 10 | 71·3 | 96·9 |
| 11 | 80·3 | 98·1 |
| 12 | 87·1 | 98·8 |
| 13 | 91·7 | 99·3 |
| 14 | 94·8 | 99·6 |
| 15 | 96·8 | 99·7 |
| 16 | 98·0 | .. |
| 17 | 98·8 | .. |
| 18 | 99·3 | .. |
| 19 | 99·6 | .. |
| 20 | 99·7 | .. |

**Table S17: Model performance**

| **Models** | | **C-statistics** | | **C-slope** | | **CITL** | |
| --- | --- | --- | --- | --- | --- | --- | --- |
|  |  | Unadjusted | Adjusted | Unadjusted | Adjusted | Unadjusted | Adjusted |
| **Primary model** | Scrub typhus | 0·86 (0·82–0·90) | 0·83 (0·78–0·87) | 1·00 (0·77–1·26) | 0·85 (0·82–0·88) | 0·00 (-0·31–0·30) | -0·03 (-0·06–0·00) |
|  | Doxycycline-treatable causes | 0·80 (0·75–0·84) | 0·75 (0·70–0·81) | 1·00 (0·75–1·27) | 0·83 (0·78–0·87) | 0·00 (-0·26–0·26) | 0·05 (0·02–0·09) |
| **Presentation-at-hospital model** | Scrub typhus | 0·75 (0·70–0·81) | 0·73 (0·67–0·79) | 1·00 (0·71–1·31) | 0·88 (0·83–0·92) | 0·00 (-0·27–0·27) | -0·02 (-0·05–0·01) |
|  | Doxycycline-treatable causes | 0·73 (0·68–0·79) | 0·70 (0·64–0·76) | 1·00 (0·71–1·32) | 0·86 (0·80–0·92) | 0·00 (-0·25–0·25) | 0·05 (0·01–0·09) |
| **Post-LP model** | Scrub typhus | 0·88 (0·84–0·92) | 0·84 (0·79–0·88) | 1·00 (0·78–1·25) | 0·83 (0·80–0·86) | 0·00 (-0·32–0·32) | -0·03 (-0·07–0·01) |
|  | Doxycycline-treatable causes | 0·82 (0·77–0·87) | 0·78 (0·73–0·83) | 1·00 (0·77–1·26) | 0·81 (0·77–0·85) | 0·00 (-0·27–0·27) | 0·05 (0·01–0·09) |

C-statistic measures the model’s ability to discriminate between cases and non-cases. Values range from 0.5 (no discrimination) to 1.0 (perfect discrimination).
C-slope assesses agreement between predicted and observed risks. A value of 1 indicates perfect calibration; values <1 suggest overfitting. CITL indicates whether the model systematically over- or underpredicts risk. A CITL of 0 represents perfect calibration; negative values indicate overestimation, and positive values indicate underestimation of risk.^11^

For example, adjusted CITL of -0.03 (-0.06 to 0) in the primary model for scrub typhus indicates that the model slightly overestimates risk on average, but the CI includes 0, suggesting the overestimation is minor and may not be statistically significant. Similarly, CITL of 0.05 (0.02 to 0.09) indicates that the model slightly underestimates risk on average, and is statistically significant, indicating a small but consistent tendency to predict lower probabilities than the actual observed risk.

**Figure S8: Calibration plots for the models**

|  | Scrub typhus | Doxycycline-treatable causes |
| --- | --- | --- |
| Primary model | 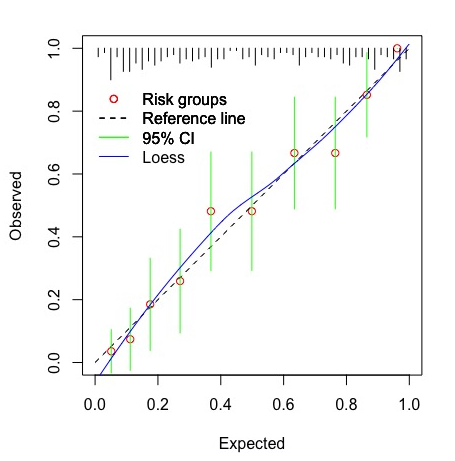 | 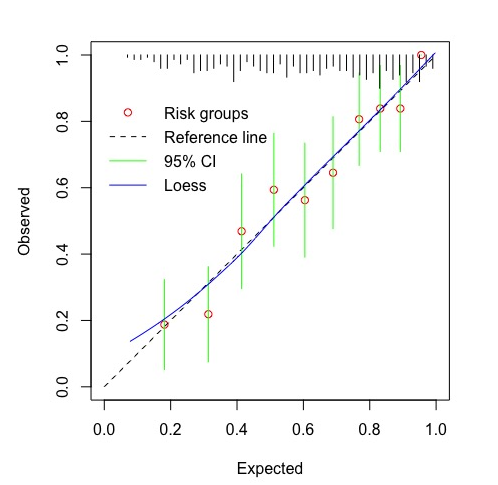 |
| Presentation-at-hospital model | 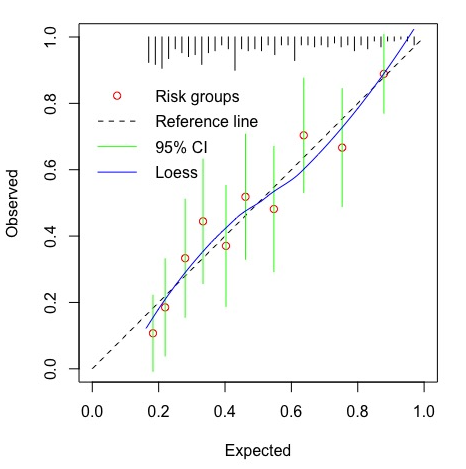 | 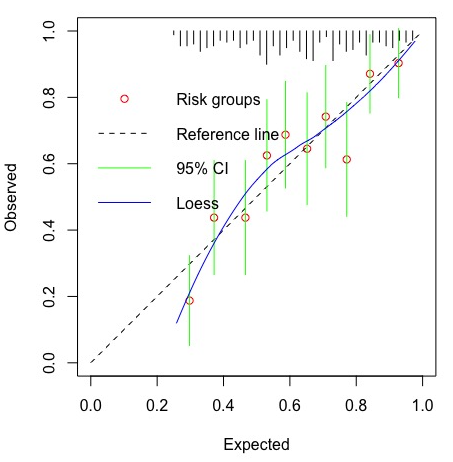 |
| Post-LP model | 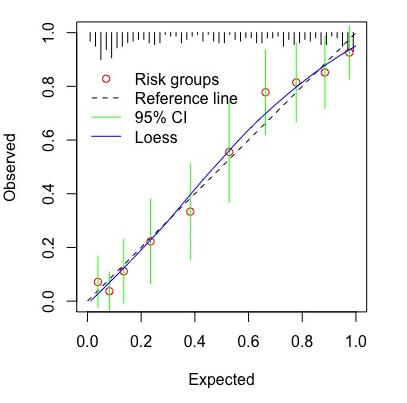 | 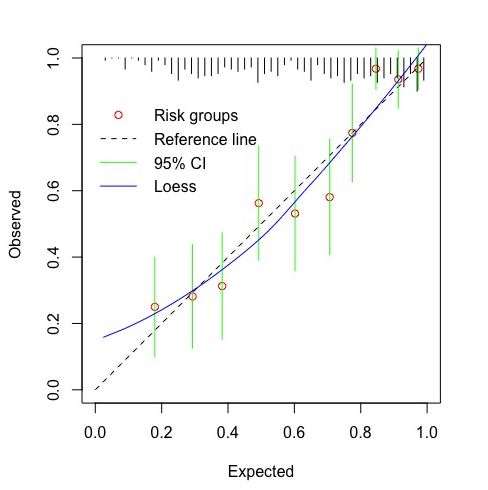 |

**References**

1. Calvert AE, Dixon KL, Delorey MJ, Blair CD, Roehrig JT. Development of a Small Animal Peripheral Challenge Model of Japanese Encephalitis Virus Using Interferon Deficient AG129 Mice and the SA14-14-2 Vaccine Virus Strain. *Vaccine*. 2014;32:258–64.
2. Johnson BW, Russell BJ, Lanciotti RS. Serotype-specific detection of dengue viruses in a fourplex real-time reverse transcriptase PCR assay. *J Clin Microbiol*. 2005;43:4977–83.
3. Lanciotti RS, Kosoy OL, Laven JJ, Panella AJ, Velez JO, Lambert AJ, et al. Chikungunya Virus in US Travelers Returning from India, 2006. *Emerg Infect Dis. 2007*;13:764–7.
4. Piqueur MA, Verstrepen WA, Bruynseels P, Mertens AH. Improvement of a real-time RT-PCR assay for the detection of enterovirus RNA. *Virol J*. 2009 7;6:95.
5. Barzon L, Murer L, Pacenti M, Biasolo MA, Della Vella M, Benetti E, Zanon GF, Palù G. Investigation of intrarenal viral infections in kidney transplant recipients unveils an association between parvovirus B19 and chronic allograft injury. *J Infect Dis*. 2009;199:372–80.
6. Carvalho M da GS, Tondella ML, McCaustland K, Weidlich L, McGee L, Mayer LW, et al. Evaluation and Improvement of Real-Time PCR Assays Targeting lytA, ply, and psaA Genes for Detection of Pneumococcal DNA. *J Clin Microbiol*. 2007;45:2460–6.
7. Wang X, Mair R, Hatcher C, Theodore MJ, Edmond K, Wu HM, et al. Detection of bacterial pathogens in Mongolia meningitis surveillance with a new real-time PCR assay to detect Haemophilus influenzae. *Int J Med Microbiol*. 2011;301:303–9.
8. Corless CE, Guiver M, Borrow R, Edwards-Jones V, Fox AJ, Kaczmarski EB. Simultaneous Detection of Neisseria meningitidis, Haemophilus influenzae, and Streptococcus pneumoniae in Suspected Cases of Meningitis and Septicemia Using Real-Time PCR. *J Clin Microbiol*. 2001;39:1553–8.
9. Savelkoul PHM, Catsburg A, Mulder S, Oostendorp L, Schirm J, Wilke H, et al. Detection of Mycobacterium tuberculosis complex with Real Time PCR:Comparison of different primer-probe sets based on the IS6110 element. *J Microbiol Methods*. 2006;66:177–80.
10. Jiang J, Chan TC, Temenak JJ, Dasch GA, Ching WM, Richards AL. Development of a quantitative real-time polymerase chain reaction assay specific for Orientia tsutsugamushi. Am J Trop Med Hyg. 2004;70:351–6.
11. [Steyerberg EW. Clinical Prediction Models: A Practical Approach to Development, Validation, and Updating.](https://www.zotero.org/google-docs/?broken=q0lU8Q) 2nd ed. [Germany: Springer International Publishing, 2019](https://www.zotero.org/google-docs/?broken=q0lU8Q) (accessed Nov 20, 2024).
